# The BCL-2 inhibitor ABT-199/venetoclax synergizes with proteasome inhibition via transactivation of the MCL-1 antagonist NOXA

**DOI:** 10.1038/s41420-022-01009-1

**Published:** 2022-04-20

**Authors:** Sandra Weller, Astrid Toennießen, Benjamin Schaefer, Tobias Beigl, Alina Muenchow, Kathrin Böpple, Ute Hofmann, Bernhard F. Gillissen, Walter E. Aulitzky, Hans-Georg Kopp, Frank Essmann

**Affiliations:** 1 Dr. Margarete-Fischer-Bosch Institute of Clinical Pharmacology and University of Tuebingen, Auerbachstr. 112, 70376 Stuttgart, Germany; 2 Department of Hematology, Oncology and Tumor Immunology, Charité - Universitätsmedizin Berlin, Corporate Member of Freie Universität Berlin, Humboldt-Universität zu Berlin, and Berlin Institute of Health, Berlin, Germany; 3 Robert-Bosch-Hospital, Department of Hematology, Oncology and Palliative Medicine, Auberbachstr. 110, 70376 Stuttgart, Germany; 4 Robert Bosch Center for Tumor Diseases and Robert-Bosch-Hospital, Department of Molecular Oncology, Auerbachstr. 110, 70376 Stuttgart, Germany; 5 Robert Bosch Center for Tumor Diseases, Auerbachstr. 112, 70376 Stuttgart, Germany

**Keywords:** Apoptosis, Cancer therapeutic resistance, Metabolomics, Cancer therapy

## Abstract

Enhanced expression of anti-apoptotic B-cell lymphoma 2 (BCL-2) protein is frequent in cancer. Targeting of BCL-2 with the specific inhibitor ABT-199 (Venetoclax) has significant clinical activity in malignant diseases such as chronic lymphocytic leukemia and multiple myeloma. The small molecule drug ABT-199 mimics the pro-apoptotic BCL-2 homology domain 3 of BH3-only proteins and blocks the hydrophobic BC-groove in BCL-2. We have previously shown that ABT-199 synergizes with the proteasome inhibitor (PI) bortezomib in soft tissue sarcoma derived cells and cell lines to induce apoptosis. Synergistic apoptosis induction relies on the pore-forming effector BAX and expression of the pro-apoptotic BH3-only protein NOXA. Bortezomib augments expression of NOXA by blocking its proteasomal degradation. Interestingly, shown here for the first time, expression of NOXA is strongly enhanced by ABT-199 induced integrated stress response (ISR). ISR transcription factors ATF3 & ATF4 mediate transactivation of the BH3-only protein NOXA which specifically inhibits the anti-apoptotic MCL-1. Thus, NOXA potentiates the efficacy of the BCL-2 inhibitor ABT-199 by simultaneous inhibition of MCL-1. Hence, ABT-199 has a double impact by directly blocking anti-apoptotic BCL-2 and inhibiting MCL-1 via transactivated NOXA. By preventing degradation of NOXA PIs synergize with ABT-199. Synergism of ABT-199 and PIs therefore occurs on several, previously unexpected levels. This finding should prompt clinical evaluation of combinatorial regimens in further malignancies.

## Introduction

Mitochondria are the lead actors of the intrinsic apoptosis pathway and they are staged by pro- and anti-apoptotic members of the BCL-2 protein family. BCL-2 kinship is rooted to the presence of at least one of the four distinct BCL-2 homology (BH1-4) domains. The exclusive presence of the BH3-domain distinguishes the pro-apoptotic “BH3-only” proteins (BAD, BID, BIM, NOXA, PUMA) from their pro-apoptotic multidomain protein (MDP) siblings BAX, BAK, and BOK, that share BH1-4 [1]. BAX, BAK, and BOK are effector proteins that upon activation undergo conformational changes and oligomerize in the mitochondrial outer membrane (MOM). Upon oligomerization the effectors mediate MOM permeabilization (MOMP) thus releasing cytochrome c resulting in activation of caspases and cellular demise. Yet, a third clan of the BCL-2 family, the anti-apoptotic “BCL-2-like” proteins (BCL-2, BCL-x_L_, BCL-w, MCL-1, A1), antagonizes both pro-apoptotic clans, the BH3-only proteins and MDPs BAX and BAK, by accommodating their BH3-domain in a hydrophobic groove. Hence, BH3-only proteins neutralize the capacity of anti-apoptotic proteins to antagonize the effectors BAX and BAK and promote cell death [2–4]. The amino acid composition of the hydrophobic groove and the BH3-domain sequence determine the specific interaction, i.e. the antagonizing effect of BCL-2-like proteins on certain pro-apoptotic clan members, generating two signaling axes: the BCL-2-BAX and the MCL-1-BAK axis.

A unique class of small molecule BCL-2 inhibitors/BH3-mimetics [5] that specifically block anti-apoptotic BCL-2 proteins has been developed and the BCL-2 specific ABT-199/Venetoclax showed efficacy in the treatment of chronic lymphocytic leukemia [6, 7]. ABT-199 is effective in hematopoietic malignancies: Multiple Myeloma (MM) and acute and chronic myeloid leukemia (AML and CML) overexpressing BCL-2 [6, 8]. Consequently, efficacy of ABT-199 tends to be higher in Multiple Myeloma with a high ratio of BCL-2 relative to BCL-x_L_ or MCL-1 [9]. Not surprisingly, MCL-1 mediates (acquired) resistance to ABT-199 [10]. Combined application of ABT-199 with an MCL-1 specific inhibitor (S63845) thus is effective in high MCL-1 expressing MM cells [11]. Meanwhile, several MCL-1 specific BH3-mimetics have been published, e.g. A-1210477 [12], S63845 [13], AZD5991 [14], AMG-176 [15], and AMG-397 [16]. The physiologic opponents of MCL-1 are promiscuous BH3-only proteins BIM, PUMA, and caspase-cleaved truncated BID (tBID) along with the MCL-1 antagonist NOXA [17]. Expression of *PMAIP1*/NOXA is induced by the tumor suppressor protein TP53 [18] and NOXA is relevant for DNA-damage induced apoptosis [19]. Recent evidence shows that interaction of NOXA with MCL-1 mediates ubiquitylation of the MCL-1:NOXA complex at mitochondria and subsequent proteasomal degradation [20, 21].

Another class of anti-cancer drugs are PIs. Improvement of the prototypical PI bortezomib (BTZ) [22] has yielded effective drugs for the therapy of MM and mantle cell lymphoma in particular [23]. PIs in clinical use, such as ixazomib (IXZ), carfilzomib (CFZ) and marizomib (MRZ), differ in pharmacokinetic and specificity towards the active subunits (β_1_, β_2_, β_5_) in the proteasome. Each PI targets the chymotrypsin-like activity of β_5_. However, BTZ and IXZ also inhibit the caspase-like activity of β_1_ while at increased concentration CFZ also blocks the trypsin-like activity of the β_2_ subunit [24]. Proteasome inhibition induces accumulation of non-functional and misfolded proteins and activates cellular stress response pathways: i) the ER-lumen prompts the unfolded protein response (UPR), and ii) the cytosol the heat shock response (HSR), while iii) integrated stress response (ISR) reacts to both ER-lumen and cytosol. The ISR funnels diverse stress signals and reduces global protein synthesis while allowing translation of specific mRNAs or open reading frames (ORFs) from alternative start codons. The former mechanism is verifiable by enhanced eIF2 phosphorylation and the latter by induction of ATF4 expression [25].

We recently found that BTZ synergizes with the BH3-mimetic ABT-199 to induce apoptosis in various soft tissue sarcoma cells and cell lines. The synergistic activity of ABT-199&BTZ depends on BAX and the MCL-1 antagonist NOXA [26]. In continuation of this work, we show here that ABT-199 transactivates *PMAIP1/*NOXA proposedly via stress-induced ATF4 with concomitant induction of ATF3 and thereby synergizes with clinically relevant PIs to overcome resistance and induce cell death.

## Results

### ABT-199 synergizes with PIs to induce cell death and enhanced expression of NOXA

**Fig. 1:**
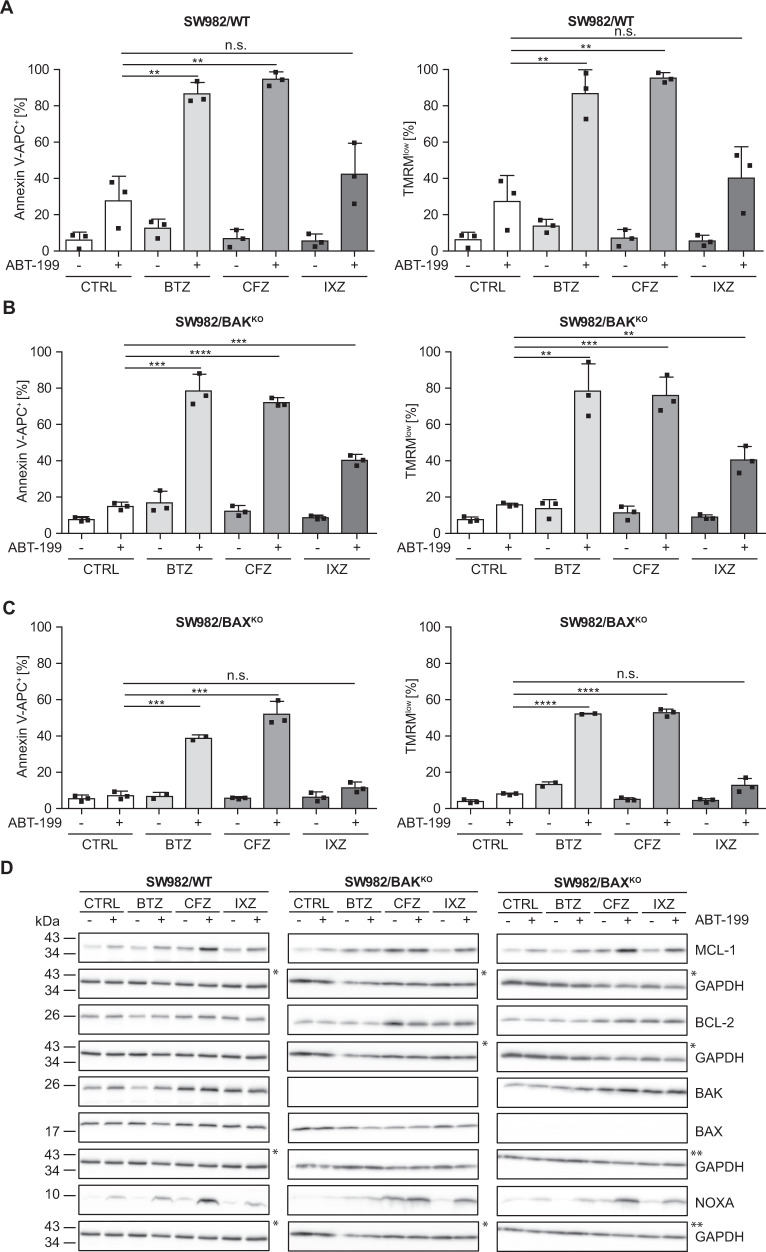
ABT-199&PIs synergize to induce cell death with concomitant induction of NOXA expression. **A**–**C** SW982/WT and corresponding knock-out cell lines SW982/BAK^KO^ and SW982/BAX^KO^ were cultured for 24 h in presence or absence of 15 µM ABT-199 and/or 5 nM PI (BTZ, CFZ or IXZ). Apoptotic cell death was assessed by flow cytometric analysis of phosphatidyl serine exposure (Annexin V-APC) and relative mitochondrial membrane potential (TMRM). Reduced apoptosis in SW982/BAX^KO^ suggests BAX as relevant mediator of synergism, whereas BAK does not appear to play a major role in early (24 h) apoptosis induction. Graphs show mean and individual data points. Statistical significance was analyzed by an unpaired student´s t-test. **D** SW982/WT, SW982/BAK^KO^ and SW982/BAX^KO^ cells were incubated for 8 h in the presence or absence of 15 µM ABT-199 and/or 5 nM PI (BTZ, CFZ, IXZ; + 10 µM Q-VD-OPh). Expression of MCL-1, BCL-2, BAK, BAX, NOXA, and GAPDH (loading control) was analyzed by Western blot. Asterisks indicate identical Western blot due to cutting/reprobing. BTZ bortezomib, CFZ carfilzomib, IXZ ixazomib, CTRL control.

To investigate whether the synergistic cell death induction by ABT-199 and bortezomib [26] is specific to BTZ or class specific, we combined ABT-199 with the clinically relevant PIs carfilzomib (CFZ) and ixazomib (IXZ) and analyzed cell death in SW982/WT, SW982/BAX^KO^ and SW982/BAK^KO^ soft tissue sarcoma cell lines [26]. Cells were incubated with 5 nM BTZ, CFZ and IXZ alone and in combination with ABT-199. Apoptotic cell death was detected by flow cytometry (Annexin V-APC; TMRM). ABT-199 induced exposure of phosphatidyl serine (Annexin V-APC^+^) and loss of mitochondrial membrane potential (low ΔΨ_m_: TMRM^low^) in 20–40% of SW982/WT (Fig. 1A) and ~18% in SW982/BAK^KO^ (Fig. 1B). In contrast, SW982/BAX^KO^ was largely resistant to ABT-199 induced cell death and loss of mitochondrial membrane potential (Fig. 1C). Incubation with PIs alone resulted in <20% Annexin V-APC^+^ (left panels) and ΔΨ_m_^low^ (right panels) cells (Fig. 1A–C). However, PIs (BTZ, CFZ and (less efficiently) IXZ) synergized with ABT-199 to induce apoptosis (Annexin V-APC^+^; TMRM^low^: >=90% for BTZ and CFZ, > = 40% for IXZ). Knock-out of BAK reduced ABT-199 induced cell death, but only marginally affected cell death induction by the combination of ABT-199 with BTZ or CFZ (Annexin V-APC^+^; ΔΨ_m_^low^: >70%) and had no effect on ABT-199&IXZ induced cell death (Fig. 1B). In contrast, BAX^KO^ strongly reduced Annexin V^+^ and TMRM^low^ cells from >80% to ~40% (ABT-199&BTZ), > 90% to ~50% (ABT-199&CFZ) and ~40% to ~10% (ABT-199&IXZ) (Fig. 1C).

Cell death induction by ABT199&PIs in SW982/BAK^KO^ is similar to SW982/WT (Fig. 1A, B) implicating that BAK is not rate limiting for synergistic apoptosis induction by ABT-199&PI (in the presence of BAX). ABT-199 and BTZ, CFZ, or IXZ induced apoptosis is strongly reduced in BAX deficient cells, indicating that it largely depends on BAX. To investigate the molecular basis for synergistic apoptosis induction, we investigated expression of the anti-apoptotic ABT-199 target protein BCL-2 and the short lived anti-apoptotic MCL-1, that is degraded by the proteasome [27]. We also analyzed expression of BAX and BAK and the BH3-only protein NOXA, because NOXA is also degraded by the proteasome. We did not detect drug induced variations in the expression of BCL-2, BAK or BAX (Fig. 1D). Expectedly, PIs tended to induce enhanced expression of NOXA and enhanced expression of MCL-1 (Fig. 1D). Intriguingly, NOXA expression is increased by ABT-199, and increased NOXA expression is even more pronounced in combination with PIs. Results were confirmed in TP53 deficient H1299/WT and H1299/*TP53* lung cancer cells (Suppl. Figure 2H). In conclusion, expression of NOXA correlates with efficacy of cell death induction in the order ABT-199 < ABT-199+IXZ < ABT-199+BTZ < ABT-199+CFZ. Therefore, compound-specific differences between PIs exist.

### Blocking of β_5_ and β_2_ activity correlates with synergistic cell death induction by ABT-199&PIs

**Fig. 2:**
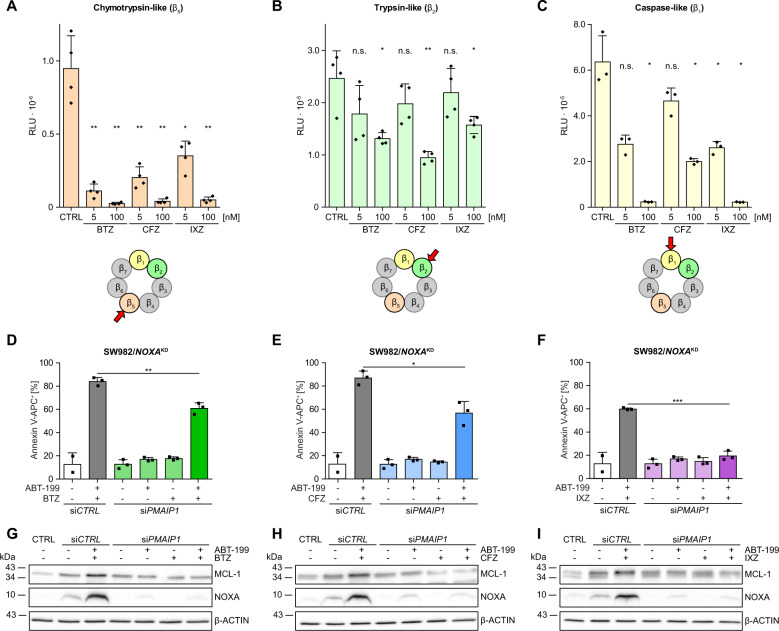
PI specificity modulates NOXA dependent synergistic cell death induction. **A**–**C** SW982/WT cells were pre-incubated for 4 h with 5 nM or 100 nM of BTZ, CFZ or IXZ and Proteasome-Glo^TM^ cell based reagent was added. Luminescence indicating activity of the individual proteasomal subunits was detected after 5 min of incubation. **D**–**F** SW982/WT were transfected with *PMAIP1* siRNA and subsequently incubated for 24 h in the presence or absence of 15 µM ABT-199 and/or 5 nM PI (BTZ, CFZ or IXZ). Apoptotic cell death was assessed flow cytometrically by detecting Annexin V-APC^+^ cells. Graphs represent mean values and individual data points. Statistical significance was calculated by an unpaired student´s *t* test. **G**–**I** After treatment with 15 µM ABT-199 and/or 5 nM PI (BTZ, CFZ, IXZ; + 10 µM Q-VD-OPh) for 8 h each 40 µg total protein per lane were separated on a 12–20% polyacrylamide gradient gel and knock-down of NOXA, with β-ACTIN as loading control, was verified by Western blot. BTZ bortezomib, CFZ carfilzomib, IXZ ixazomib, CTRL control.

The PIs BTZ and IXZ block the β_1_ and β_5_ subunits of the 26 S proteasome, while CFZ blocks β_2_ and β_5_ subunits [24]. To analyze whether protease-specificity of PIs causes the differences in synergistic cell death induction we detected activity of proteasome subunits after incubation with BTZ, CFZ or IXZ. Chymotrypsin-like β_5_-activity is efficiently blocked by BTZ, CFZ, and IXZ (Fig. 2A). CFZ is the most effective β_2_-inhibitor (Fig. 2B) and weakest β_1_-inhibitor (Fig. 2C). Apparently, the combined inhibition of β_5_ and β_2_ plays a role in synergistic cell death induction while β_1_ has minor impact.

### Knock-down of NOXA impairs synergism of ABT-199&PIs

To examine the role of NOXA we knocked down *PMAIP1/*NOXA (Fig. 2D–I) resulting in strongly reduced cell death induction by ABT-199 in combination with BTZ, CFZ or IXZ (Fig. 2D-F; data including all controls in Suppl. Figure 1A-G). *PMAIP1/*NOXA downregulation reduced cell death by ~24% in case of ABT-199&BTZ (Fig. 2D) and 30% in case of ABT-199&CFZ (Fig. 2E) whereas ABT-199&IXZ induced cell death was blocked (<20%; Fig. 2F). Thus, knock-down of NOXA unambiguously impairs apoptosis induction by ABT-199&PIs. However, residual cell death indicates existênce of alternative pathways induced by ABT-199&BTZ or ABT-199&CFZ. We thought to elucidate the mechanisms underlying induction of NOXA. Because induction of *PMAIP1/*NOXA was associated with enhanced expression of MCL-1 whereas in si*PMAIP1/*NOXA transfected cells basal expression of MCL-1 was unchanged (Fig. 2G-I), we excluded the possibility that enhanced MCL-1 expression caused increased NOXA expression. To analyze whether NOXA induction is the consequence of BAX/BAK induced MOMP and apoptosis we incubated HCT116 cells in the presence or absence of ABT-199 and/or BTZ for 24 h. Expectedly, knock-out of BAX and/or BAK reduces synergistic cell death induction (Suppl. Figure 1H) while NOXA expression is invariably induced by ABT-199 (in the presence of Q-VD-OPh) (Suppl. Figure 1I). Thus, induction of NOXA by ABT-199 is independent/upstream of MOMP and apoptosis.

### Negligible TP53-dependent induction of NOXA by PIs

**Fig. 3:**
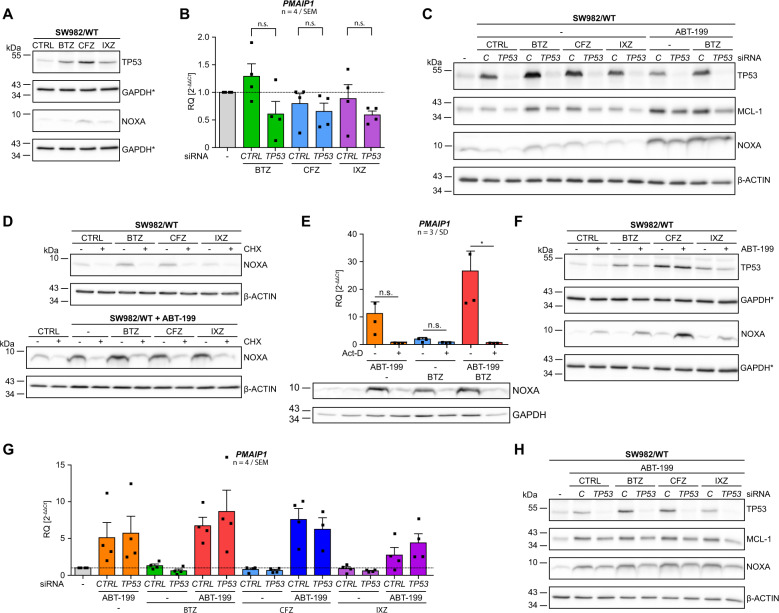
Negligible TP53-dependent induction of NOXA by PIs. **A** Cells were incubated with 5 nM PI (BTZ, CFZ, IXZ; + 10 µM Q-VD-OPh) for 8 h and expression of TP53 and NOXA were analyzed by Western blot, using GAPDH as loading control. **B**, **C** Cells were transfected with si*CTRL* or si*TP53* and subsequently incubated with indicated inhibitors for 8 h. **B** Expression of *PMAIP1* was analyzed by qRT-PCR and **C** Western blot was performed to analyze expression of TP53, MCL-1, NOXA, and β-ACTIN (loading control). **D** Western blot analysis of SW982/WT incubated with PIs alone or in combination with ABT-199 (+ 10 µM Q-VD-OPh) for 8 h were carried out to analyze expression of NOXA (β-ACTIN as loading control) in the absence or presence of 10 µM cycloheximide. **E** qRT-PCR and Western blot analysis of *PMAIP1/*NOXA expression in SW982/WT cells incubated with ABT-199, BTZ or ABT-199&BTZ for 8 h in the absence or presence of 1 µM actinomycin D were performed (GAPDH as loading control). **F** SW982/WT cells were incubated with BTZ, CFZ or IXZ in the absence or presence of ABT-199 for 8 h. Expression of NOXA (GAPDH as loading control) was analyzed by Western blot. **G** SW982/WT were transfected with si*CTRL* or si*TP53* and incubated with the indicated PIs alone or in combination with ABT-199. Subsequently, *PMAIP1* expression was analyzed by qRT-PCR. **H** SW982/WT cells were transfected with si*CTRL* or si*TP53* and incubated with ABT-199 alone or in combination with BTZ, CFZ, or IXZ for 8 h. Cell extracts were analyzed for the expression of TP53, MCL-1 and NOXA by Western blot (β-ACTIN as loading control). Graphs show mean and individual data points. Asterisks indicate identical Western blot due to cutting/reprobing. Act-D actinomycin D, BTZ bortezomib, CFZ carfilzomib, CHX cycloheximide, IXZ ixazomib, CTRL control.

*PMAIP1* is a TP53-inducible gene [18, 28] and NOXA and TP53 are post-transcriptionally regulated by proteasomal degradation [20]. Thus, we investigated whether proteasome inhibition augments expression of NOXA. SW982/WT cells were incubated with PIs and extracts were analyzed for expression of NOXA. However, PIs only very slightly augmented expression of NOXA (Figs. 1D, 3A). Because proteasome inhibition also stabilizes TP53 ([29] Fig. 3A), we investigated whether PI-stabilized TP53 induces NOXA. We transfected SW982/WT with siRNA targeting *TP53* (si*TP53*) and analyzed transcription of *PMAIP1* by qRT-PCR upon inhibition of the proteasome. BTZ, CFZ and IXZ had no significant impact on expression of *PMAIP1* (Fig. 3B). Knock-down of *TP53* reduced *PMAIP1* expression although not statistically significant (Fig. 3B). Western blot analysis showed slightly enhanced NOXA expression (BTZ, CFZ) and knock-down of *TP53* reduced NOXA expression (Fig. 3C). Thus PI mediated stabilization of TP53 has negligible impact on *PMAIP1*/NOXA induction. Notably, ABT-199 and ABT-199&BTZ caused comparably stronger expression of NOXA that was hardly attenuated by si*TP53*. To further delineate induced expression of NOXA from NOXA stabilization by proteasome inhibition we co-applied cycloheximide (CHX) to block protein synthesis. CHX abolishes PI induced NOXA expression (Fig. 3D, upper panel) indicating that enhanced expression of NOXA is based on protein synthesis. Intriguingly, strong expression of NOXA in response to ABT-199 (without or with PIs) is blocked by CHX and thus also relies on protein synthesis (Fig. 3D, lower panel).

To test whether ABT-199 mediates enhanced transcription we analyzed *PMAIP1* mRNA by real-time PCR. Astonishingly, ABT-199 induced 10-fold expression of *PMAIP1/*NOXA that was even further enhanced (>25-fold) in response to ABT-199&BTZ. This enhanced transcription of *PMAIP1/*NOXA was blocked when transcription was blocked by actinomycin D (Act-D) (Fig. 3E, bar graph). Transcriptional regulation was further investigated by analyzing the impact of ABT-199 on expression of transgenic NOXA in SW982 cells. In line with previous data ABT-199 enhanced the expression of endogenous NOXA while expression of transgenic NOXA was unaffected (Suppl. Figure 3A). Noteworthy, these findings demonstrate that ABT-199 alone and in combination with BTZ induces transcription of *PMAIP1/*NOXA that translates into enhanced expression of NOXA (Fig. 3E, lower panel).

We investigated a role of TP53 in enhanced expression of NOXA in response to ABT-199 with or without PIs. As noted, expression of NOXA was enhanced by ABT-199 and unleashed in combination with BTZ or CFZ. In contrast, TP53 was slightly augmented by PIs but unaffected by ABT-199, implying TP53-independent NOXA induction (Fig. 3F). Nevertheless, we knocked down *TP53* and analyzed ABT-199 and/or PI mediated *PMAIP1* gene expression. ABT-199 alone and in combination with BTZ, CFZ and to a lesser extent IXZ induced expression of *PMAIP1* that was not affected by *TP53* knock-down. Furthermore, *PMAIP1* induction by ABT-199 alone and in combination with PIs was significantly stronger than induction by proteasome inhibition (Fig. 3G). Western blot showed that induction of NOXA expression mainly depends on ABT-199 and is augmented by PIs. Thus, qRT-PCR (Fig. 3G) and Western blot (Fig. 3C, H) indicate that ABT-199 strongly induces expression of NOXA by a transcription (Fig. 3E) and translation (Fig. 3D, lower panel) dependent mechanism, independent of TP53.

### TP53 is dispensable for ABT-199&PI mediated induction of NOXA expression and cell death

To broaden the analyzes, we investigated ABT-199&PI induced apoptosis in the rhabdomyosarcoma cell line RD with mutant *TP53* [30], the non-small cell lung cancer cell line H1299 with deleted *TP53* [31], and H1299 with reconstituted expression of TP53. In each cell line, ABT-199&PIs synergistically induced cell death (Suppl. Figure 2A–C: Annexin V-APC; Suppl. Figure 2D–F: TMRM). In both, H1299/WT and H1299/*TP53*, ABT-199 strongly induced *PMAIP1* mRNA expression (Suppl. Figure 2G). Western blot showed that in H1299 NOXA induction was induced by ABT-199 rather than PIs (Suppl. Figure 2H). These data confirm cell type and TP53-independent induction of NOXA by ABT-199 and substantiate synergistic cell death induction by ABT-199&PIs.

### ABT-199 activates ATF3&ATF4 that mediate NOXA expression

Data show that the BCL-2 inhibitor ABT-199 induces expression of the MCL-1 antagonizing NOXA by a TP53-independent transcriptional mechanism. Roca-Portoles et al. [32] postulated that ABT-199 induces mitochondrial metabolic reprogramming, whereby ABT-199 impairs complex I/II activity of the electron transport chain (ETC), resulting in enhanced reductive carboxylation. To analyze metabolic reprogramming we detected Citrate and α-ketoglutarate by mass spectrometry. Analysis evidenced an increased ratio of α-ketoglutarate:citrate after incubation with ABT-199 and ABT-199&BTZ (Suppl. Figure 4A–C) confirming metabolic reprogramming.

**Fig. 4:**
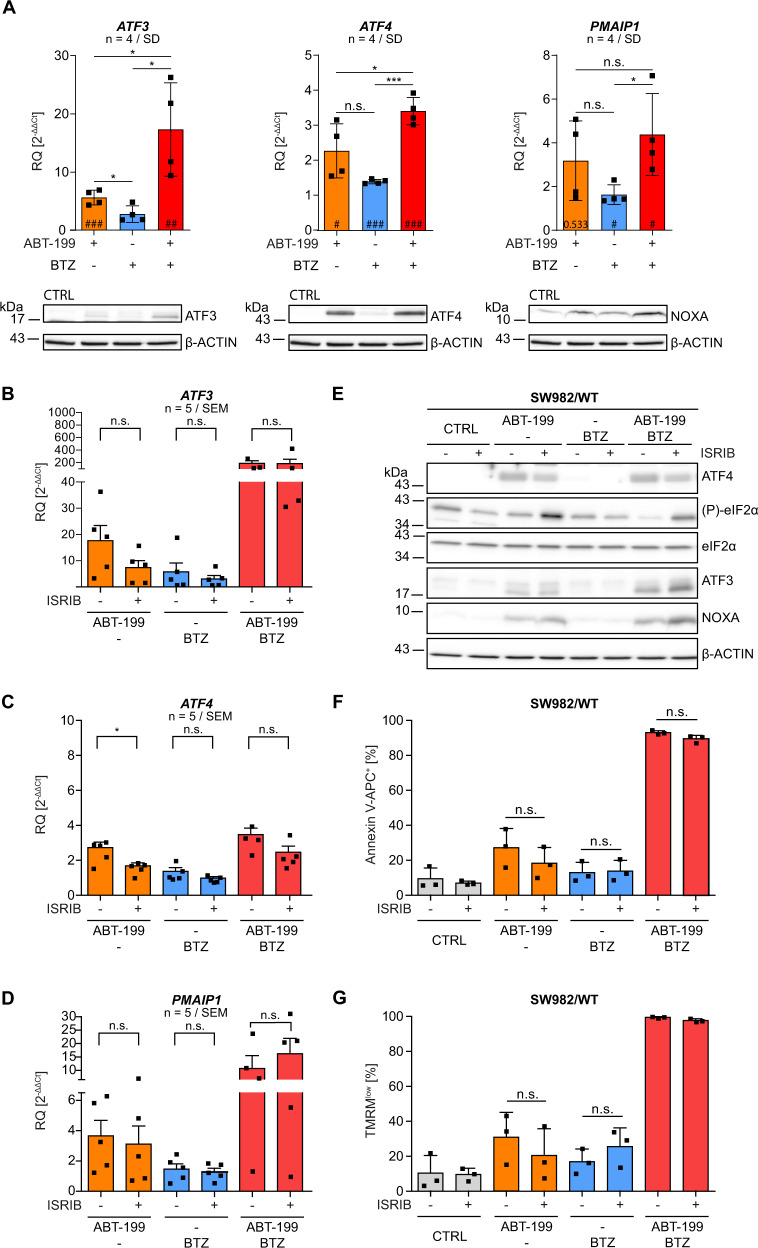
ABT-199 activates ATF3&ATF4 that induce NOXA expression by activation of the integrated stress response pathway. **A** SW982/WT cells were incubated in the presence or absence of 15 µM ABT-199 and/or 5 nM BTZ ( + 10 µM Q-VD-OPh) for 8 h. Expression of *ATF3/*ATF3 (left panel)*, ATF4/*ATF4 (middle panel) or *PMAIP1/*NOXA (right panel) was analyzed by qRT-PCR and Western blot (β-ACTIN as loading control). **B**–**D** SW982/WT cells were incubated in the presence or absence of 15 µM ABT-199 and/or 5 nM BTZ ( + 10 µM Q-VD-OPh) with/without 200 nM ISRIB for 8 h and mRNA expression of *ATF3, ATF4* and *PMAIP1* was analyzed by qRT-PCR. **E** SW982/WT cells were incubated with 15 µM ABT-199 and/or 5 nM BTZ ( + 10 µM Q-VD-OPh) in the presence or absence of 200 nM ISRIB for 8 h. Each 40 µg protein were analyzed by Western blot for the expression of ATF4, (P)-eIF2α, eIF2α, ATF3 and NOXA (β-ACTIN as loading control). **F, G** SW982/WT cells were incubated with 15 µM ABT-199 and/or 5 nM BTZ with/without 200 nM ISRIB for 24 h and apoptosis was assessed flow cytometrically after staining cells with Annexin V-APC and TMRM. Graphs show mean values and individual data points. Statistical significance was analyzed by an unpaired student´s t-test. # indicates statistical power vs. control. BTZ bortezomib, CTRL control, ISRIB integrated stress response inhibitor.

A disbalance in the ETC activates the integrated stress response pathway (ISR) and the activating transcription factor ATF4 that, together with ATF3, can induce expression of *PMAIP1*/NOXA [28]. Thus, we analyzed whether ATF3 and ATF4 mediate NOXA induction in response to ABT-199 by qRT-PCR and Western blot. Proteasome inhibition had negligible impact on ATF3 expression but ABT-199 enhanced transcription (6-fold) and expression (WB) of ATF3 (Fig. 4A, left panel). ATF3 expression was strongly induced by incubation with ABT-199&BTZ (18-fold; WB; Fig. 4A, left panel). ABT-199 alone and in combination with BTZ induced expression of *ATF4* mRNA by 2.3 and 3.4-fold, respectively (Fig. 4A, middle panel), and enhanced expression of ATF4 was indicated in WB analysis (Fig. 4A, middle panel), in line with translational regulation of ATF4. Finally, enhanced expression of ATF3 and ATF4 paralleled enhanced expression of *PMAIP1* mRNA and protein (Fig. 4A, right panel).

### ABT-199 induces activation of the integrated stress response pathway

We next analyzed the role of ISR for NOXA expression and cell death induction. ISR induces phosphorylation of the translation-initiation complex eIF2 subunit α (eIF2α) and (P)-eIF2α inhibits the translation complex eIF2B. This abolishes translation initiation from regular AUG start codons while enhancing translation from alternative AUG-codons resulting in increased expression of the activating transcription factor 4 (ATF4) and it´s downstream target ATF3. To analyze the role of ISR in ABT-199 induced NOXA expression we utilized the specific ISR-**i**nhi**b**itor ISRIB which antagonizes the inhibitory effect of (P)-eIF2α. ISRIB had no significant impact on ABT-199 and BTZ mediated transcriptional induction of *ATF3, ATF4* and *PMAIP1* (Fig. 4B-D). In cells incubated with ABT-199&BTZ, the increased expression of *ATF3*, *PMAIP1*, and *ATF4* was even more pronounced but ISRIB had no significant impact on the expression of *ATF3, ATF4* and *PMAIP1*. Thus, ISRIB showed limited effect on transcriptional induction of ATF3&ATF4 and *PMAIP1*.

ISRIB attenuates stress response and translation of ATF4, thereby reducing the level of active phosphatase complexes, leading to reduced dephosphorylation of (P)-eIF2α and accumulation of (P)-eIF2α (Pakos-Zebrucka 2016). Although Western blot analysis verifies accumulation of (P)-eIF2α in the presence of ISRIB, the expected reduced induction of ATF4 was not detectable (Fig. 4E). Consequently, expression of ATF3 and NOXA is largely unchanged (Fig. 4E). Nevertheless, we investigated whether ISRIB affects cell death induction by ABT 199&BTZ. Since ISRIB did not modulate ATF3, ATF4 and NOXA expression, cell death induction by ABT-199&BTZ was unaffected (Fig. 4F: Annexin V-APC; Fig. 4G: TMRM), which is in line with unchanged NOXA expression (Fig. 4E).

### ATF3&ATF4 double knock-down reduces ABT-199&BTZ mediated NOXA expression and cell death

**Fig. 5:**
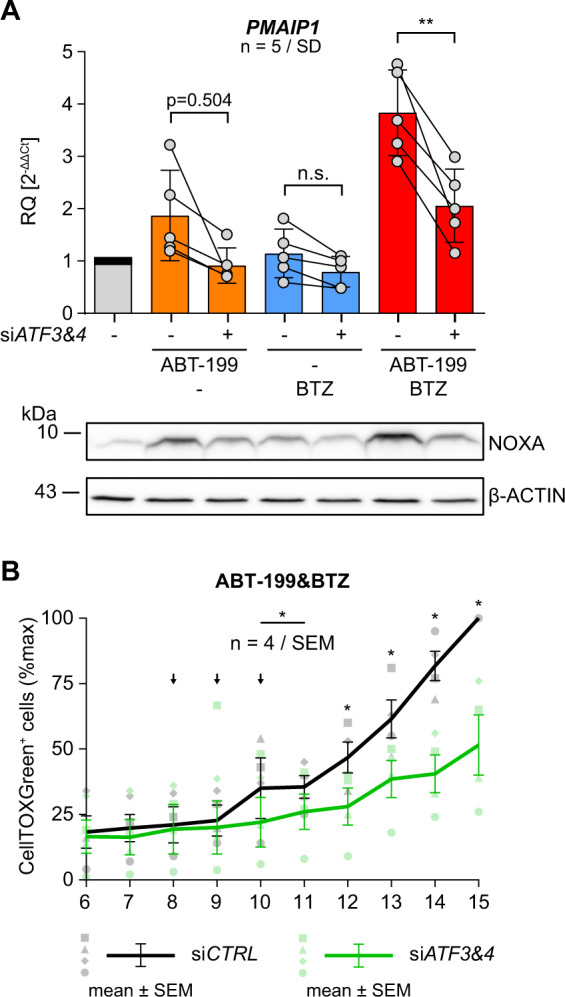
Knock-down of *ATF3&ATF4* reduces ABT-199&PI induced *PMAIP1*/NOXA expression resulting in diminished cell death. **A** SW982/WT cells were transfected with si*CTRL* or si*ATF3*/si*ATF4* and incubated with 15 µM ABT199, 5 nM BTZ or both for 8 h (+ 10 µM Q-VD-OPh). Aliquots from identical samples were analyzed by qRT-PCR and Western blot for expression of *PMAIP1*/NOXA (β-ACTIN as loading control). Graphs show mean values and individual data points. Statistical significance was analyzed by an unpaired student´s *t* test. **B** SW982/WT cells transfected with si*CTRL* or si*ATF3*/si*ATF4* were incubated with 15 µM ABT-199 in combination with 5 nM BTZ. Medium was supplemented with CellTOX Green and cells were monitored by fluorescence microscopy imaging at the indicated time points. Graph shows the number of CellTOX Green positive cells as mean values ± SEM from 4 independent experiments. Statistical significance was calculated by a paired student´s *t* test. Arrows indicate excluded time points 8–10 h (■/□). BTZ bortezomib, CTRL control.

Finally, we directly investigated the relevance of ATF3&ATF4 for ABT-199 mediated NOXA induction. Simultaneous knock-down of *ATF3* and *ATF4* (Suppl. Figure 3B) reduced both *PMAIP1* induction in the presence of ABT-199 alone from 1.8x to 0.9x and significantly reduced induction by ABT-199&BTZ by 50% from 3.8x to 2x (Fig. 5A). Western blot showed that strongest induction of NOXA by ABT-199&BTZ was reduced by knock-down of *ATF3&ATF4*. Thus, it is expected that knock-down of *ATF3&ATF4* also reduces NOXA mediated cell death. Therefore, we investigated cell death in a time kinetic over 15 h using CellTOXGreen^+^. ABT-199&BTZ incubation at time points beyond 12 h resulted in significant fewer CellTOXGreen^+^ cells in *ATF3&ATF4* double knock-down cells as compared to si*CTRL* (Fig. 5B). Thus, ABT-199 induces *ATF3&ATF4* thereby transactivating *PMAIP1/*NOXA and NOXA potentiates ABT-199&BTZ induced apoptosis.

## Discussion

We previously demonstrated that ABT-199 synergizes with BTZ to induce apoptosis in a BAX and NOXA dependent manner. Here, we show that ABT-199 also synergizes with PIs carfilzomib and ixazomib to induce apoptosis. Similar to ABT-199&BTZ, also ABT-199&CFZ enhances expression of NOXA with concomitant cell death induction. ABT-199&IXZ less efficiently induces NOXA and cell death. Reduced efficacy of IXZ may result from the lowest inhibitory activity for the chymotrypsin-like β_5_ activity, but might also result from biological availability or stability. Regardless of PI efficacy, knock-down of *PMAIP1* confirms that NOXA is a key mediator of synergistically induced apoptosis by ABT-199&PIs [26].

Proteasome inhibition stabilizes NOXA and TP53 [20, 21, 29]. Also, NOXA is a TP53-target gene [18] and expression of NOXA in part is regulated by TP53 [33]. However, TP53 (knock-down, mutation, deletion) does not significantly affect ABT-199 induced NOXA expression, indicating TP53 independent regulation (Fig. 3G, H; Suppl. Figure 2). Upregulation of NOXA is mediated by ISR, caused by e.g. erlotinib, 5-azacitidine or fluorizoline [34–36]. Surprisingly, the specific ISR-inhibitor ISRIB [37] had no significant impact on ABT-199 induced *ATF3, ATF4* and *PMAIP1* mRNA expression and could not mitigate ABT-199&BTZ induced expression of *ATF3, ATF4* or *PMAIP1* mRNA. Because ISRIB does not affect ABT-199&BTZ induced *ATF3* and *PMAIP1* expression and even enhances ATF3 and NOXA, ISRIB does not reduce cell death induction by ABT-199&BTZ. Rabouw et al. showed that ISRIB suppresses ISR in a defined window of activation and postulated that ISRIB inhibits ISR only when (P)-eIF2α is relatively low [37].

Wang et al. demonstrated NOXA induction by ISR, already considering ATF3 and ATF4 [38]. This is conceivable since a) ATF3 and ATF4 bind to the CRE (cAMP-responsive) element within the *PMAIP1* promotor and activate NOXA expression [28, 36, 39] and b) ATF3 and ATF4 are crucially involved in ISR. Here, we show that ABT-199 and ABT-199&BTZ activate ATF3, ATF4 and induce ATF3&ATF4 dependent induction of *PMAIP1*/NOXA. Consequently, simultaneous knock-down of *ATF3* and *ATF4* reduces induction of *PMAIP1* expression by ABT-199 and ABT-199&BTZ. In turn, reduced induction of *PMAIP1*/NOXA was functionally associated with a lower number of apoptotic cells, reflecting the importance of NOXA for synergistic cell death induction by ABT-199&BTZ.

Roca-Portoles et al. [32] described a BCL-2 independent effect of ABT-199 [32] that involves the transcription factor ATF4. ABT-199 affects the ETC and the TCA cycle mediating “metabolic reprogramming”, leading to accumulation of α-ketoglutarate and succinate. Concomitantly, the ABT-199 induced imbalance in ETC activates the ISR and ATF4 [32]. In line, activity of the ETC affects sensitivity of MM to ABT-199 induced cell death [40]. Thus, it is conceivable that ABT-199 induced effects (metabolic reprogramming, ISR, and apoptosis) are exacerbated in the combined presence of ABT-199 and BTZ. A likely mechanism is enhanced ATF3&ATF4 mediated expression of *PMAIP1/*NOXA and simultaneously reduced degradation of NOXA by proteasome inhibition. Thus, knock-down of *PMAIP1* reduces ABT-199&BTZ induced cell death.

The transactivation of *PMAIP1* by ABT-199 shown here corroborates a model in which ABT-199 has a double impact on apoptosis regulation by a) directly blocking anti-apoptotic BCL-2 and b) simultaneous inhibition of MCL-1 via transactivation of NOXA. Blocking proteasomal degradation of NOXA augments cell death induction. The ABT-199 induced transactivation of NOXA described here might re-sensitize resistant cancer cells or MCL-1 overexpressing tumors. Importantly, the mechanism described here is independent of TP53 – thus, ABT-199&BTZ is a therapeutic option for TP53-mutant or deleted tumors.

Clinical studies propose that ABT-199 is effective not only in the treatment of AML [41] but also in patients with relapsed chronic lymphocytic leukemia (CLL) [6] or del(17p) CLL [7, 42]. Furthermore, the combination of ABT-199 with BTZ and dexamethasone demonstrated promising efficacy in patients with relapsed/refractory MM in phase 1b and 3 trials [43, 44]. New PIs with different subunit-specificity and pharmacokinetic profiles have been developed and a phase 2 trial investigates ABT-199&CFZ + dexamethasone (NCT02899052), whereas the novel combination ABT-199&IXZ has not achieved clinical trial success to date (NCT03856112). These results might reflect the mechanisms proposed here. In view of the molecular aspects, i.e. BCL-2 inhibition and transactivation of NOXA, we propose that both, expression of BCL-2 and expression of NOXA are relevant for the efficacy of ABT-199&BTZ.

## Conclusion

**Fig. 6:**
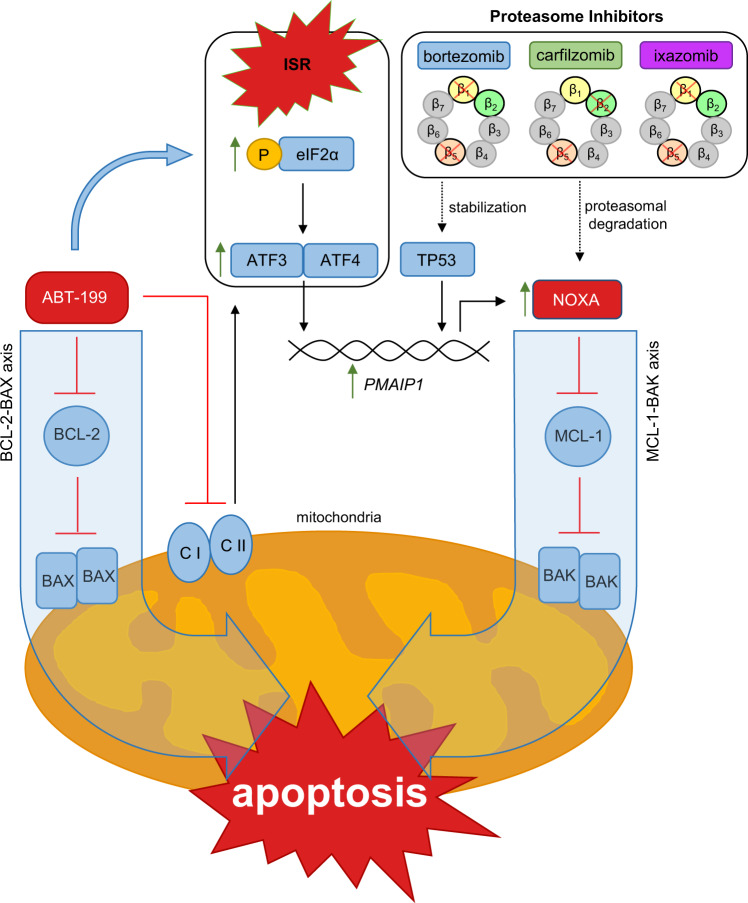
The proposed mechanism underlying synergistic cell death induction by ABT-199&PIs by transactivation of NOXA. Besides stabilization of TP53, proteasome inhibition by BTZ, CFZ, or IXZ prevents proteasomal degradation of the BH3-only protein NOXA. (I) The BH3-mimetic ABT-199 antagonizes the anti-apoptotic activity of BCL-2 thereby releaving BAX. (II) ABT-199 reduces activity of complexes I and II of the mitochondrial ETC mediating metabolic reprogramming that induces ISR manifesting in (P)-eIF2α and increased expression of ATF4 and ATF3. ATF3&ATF4 mediate transactivation of *PMAIP1*/NOXA that inhibits the anti-apoptotic activity of MCL-1. Thus, unrestrained effectors BAX and BAK are free to oligomerize and mediate mitochondrial outer membrane permeabilization (MOMP) that sets the intrinsic apoptosis machinery in motion.

Molecular aspects elucidated in this study are summarized in Fig. 6. We show here for the first time that the BCL-2 inhibitor ABT-199 transactivates NOXA *via* ATF3&ATF4. Thus, in addition to BCL-2 inhibition, transactivated NOXA blocks MCL-1. Therefore, ABT-199 has a double impact on both, the BCL-2–BAX axis and the MCL-1–BAK axis of apoptosis signaling. Additional inhibition of the proteasome reduces NOXA degradation and exacerbates apoptosis induction by ABT-199&PI.

Solid cancers have shown to be less dependent on BCL-2 as compared to leukemias explaining why BH3-mimetics showed little clinical activity in solid tumors [45]. However, intrinsic BH3-mimetic resistance may be overcome by combinatorial treatment with PIs utilizing previously unexpected synergistic mechanisms of action described here. Our data strongly suggest that ABT-199&PI combinations could be active in various solid malignancies.

## Materials & Methods

### Cell culture

Sarcoma cell line RD, SW982 and corresponding knock-out cell lines BAX^KO^, BAK^KO^ and BOK^KO^ [26] were maintained in medium (DMEM; Gibco, Life Technologies, Darmstadt, Germany) supplemented with 10% fetal calf serum (FCS; Biochrom, Germany) and 1% Penicillin/Streptomycin (Gibco, Life Technologies, Darmstadt, Germany). H1299/WT and H1299/*TP53* were maintained in RPMI 1640 (RPMI 1640; Gibco, Life Technologies, Darmstadt, Germany) supplemented with 10% FCS and 1% Penicillin/Streptomycin. HCT116 and corresponding knock-out cell lines BAX^KO^, BAK^KO^ and BAX^KO^/BAK^KO^ were maintained in McCoy´s 5 A (McCoy´s 5 A; Gibco, Life Technologies, Darmstadt, Germany) supplemented with 10% FCS and 1% Penicillin/Streptomycin. Human STS cell line SW982 was authenticated by STR-profiling at the DSMZ. Cells were harvested after incubation in 0.05% trypsin/EDTA solution, centrifuged at 800 x g for 5 min and further processed for subsequent analysis.

### Flow cytometry

*Annexin V-APC, TMRM staining:* Apoptotic cell death was assessed as previously described [46]. Cells were harvested using trypsin/EDTA, resuspended in supernatant and washed in ice-cold PBS. Then, cell pellets were resuspended in 300 μL Annexin V-APC-binding buffer (PBS, 2.5 mM CaCl_2_) supplemented with recombinant chicken Annexin V-APC (ImmunoTools, Friesoythe, Germany) and incubated for 10 min on ice. Subsequently, samples were analyzed using a FACS Lyric flow cytometer (Becton Dickinson, Heidelberg, Germany). To detect loss of mitochondrial membrane potential, cell pellets were resuspended in PBS supplemented with 2% FCS and 50 nM tetramethyl rhodamine (TMRM) (Merck, Darmstadt, Germany) of the potentiometric dye. Cells were incubated at 37 °C for 20 min and fluorescence was analyzed using a FACS Lyric flow cytometer. The proportion of TMRM^low^ and Annexin V-APC^+^ cells was calculated using FACS Suite software.

### Antibodies and reagents

Antibodies used were: anti-ATF3 (Santa Cruz, #sc-188), anti-ATF4 (Cell Signaling, #11815), anti-BAX (Cell Signaling, #2772), anti-BAK (Cell Signaling, #3814), anti-BCL-2 (Cell Signaling, #15071), anti-MCL-1 (Cell Signaling, #5453), anti-NOXA (Merck, #OP180), anti-TP53 (Santa Cruz, #sc-126), anti-eIF2α (Cell Signaling, #9722), anti-(P)-eIF2α (Cell Signaling, #9721), anti-GAPDH (Cell Signaling, #2118), anti-β-ACTIN (Merck, #A5541). Secondary anti-mouse (#7076 S) and anti-rabbit (#7074 S) horseradish peroxidase-coupled antibodies were from Cell Signaling.

### Western blot

Protein expression was analyzed by Western blot as described elsewhere [26]. Cells were harvested by scraping and washed in ice-cold PBS. Whole cell-lysates were prepared in lysis buffer (50 mM Tris-HCl pH 7.6, 250 mM NaCl, 0.1% Triton X-100, 5 mM EDTA; 150 μL/10cm ^2^) supplemented with protease and phosphatase inhibitor cocktails (complete and PhosphoSTOP, Roche, Basel, Switzerland). Samples were sonified (Diagenode, Liège, Belgium) and cleared by centrifugation (15 min, 14000 x g, 4 °C). Protein content was assessed using the Pierce BCA Protein Assay Kit, according to the manufacturer´s protocol (Thermo Fisher Scientific, Waltham, US). Samples were mixed with denaturing sample buffer (1 M Tris-HCl pH 6.8, glycerol, β-mercaptoethanol, 20% sodium dodecyl sulfate (SDS), 1% bromophenol blue) and heated for 5 min at 95 °C. Then equal amounts of protein (typically 40 μg) were separated by SDS-PAGE and blotted (Biometra FastblotTM, Analytic Jena, Jena, Germany) onto nitrocellulose membrane (0.1 μm; GE Healthcare, Munich, Germany) by semi-dry blotting (1 mA/cm ^2^, 1 h). Primary antibody was applied in 5% BSA (Carl Roth, Karlsruhe, Germany) or skim milk powder (Sigma Aldrich, Hamburg, Germany) in PBS-T (PBS, 0.1% Tween-20) over-night at 4 °C. Membranes were washed thrice for 10 min in PBS-T and subsequently incubated with horseradish peroxidase coupled 2nd antibody in 5% skim milk powder in PBS-T (1:2000) for 2 h at room temperature. After washing thrice, ECL solution was applied (SuperSigna West Dura, Thermo Fisher Scientific) and specific bands were detected using a Stella gel documentation system (Raytest Isotopenmessgeräte GmbH, Straubenhardt, Germany).

### RNA interference

Knock-down experiments were performed according to the manufacturer´s protocol. Briefly, 0.6 × 10^5^ cells/12-well or 2.5 × 10^5^ cells/6-well were seeded 24 h prior to transfection. Then, cells were transfected with either 100 µl or 200 µl of Opti-MEM (Gibco) containing 50 nM ON-TARGET Plus Smartpool siRNAs targeting *PMAIP1, or ATF3/ATF4* or non-targeted (NT) (Horizon Discovery, Waterbeach, UK) using Dharmafect Ι reagent (Thermo Fisher Scientific) according to the manufacturer’s protocol. After 24 h cells were incubated with ABT-199 and/or BTZ for additional 24 h. Then, cells were harvested and analyzed by flow cytometry. For verification of knock-down efficacy, cells were lysed and assessed by Western blot analysis.

### Cytotoxicity assay

0.6 × 10^5^ cells were seeded in 12 well plates 24 h prior to transfection. Then, cells were transfected with siRNA specific for *ATF3* and *ATF4* or si*CTRL*. 8 h post transfection, non cell-permeable CellTOX Green Dye (Promega GmbH, Walldorf, Germany) was added and fluorescence was monitored in a Cytation 1 Cell Imaging Multi-Mode Reader (BioTek, Bad Friedrichshall, Germany). Cell death was assessed at indicated time points by counting the number of CellTOX Green positive cells.

### Quantitative RT-PCR (qRT-PCR)

Total RNA from cells was extracted using RNeasy Kit (Qiagen, Valencia, USA). 500 ng of RNA was reverse-transcribed by Moloney Murine Leukemia Virus Reverse Transcriptase (M-MLV RT, Promega GmbH, Walldorf, Germany). For gene expression analysis following Taqman Gene Expression Assay primer/probes were used: Hs00231069 for *ATF3*, Hs00909569 for *ATF4*, Hs00560402 for *PMAIP1*, Hs01034249 for *TP53*, Hs03023943 for *β-ACTIN*, Hs02758991 for *GAPDH*, Hs00362387 for *TUBA1A* (Life Technologies, Darmstadt, Germany). Gene expression analysis was performed in real-time PCR system (7900HT Real-time PCR system, Applied Biosystems, Singapore) and relative quantification was calculated with SDS2.4 software based on the expression level of either *β-ACTIN* alone or the three references genes *β-ACTIN, GADPH* and *TUBA1A*.

### Proteasome-Glo™ Chymotrypsin-Like, Trypsin-Like and Caspase-Like Cell-Based Assays

5 × 10^3^ cells were seeded in 96 well plates 24 h prior to incubation with 5 nM or 100 nM PI (BTZ, CFZ or IXZ) for 4 h. Then, Proteasome-Glo^TM^ chymotrypsin-like, trypsin-like and caspase-like cell based reagent was added accordingly (Promega GmbH, Walldorf, Germany). Each reagent contains a luminogenic protease substrate specific for the three different subunits of the 26 S proteasome (chymotrypsin-like assay for the β_5_ subunit, trypsin-like assay for the β_2_ subunit and caspase-like assay for the β_1_ subunit). Luminescence was detected in an Enspire Multimode Plate Reader (Perkin Elmer, Waltham, Massachusetts, USA). Reduction of luminescence indicates efficacy of the respective PI to inhibit the specific subunits of the 26 S proteasome.

### Statistical analysis

Continuous variables are presented as mean as indicated and categorical variables are given by number and percentages. The statistical significance of differences was analyzed using students t-test. All statistical tests were considered significant when *p* < 0.05. Statistical analyses were calculated using GraphPad Prism (v5.04).

## Supplementary information

Supplemental Figures 1–4

Supplemental Materials & Methods

Supplemental Figure 1

Supplemental Figure 2

Supplemental Figure 3

Supplemental Figure 4

Related Manuscript File Author Contribution

original western blots

## Data Availability

The data generated or analyzed during this study are included in this published article and its supplementary information files. Original data is available from the corresponding author on reasonable request.
