# Supplementary material for: The BCL-2 inhibitor ABT-199/venetoclax synergizes with proteasome inhibition via transactivation of the MCL-1 antagonist NOXA
Source: Cell Death Discov. 2022 Apr 20;8:215. doi: 10.1038/s41420-022-01009-1 (PMC9021261; doi:10.1038/s41420-022-01009-1)
Supplement: Supplementary file 1 — Supplemental Figures 1–4 [file 41420_2022_1009_MOESM1_ESM.docx]

**
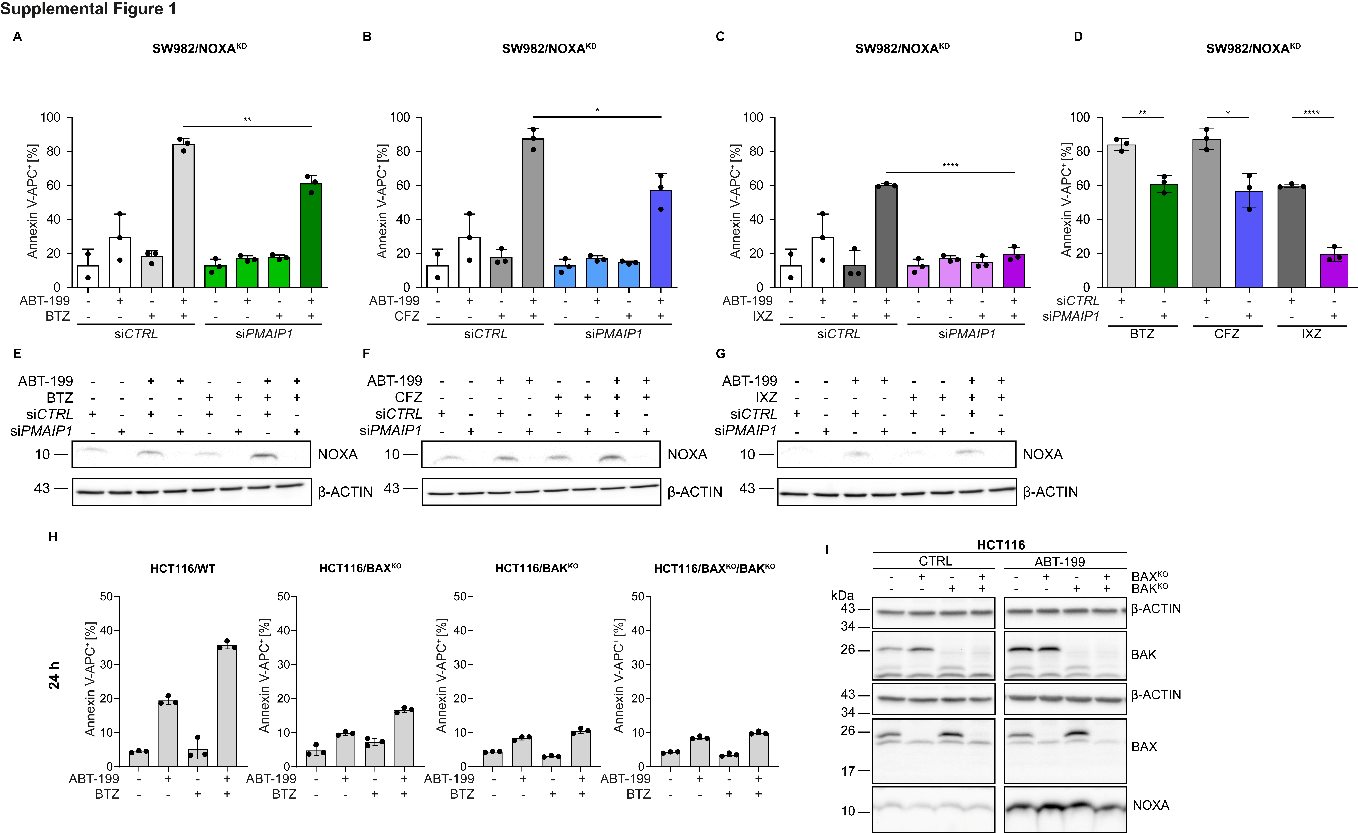
**

**Suppl. Fig. 1: ABT-199&PIs synergistic cell death induction is reduced by Knock-Down of NOXA (extended Fig 2A-G) and independent of MOMP. (A-D)** SW982/WT transfected with siRNA (24 h) were incubated for 24 h in the presence or absence of 15 µM ABT-199 and/or 5 nM PI (**A**: BTZ, **B**: CFZ, **C**: IXZ). **(D)** Graph showing data for ABT-199&BTZ from A-C. Apoptotic cell death was assessed flow cytometrically by detecting Annexin V-APC^+^ cells. Graphs represent mean values and individual data points. Statistical significance was calculated by an unpaired student´s t-test. **(E-G)** siRNA mediated knock-down of NOXA was verified by Western blot (β-ACTIN as loading control). **(H)** Flow cytometric analysis of ABT-199 and/or BTZ induced cell death in HCT116 cell lines shows diminished apoptosis in the absence of BAX and/or BAK. Graphs show mean values and individual data points. **(I)** ABT-199 induces expression of NOXA in HCT116/WT, BAX^KO^, BAK^KO^, and BAX^KO^/BAK^KO^ after 8 h in the presence of Q-VD-OPh. BTZ: bortezomib, CFZ: carfilzomib, CTRL: control, IXZ: ixazomib.

**
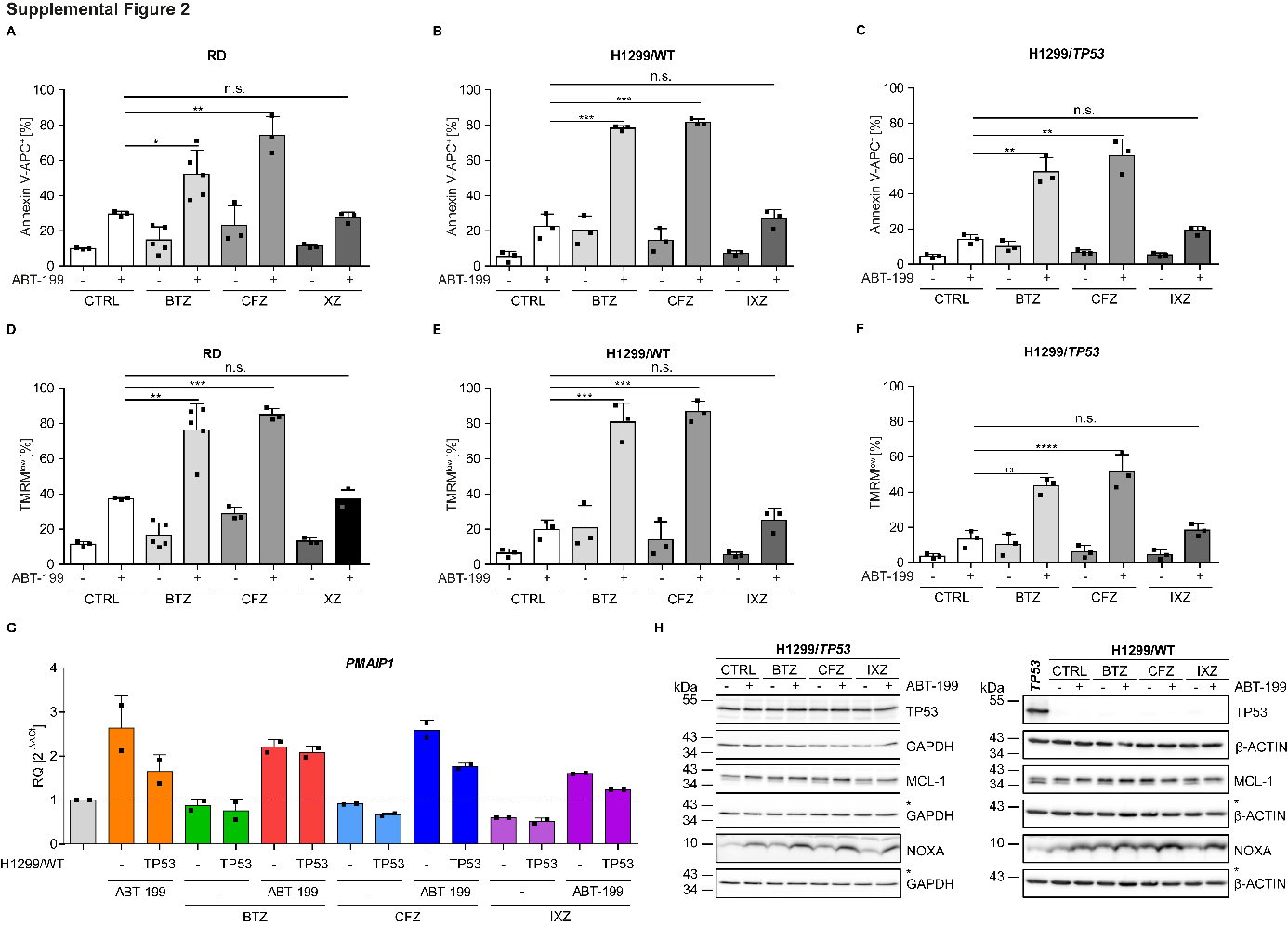
**

**Suppl. Fig. 2: ABT-199&PIs induce expression of NOXA and cell death independent of cell type and TP53. A, D,** RD cells, **B, E** TP53 deficient H1299 cells, and **C, F** TP53 re-expressing H1299 cells were incubated in the presence or absence of 15 µM ABT-199 and/or 5 nM PI (BTZ, CFZ or IXZ) for 24 h. Apoptotic cell death was flow cytometrically detected by staining cells with (**A-C**) Annexin V-APC and (**D-F)** TMRM. Graphs show mean values and individual data points. Statistical significance was calculated using a paired student´s t-test. **G, H** H1299/WT and H1299/*TP53* cells were cultured with or without 15 µM ABT‑199 and/or 5 nM PI (BTZ, CFZ or IXZ; + 10 µM Q-VD-OPh) for 8 h. Cells were harvested and samples were split for RNA and protein extraction. **G** Relative expression of *PMAIP1* mRNA was assessed by qRT-PCR. **H** Western blot analysis of extracts from H1299/WT and H1299/*TP53* verifies absence and presence of TP53, respectively. Also, expression of MCL-1 and NOXA was analyzed (left panel: GAPDH as loading control; right panel: β-ACTIN as loading control). BTZ: bortezomib, CFZ: carfilzomib, CTRL: control, IXZ: ixazomib.

**

**

**Suppl. Fig. 3: Western blot analysis show transgenic expression of NOXA is unaffected by ABT-199 and verifies efficient knock-down of *ATF3* and *ATF4*. (A)** SW982/WT cells were transfected with a vector for the expression of NOXA for 12 h and subsequently incubated with ABT-199 in the presence of Q-VD-OPh for 8 h. Western blot analysis shows induction of endogenously expressed NOXA while expression of exogenous NOXA is unaffected. **(B)** SW982/WT cells were transfected with si*CTRL* or si*ATF3/ATF4* and incubated with 15 µM ABT-199 and/or 5 nM BTZ (+ 10 µM Q-VD-OPh) for 8 h. Each 40 µg total protein per lane were separated on a 12-20% polyacrylamide gradient gel and expression of ATF3 and ATF4 was analyzed by Western blot (β-ACTIN as loading control). BTZ: bortezomib, CTRL: control.





**Suppl. Fig. 4: ABT-199 shifts TCA metabolism towards increased reductive carboxylation.** SW982/WT cells were cultured in absence or presence of 15 µM ABT-199 and 5 nM BTZ (+ 10 µM Q-VD-OPh) for 8 h. Cells were harvested by scraping in ice-cold 80% MeOH and **A, B** cell extracts were analyzed for α-ketoglutarate and citrate content by mass spectrometry. **C** ABT-199 mediated metabolic reprogramming of cellular metabolism manifests in increased α-ketoglutarate / citrate ratio as compared to CTRL. Graphs show mean values and individual data points. Statistical significance was calculated using a paired student´s t-test. BTZ: bortezomib, CTRL: control.
