# Supplementary material for: The BCL-2 inhibitor ABT-199/venetoclax synergizes with proteasome inhibition via transactivation of the MCL-1 antagonist NOXA
Source: Cell Death Discov. 2022 Apr 20;8:215. doi: 10.1038/s41420-022-01009-1 (PMC9021261; doi:10.1038/s41420-022-01009-1)
Supplement: Supplementary file 2 — Supplemental Materials & Methods [file 41420_2022_1009_MOESM2_ESM.docx]

**Supplemental Materials & Methods**

**Analysis of transgenic expression of NOXA by vector transfection**

3,5 x 10^5^ cells were seeded in 6 well plates 24 h prior to transfection. For analysis of transgenic expression of NOXA, pHV Ad2 TRE-Puro myc NOXA vector was generated as published for Nbk/BIK earlier [1]. Then, 2 µg pHV Ad2 TRE-Puro myc NOXA vector and 0,5 µg pTET-OFF vector were cotransfected using PEI (Polysciences Europe GmbH, Hirschberg an der Bergstrasse, DE) transfection reagent at a ratio of 1:6 (vector DNA:PEI) in serum- and antibiotic-free medium. After 6 h, FCS was added and incubated for another 12 h. At the next day, cells were incubated with 15 µM ABT (+ 10 µM Q-VD-OPh) for 8 h and then harvested as described in method Western blot. The protein expression of endogenously expressed NOXA versus expression of exogenous NOXA was analyzed via Western blot.

**TCA-metabolite analysis by mass spectrometry**

3,5 x 10^5^ cells were seeded in 6 well plates 24 h prior to incubation with 1 µM or 15 µM ABT and/or 5 nM BTZ (+ 10 µM Q-VD-OPh) for 8 h. Cells were harvested in cold PBS by scraping and counted. Then, pellets were incubated in 80% MeOH on dry ice for 15 min. Samples were centrifuged for 5 min at 4 °C at 14000 x g and supernatant (extract) was transferred to a fresh tube. Samples were spiked with standard metabolites, cell extracts were separated via gas chromatography (7890A System, Agilent, Santa Clara, USA) and then α-ketoglutarate and citrate were determined by GC–MS (MSD 5975C System, Agilent, Santa Clara, USA) analysis as described previously [2].

**Supplemental Bibliography**

1. Gillissen B, Essmann F, Graupner V, Starck L, Radetzki S, Dorken B, et al. Induction of cell death by the BH3-only Bcl-2 homolog Nbk/Bik is mediated by an entirely Bax-dependent mitochondrial pathway. EMBO J; 2003;22:3580-90.

2. Hofmann U, Maier K, Niebel A, Vacun G, Reuss M, Mauch K. Identification of metabolic fluxes in hepatic cells from transient 13C-labeling experiments: Part I. Experimental observations. Biotechnol Bioeng; 2008;100:344-54.
