# Supplementary figures and images for: The BCL-2 inhibitor ABT-199/venetoclax synergizes with proteasome inhibition via transactivation of the MCL-1 antagonist NOXA
Source: Cell Death Discov. 2022 Apr 20;8:215. doi: 10.1038/s41420-022-01009-1 (PMC9021261; doi:10.1038/s41420-022-01009-1)

Supplemental Figure 2

A

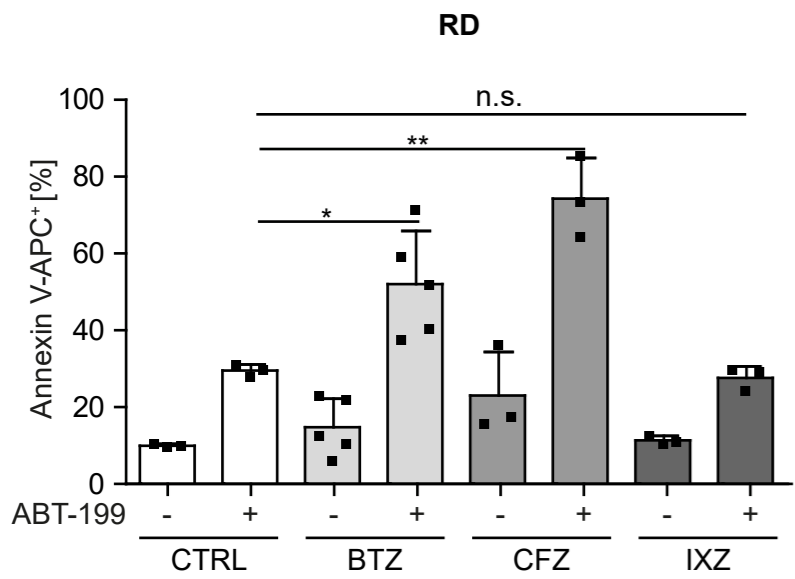

B

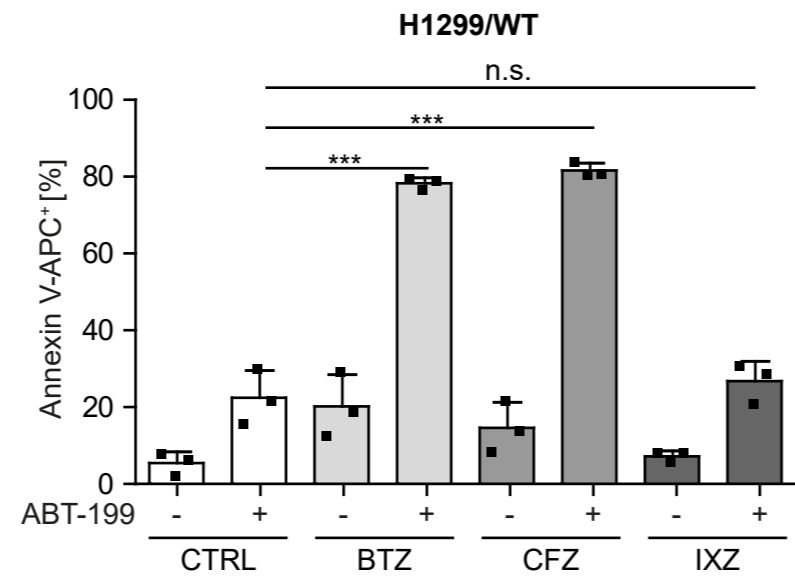

C

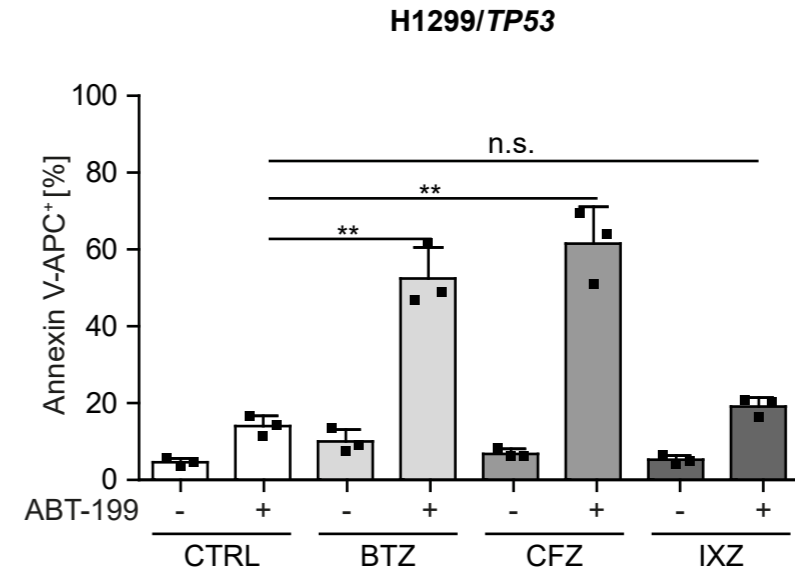

D

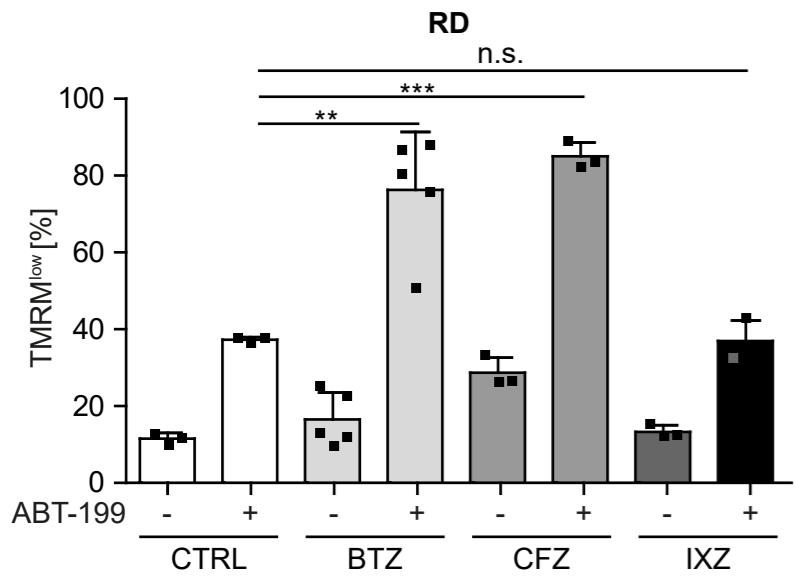

E

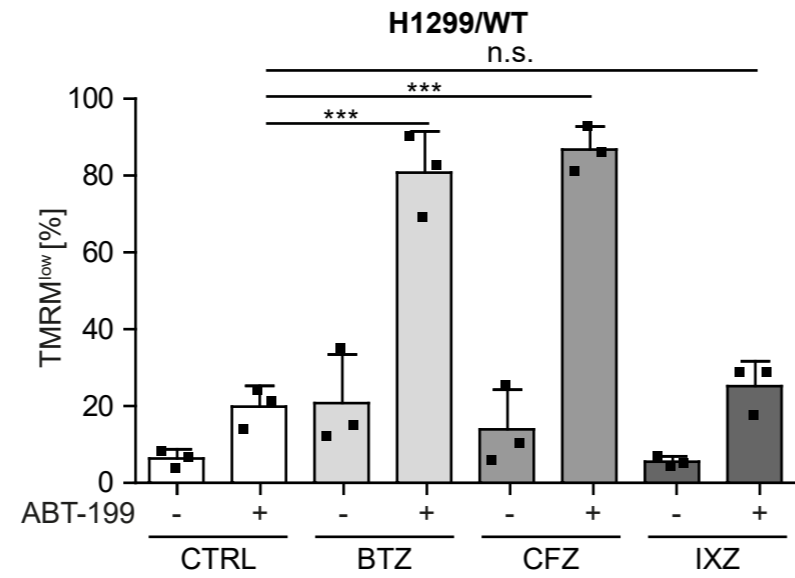

F

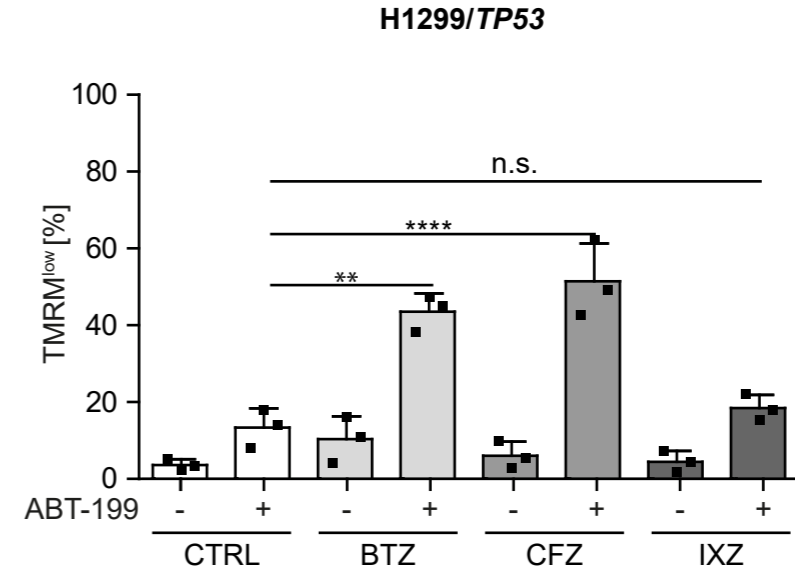

G

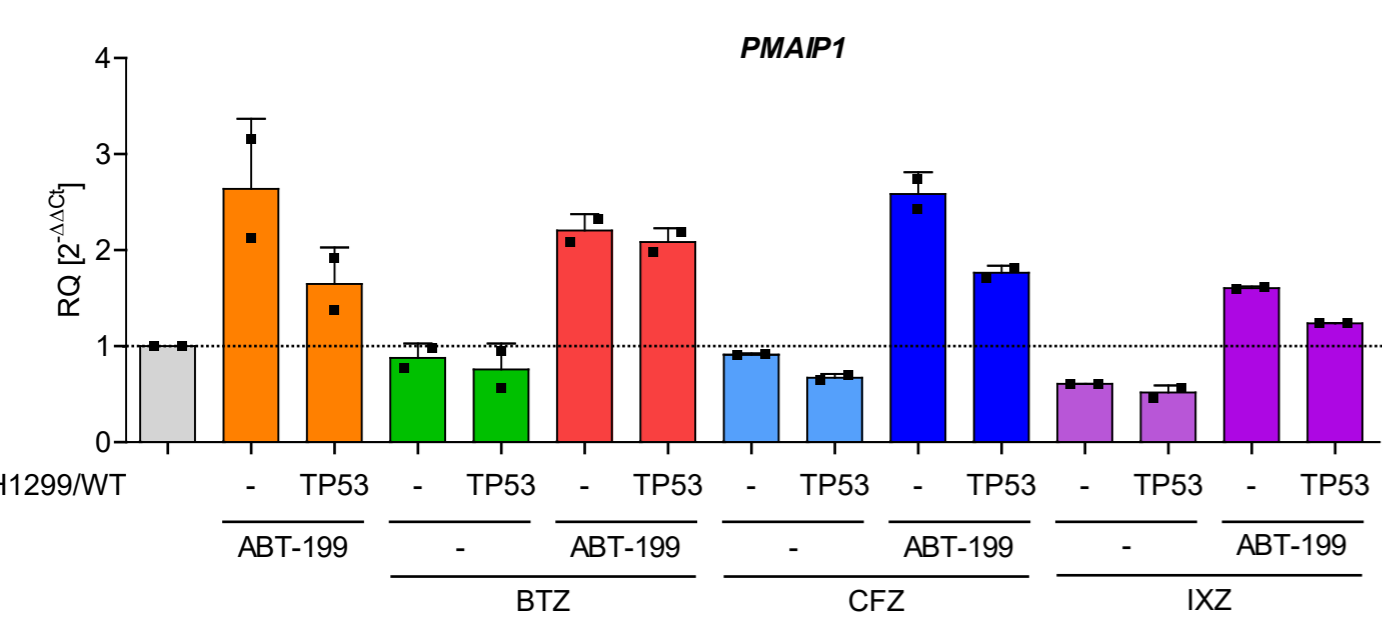

H

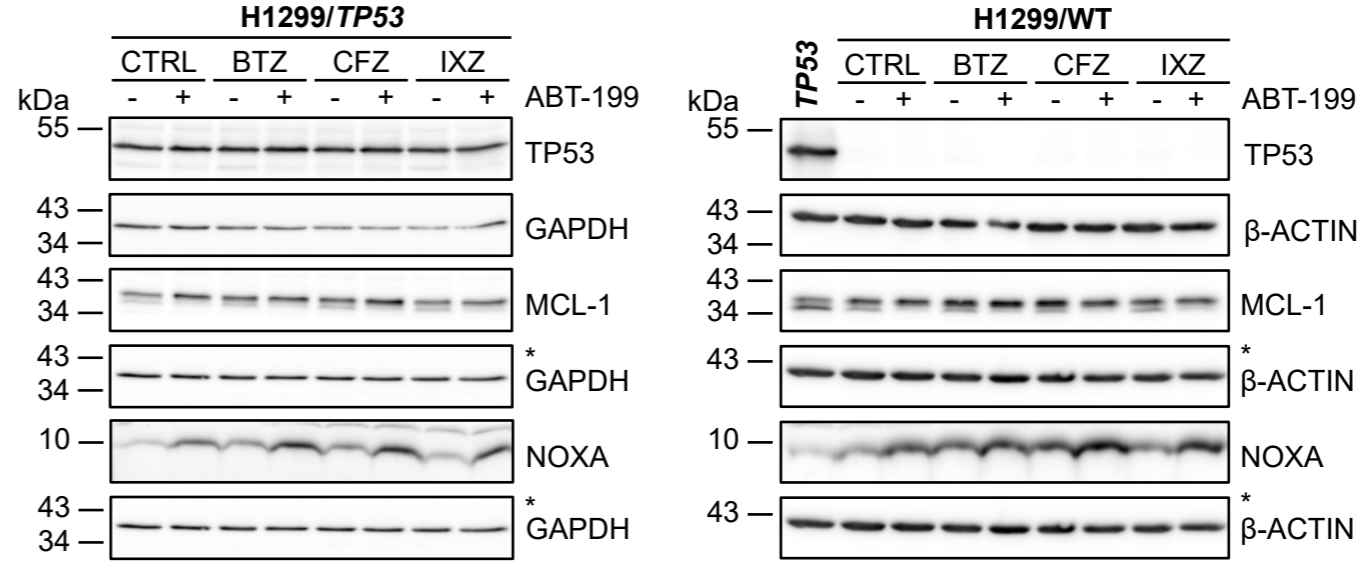

Supplement: Supplementary file 4 — Supplemental Figure 2 [file 41420_2022_1009_MOESM4_ESM.pdf]

# Supplemental Figure 3

**A**

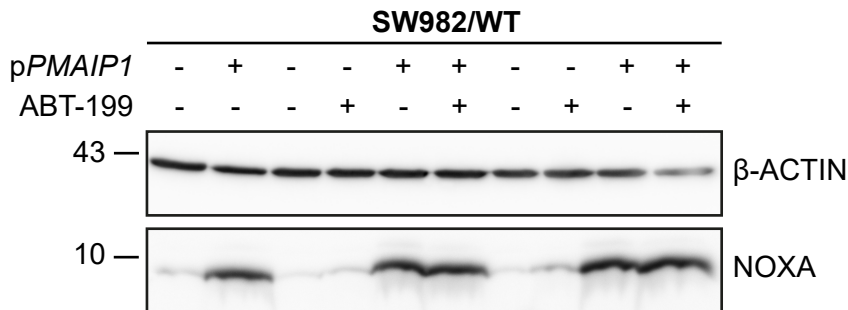

**B**

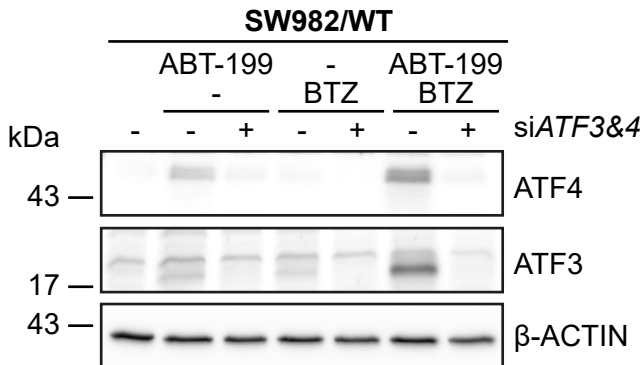

Supplement: Supplementary file 5 — Supplemental Figure 3 [file 41420_2022_1009_MOESM5_ESM.pdf]

**Supplemental Figure 4**

**A**

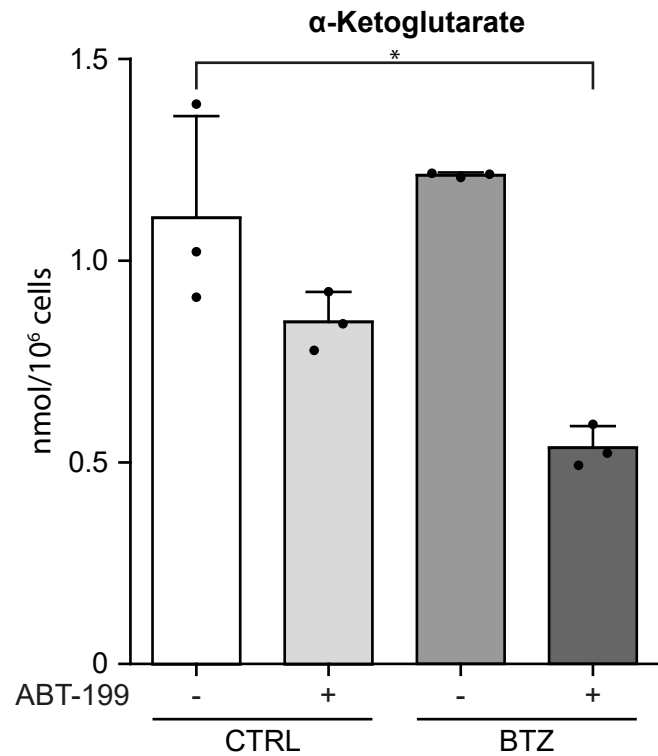

**B**

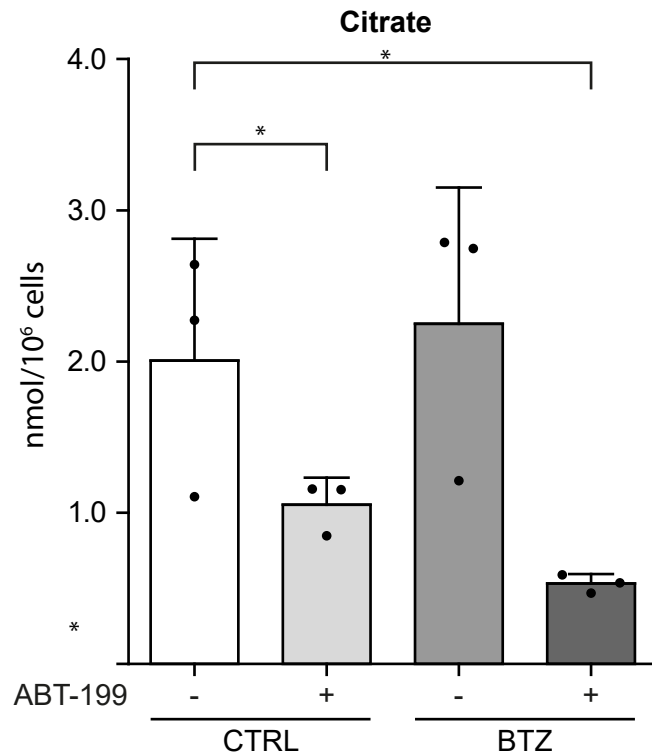

**C**

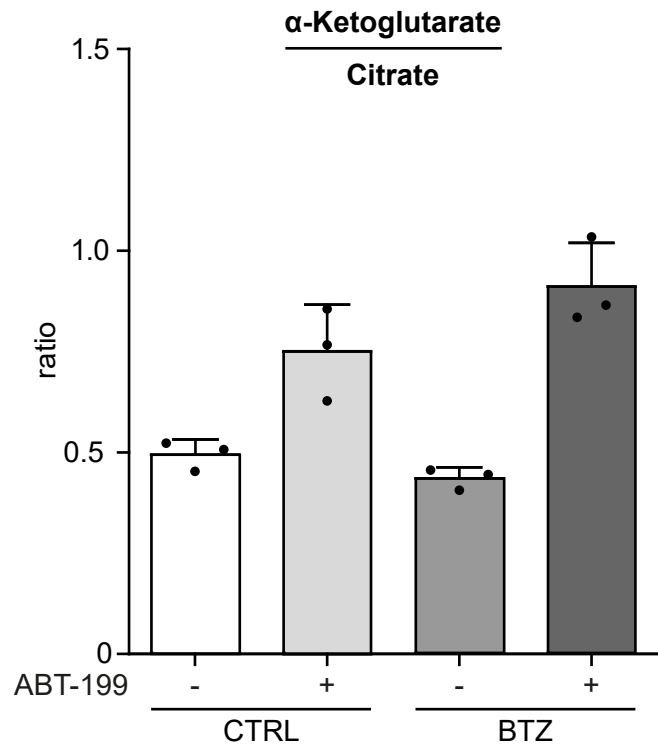

Supplement: Supplementary file 6 — Supplemental Figure 4 [file 41420_2022_1009_MOESM6_ESM.pdf]
