# Supplementary material for: The BCL-2 inhibitor ABT-199/venetoclax synergizes with proteasome inhibition via transactivation of the MCL-1 antagonist NOXA
Source: Cell Death Discov. 2022 Apr 20;8:215. doi: 10.1038/s41420-022-01009-1 (PMC9021261; doi:10.1038/s41420-022-01009-1)
Supplement: Supplementary file 7 — Related Manuscript File Author Contribution [file 41420_2022_1009_MOESM7_ESM.pdf]

DECLARATION OF CONTRIBUTIONS TO ARTICLE

ADMC

Manuscript Number: CDD-21-1831

Journal Name: *Cell Death & Differentiation* (the 'Journal')

Proposed Title of the Contribution:

The BCL-2 Inhibitor ABT-199/Venetoclax Synergizes With Proteasome Inhibition Via Transactivation Of The MCL-1 Antagonist NOXA (the 'Contribution)

Author(s):

Sandra Weller, Astrid Toennießen, Benjamin Schaefer, Tobias Beigl, Alina Muenchow, Kathrin Böpple, Ute Hofmann, Bernhard F. Gillissen, Walter E. Aulitzky, Hans-Georg Kopp, Frank Essmann (the 'Authors')

For all *CDD* articles, each person named as an author in the published version must be able to show he or she has contributed substantially to the article.

Authorship credit should be based on 1) substantial contributions to conception and design, acquisition of data, or analysis and interpretation of data; 2) drafting the article or revising it critically for important intellectual content; and 3) final approval of the version to be published. Authors should meet conditions 1, 2 and 3.

Any person who cannot be shown to have made a substantial contribution to the article cannot be listed as an author in the final version. The name of any person who is deemed to have made a minor contribution can, however, appear in the Acknowledgments section of the article.

Please complete the table below to indicate the contributions of all named authors to the manuscript.

Author Full Name:	Specification of Contribution to the Manuscript:
Sandra Weller	contributed to concept and design of study, performed experiments, wrote manuscript, prepared figures, acquired and interpreted data, reviewed the manuscript
Astrid Toennießen	performed experiments, acquired and interpreted data, corrected the manuscript
Benjamin Schaefer	contributed to initiation of experimental design, data acquisition and interpretation, corrected the manuscript
Tobias Beigl	contributed to initiation of experimental design, data acquisition and interpretation, corrected the manuscript
Alina Muenchow	contributed critically to initiation of the study, reviewed of manuscript and data, corrected the manuscript
Kathrin Böpple	contributed critically to the study, experimental design, data acquisition and interpretation, corrected the manuscript
Ute Hofmann	experiments, data acquisition & interpretation, corrected the manuscript
Bernhard F. Gillissen	contributed to concept and design of study, experiments, data analysis, manuscript writing, figure preparation, manuscript revision
Walter E. Aulitzky	contributed to initiation of experiments & methods, reviewed the data, results and manuscript
Hans-Georg Kopp	conceptualized and designed the study and experiments, contributed to data analysis, wrote the manuscript, reviewed data, figures and manuscript
Frank Essmann	conceptualized and designed the study, initiated methods and experiments, supervised experiments, analyzed and interpreted data, wrote the manuscript, designed and repared figures
CDD-21-1831	CDD-21-1831
CDD-21-1831	CDD-21-1831

Please complete the table below to indicate the contributions of all named authors to the figures.

Figure 1:

Sandra Weller, Alina Muenchow,

Figure 2:

Sandra Weller, Tobias Beigl

Figure 3:

Sandra Weller, Benjamin Schaefer

Figure 4:

Sandra Weller, Kathrin Böpple, Benjamin Schaefer

Figure 5:

Sandra Weller, Benjamin Schaefer

Figure 6:

Sandra Weller
Supplemental Figure 1: Astrid Toennießen
Supplemental Figure 2: Sandra Weller
Supplemental Figure 3: Ute Hofmann

Signed for and on behalf of the Author(s):	Print Name:	Date:
[signature]	Frank Essmann	August 18th, 2021
