# Supplementary material for: The BCL-2 inhibitor ABT-199/venetoclax synergizes with proteasome inhibition via transactivation of the MCL-1 antagonist NOXA
Source: Cell Death Discov. 2022 Apr 20;8:215. doi: 10.1038/s41420-022-01009-1 (PMC9021261; doi:10.1038/s41420-022-01009-1)

Supplemental Figure 1

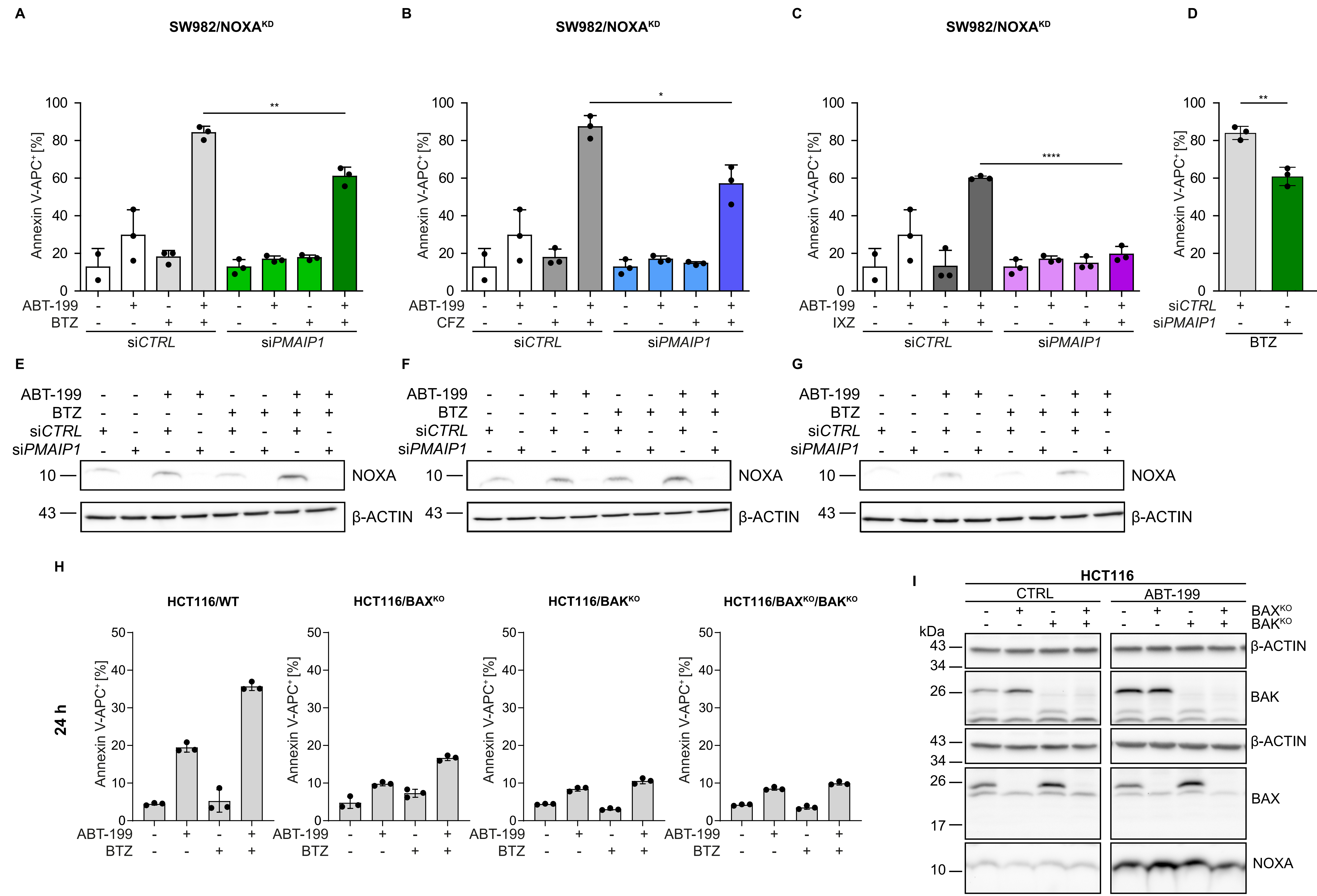

Supplement: Supplementary file 3 — Supplemental Figure 1 [file 41420_2022_1009_MOESM3_ESM.pdf]
