# Supplementary material for: The BCL-2 inhibitor ABT-199/venetoclax synergizes with proteasome inhibition via transactivation of the MCL-1 antagonist NOXA
Source: Cell Death Discov. 2022 Apr 20;8:215. doi: 10.1038/s41420-022-01009-1 (PMC9021261; doi:10.1038/s41420-022-01009-1)

Originalbilder Manuskript

Fig. 1 D

SW982/WT

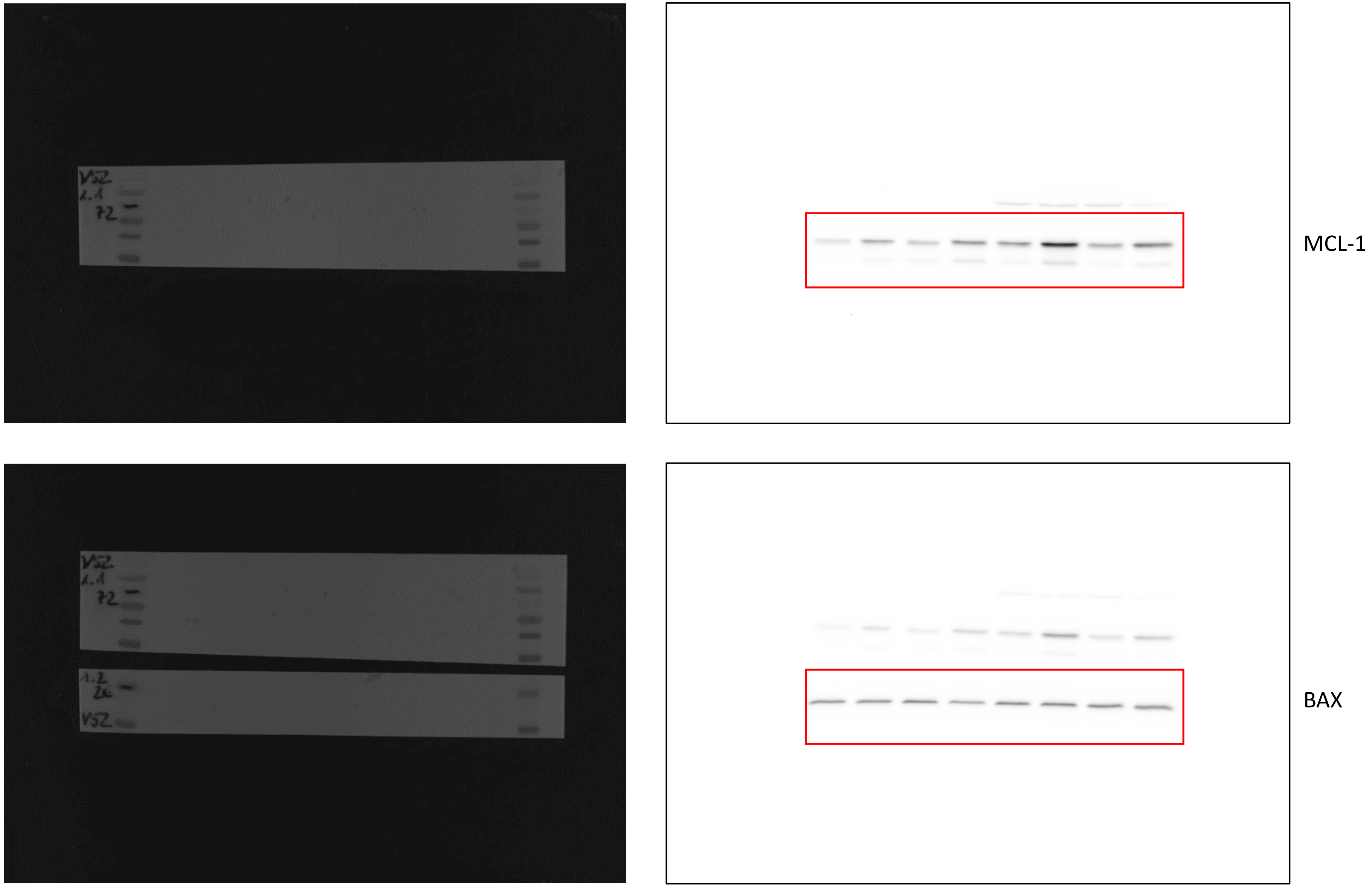

Fig. 1 D

SW982/WT

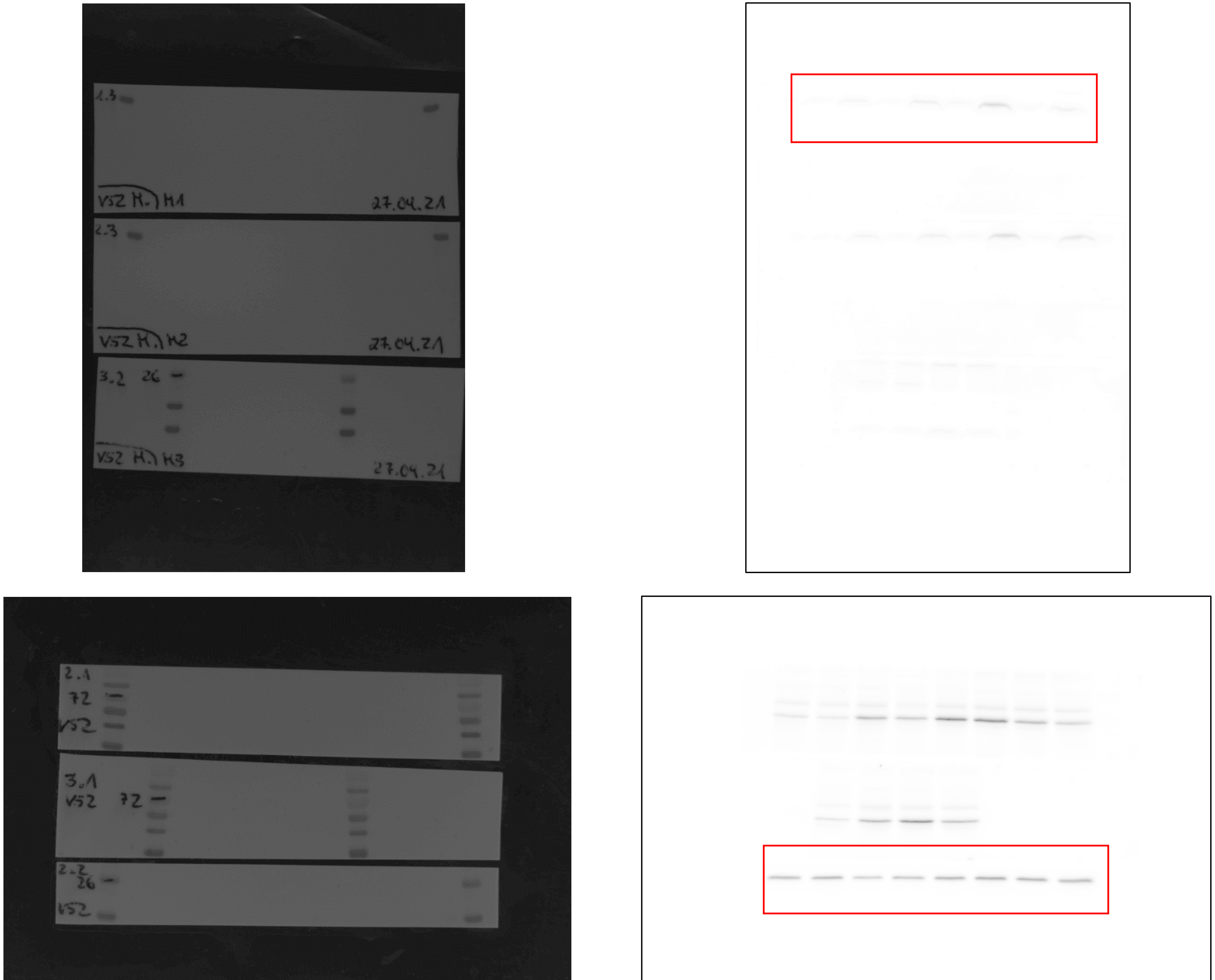

NOXA

BCL-2

Fig. 1 D

SW982/WT

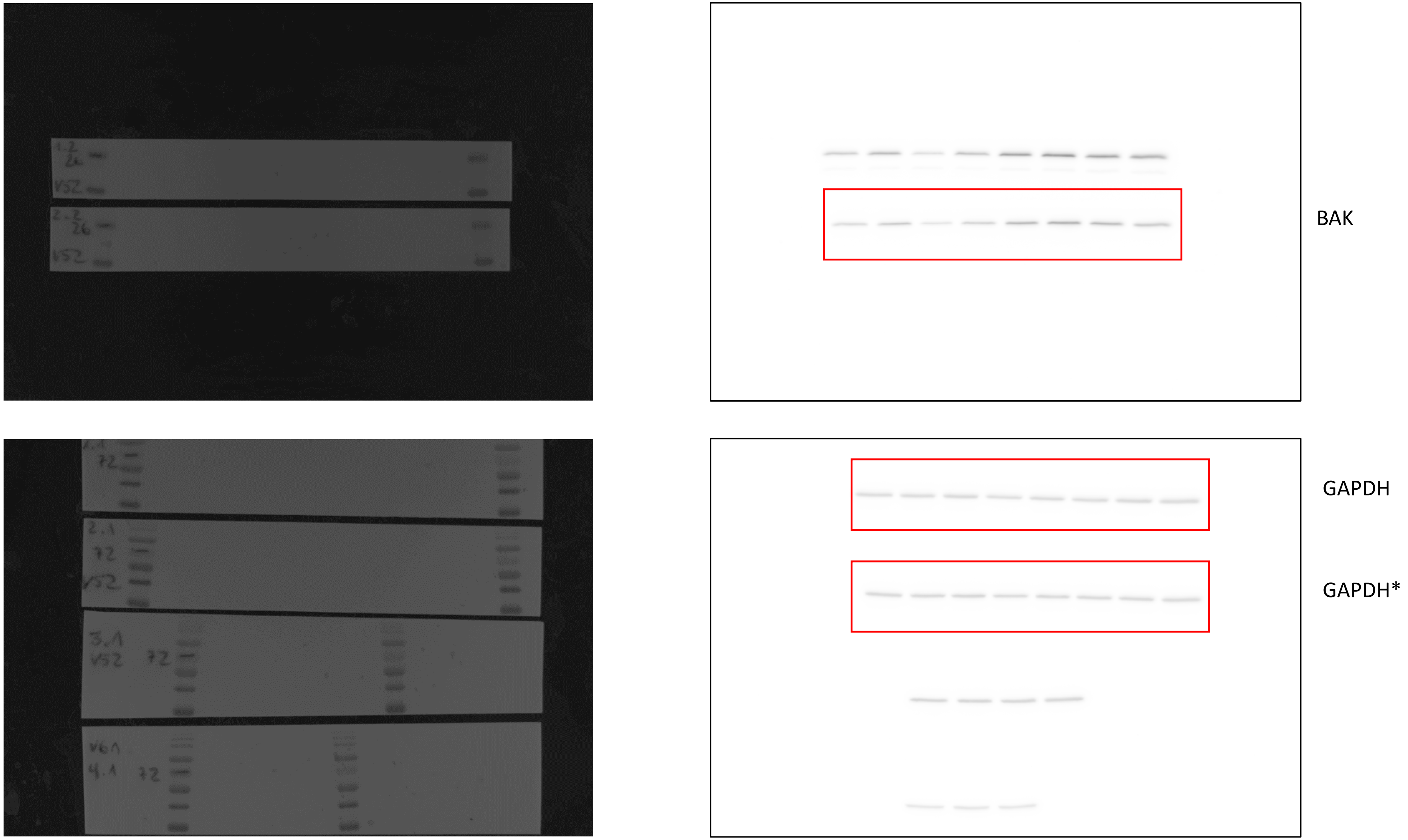

Fig. 1 D

SW982/BAK^{KO}

SW982/BAX^{KO}

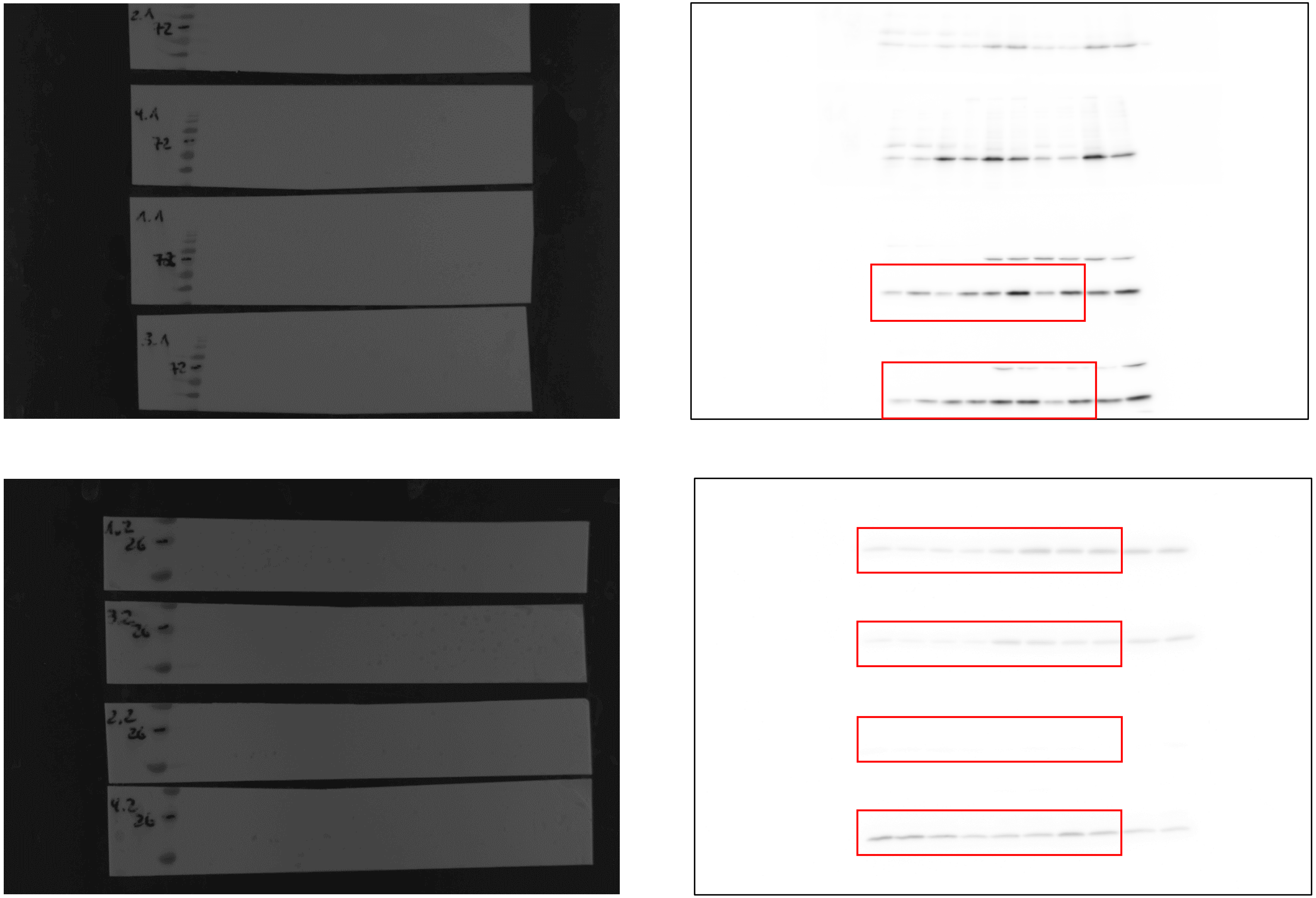

MCL-1 (SW982/BAX^{KO})

MCL-1 (SW982/BAK^{KO})

BOK (SW982/BAX^{KO})

BOK (SW982/BAK^{KO})

BAX (SW982/BAX^{KO})

BAX (SW982/BAK^{KO})

Fig. 1 D

SW982/BAK^{KO}

SW982/BAX^{KO}

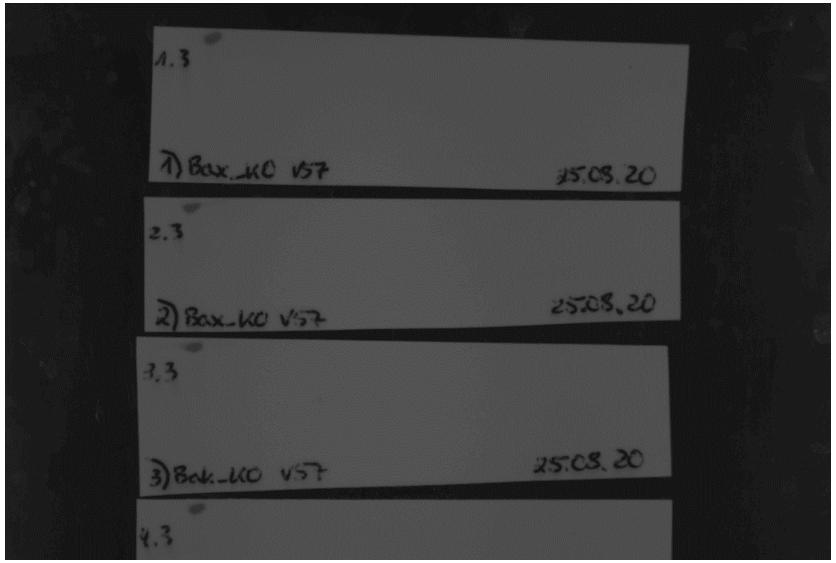

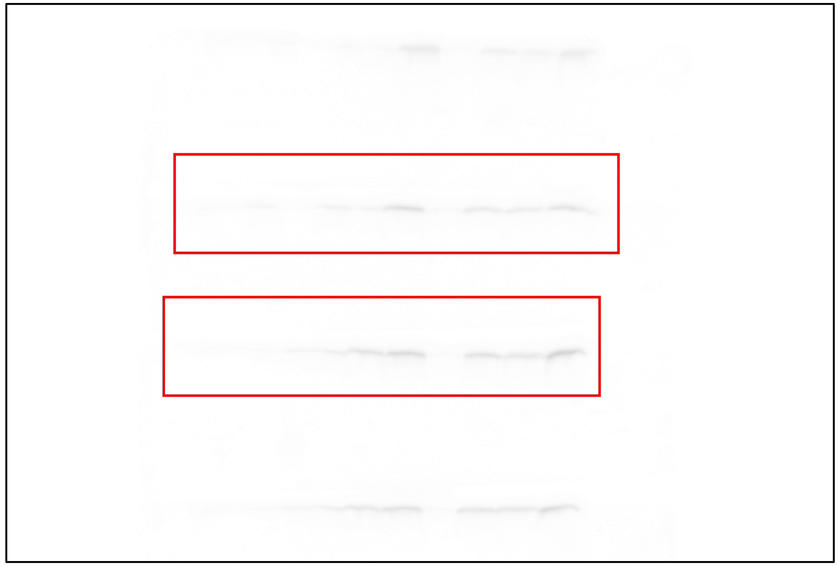

NOXA (SW982/BAX^{KO})

NOXA (SW982/BAK^{KO})

Fig. 1 D

SW982/BAK^{KO}

SW982/BAX^{KO}

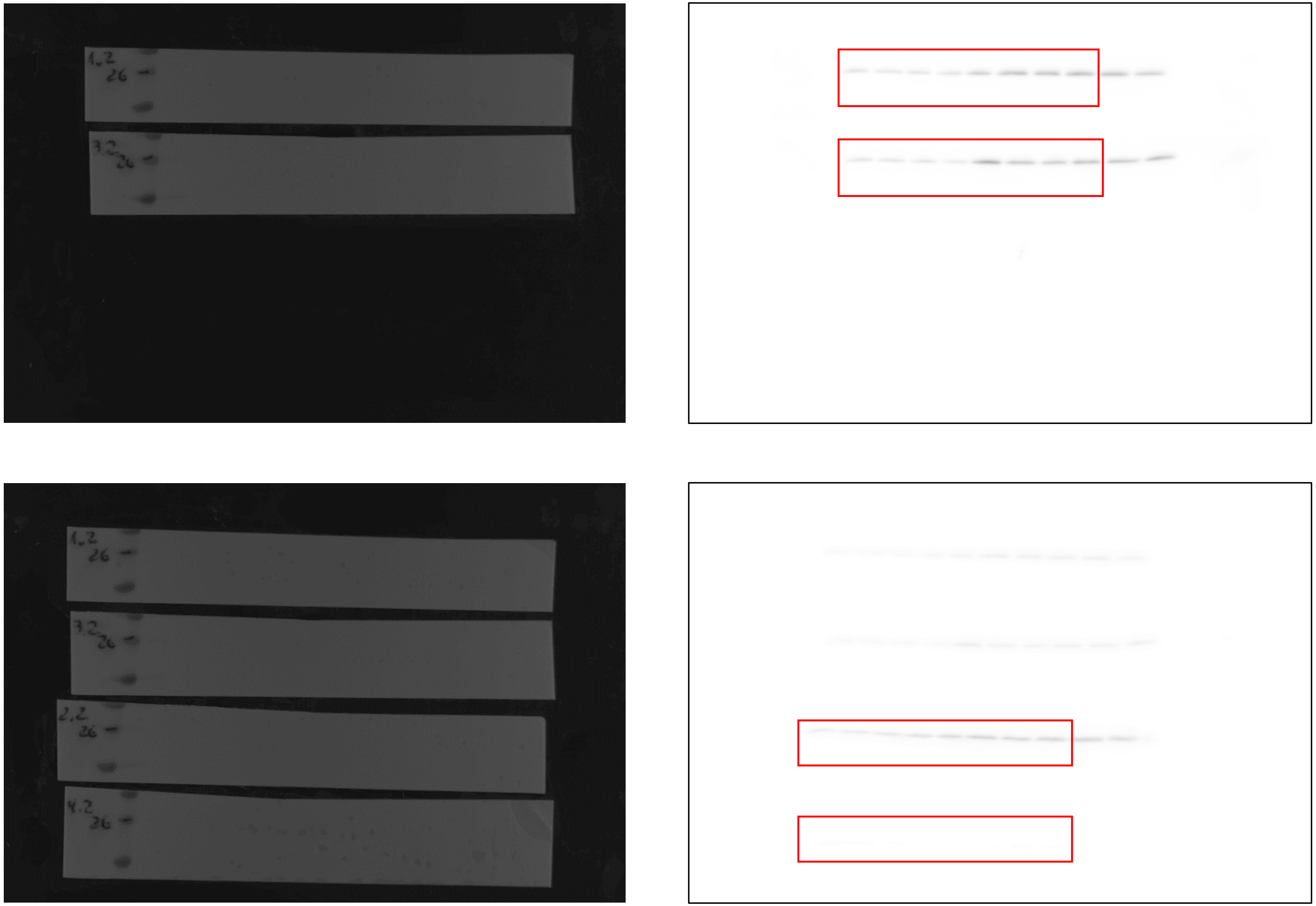

BCL-2 (SW982/BAX^{KO})

BCL-2 (SW982/BAK^{KO})

BAK (SW982/BAX^{KO})

BAK (SW982/BAK^{KO})

Fig. 1 D

SW982/BAK^{KO}

SW982/BAX^{KO}

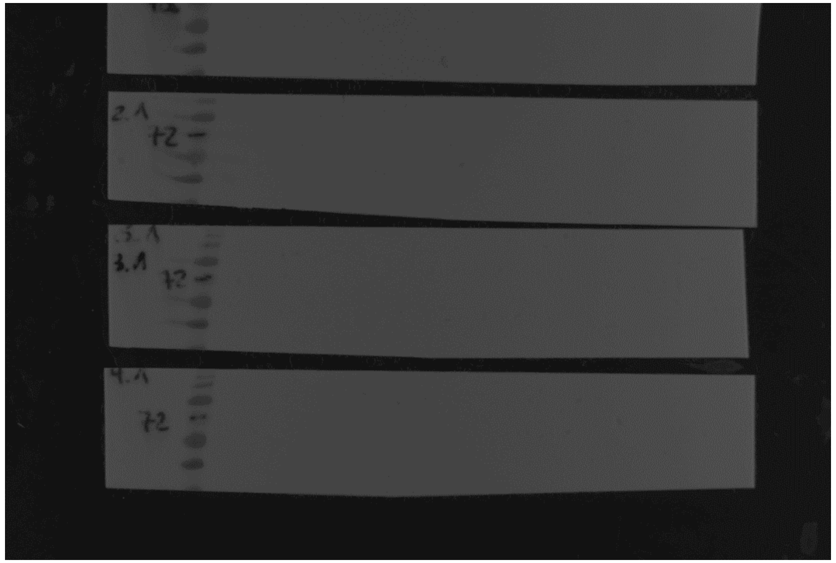

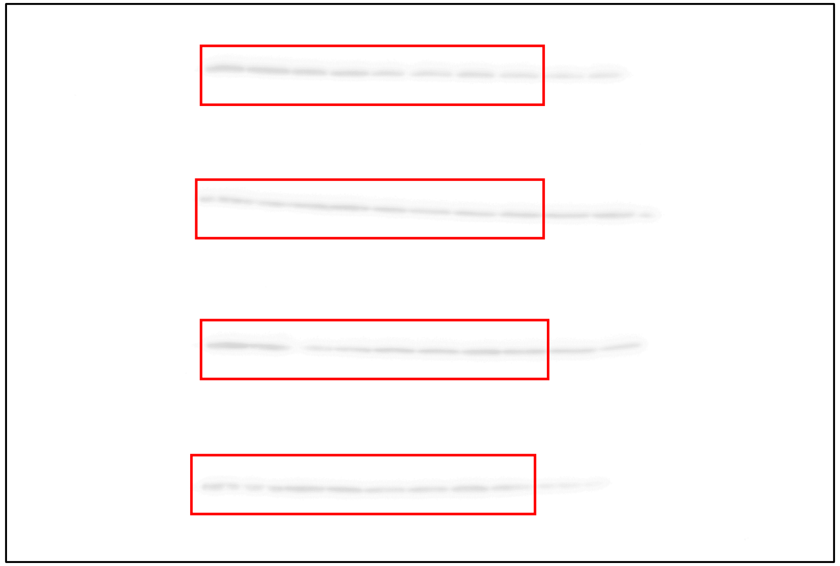

GADPH* (SW982/BAX^{KO})

GADPH** (SW982/BAX^{KO})

GAPDH (SW982/BAK^{KO})

GAPDH* (SW982/BAK^{KO})

Fig. 2 G-I

SW982/NOXA^{KD}

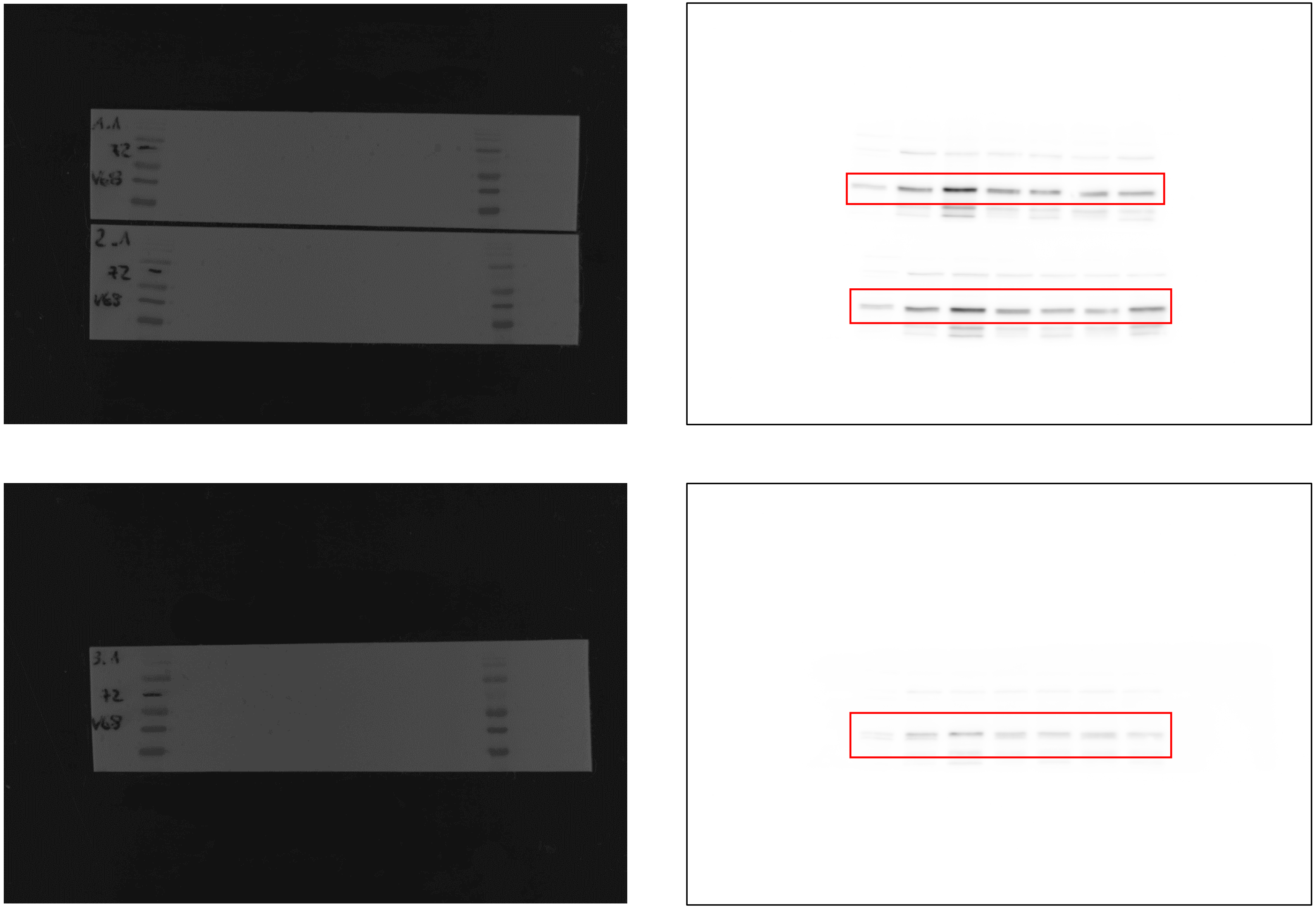

MCL-1 (Fig. 2 G)

MCL-1 (Fig. 2 H)

MCL-1 (Fig. 2 I)

Fig. 2 G-I

SW982/NOXA^{KD}

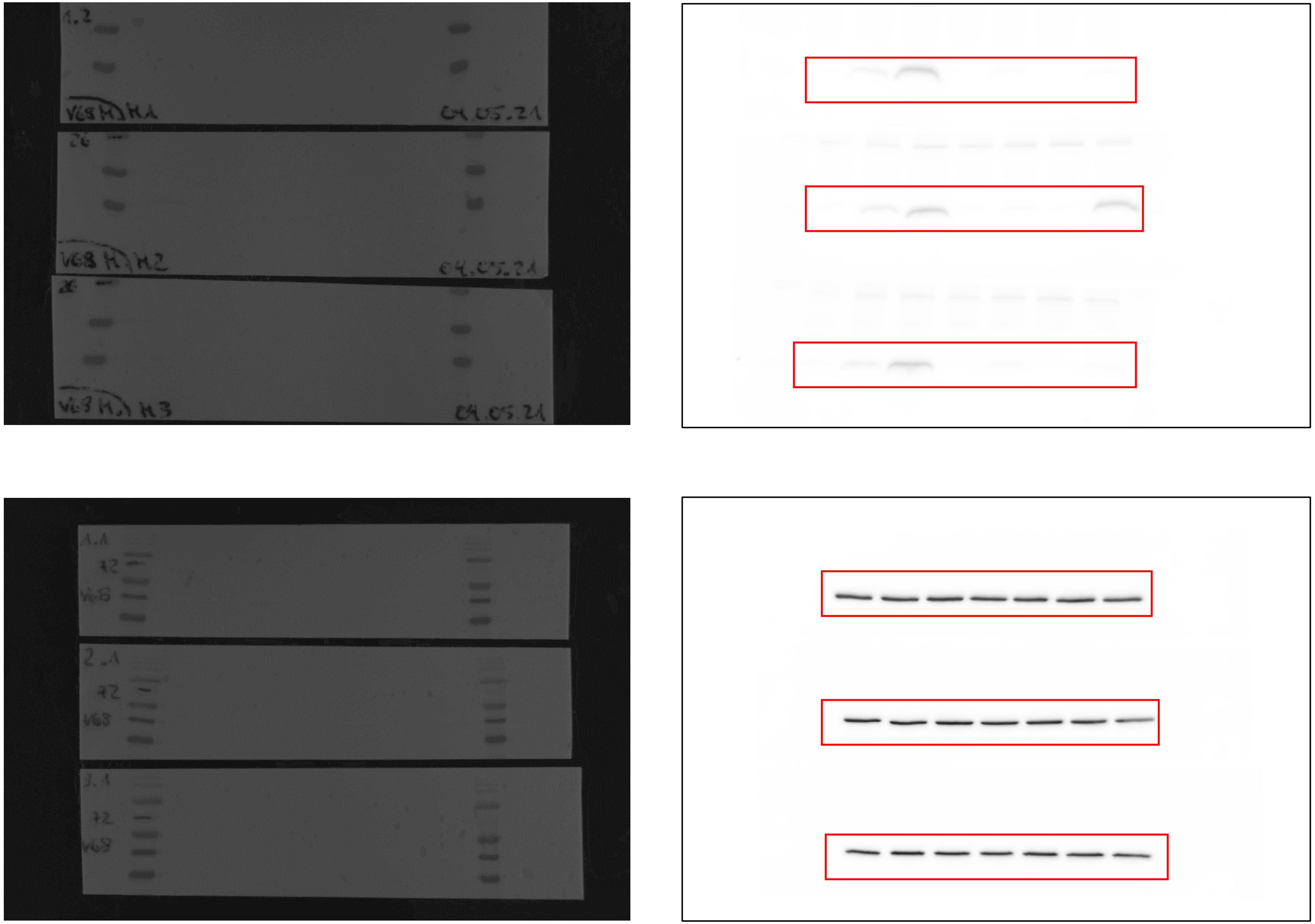

NOXA (Fig. 2 G)

NOXA (Fig. 2 H)

NOXA (Fig. 2 I)

ß-ACTIN (Fig. 2 G)

ß-ACTIN (Fig. 2 H)

ß-ACTIN (Fig. 2 I)

Fig. 3 A

SW982/WT

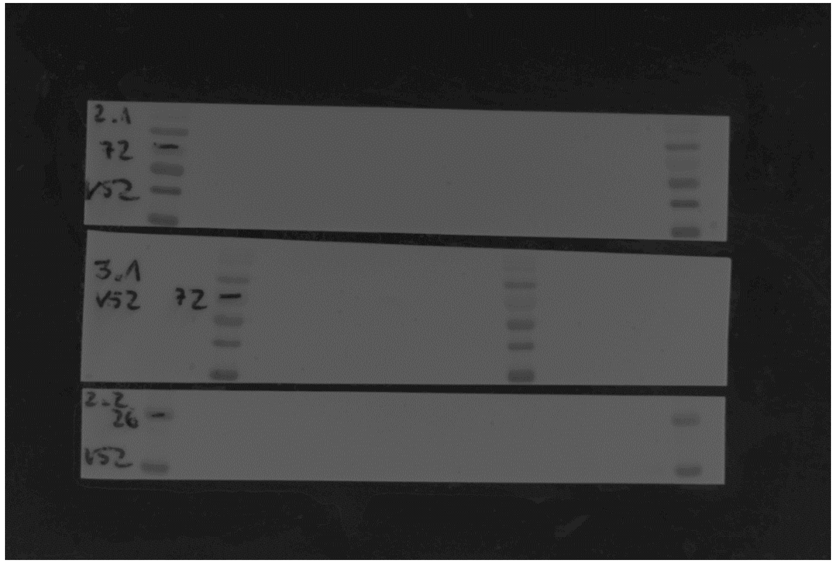

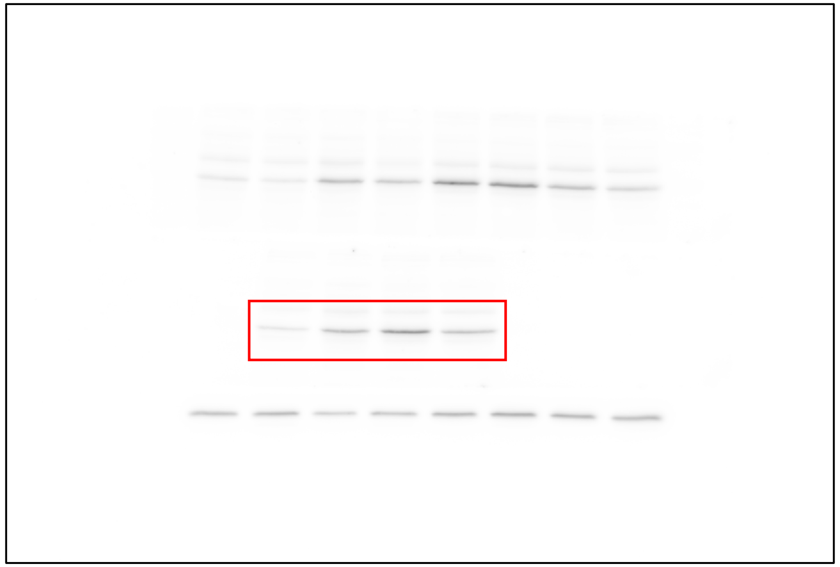

TP53

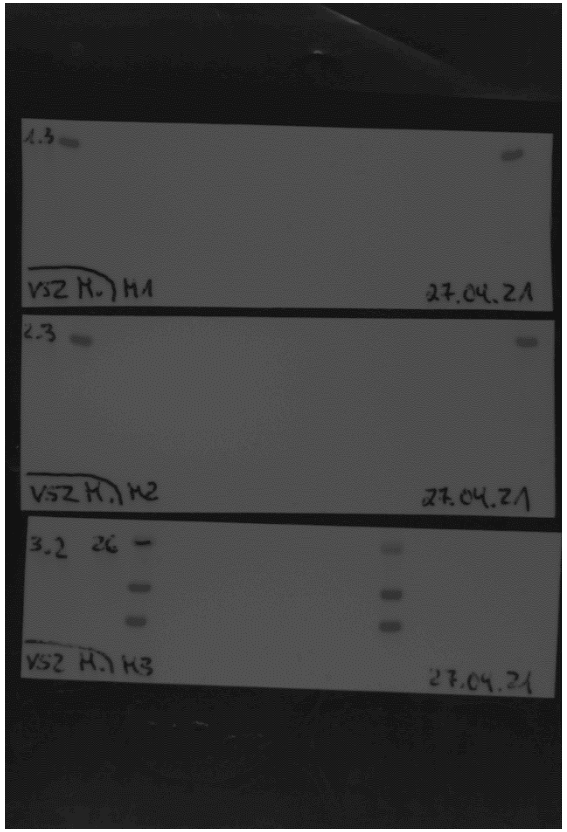

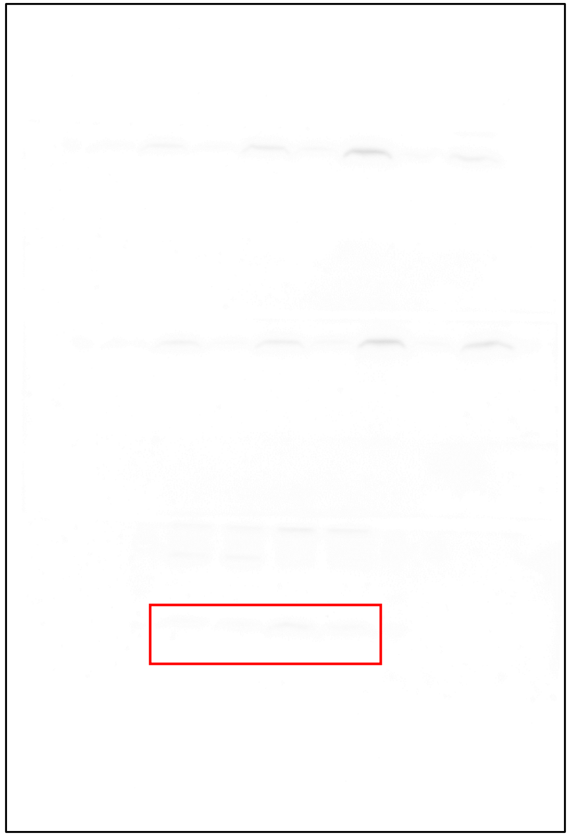

NOXA

Fig. 3 A

SW982/WT

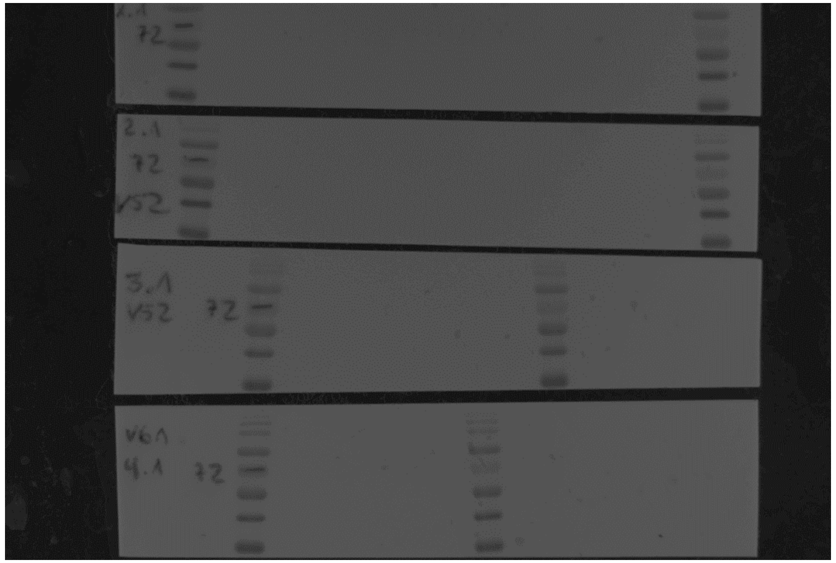

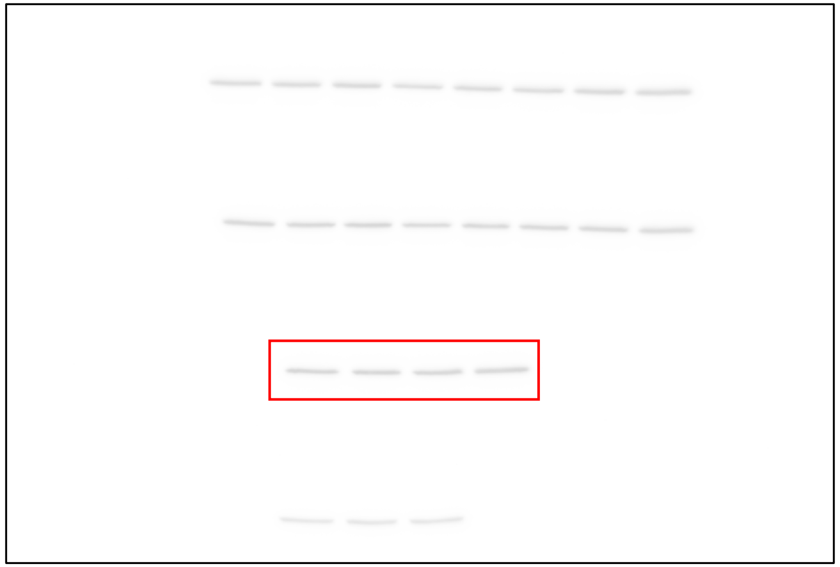

GAPDH

Fig. 3 C

SW982/WT

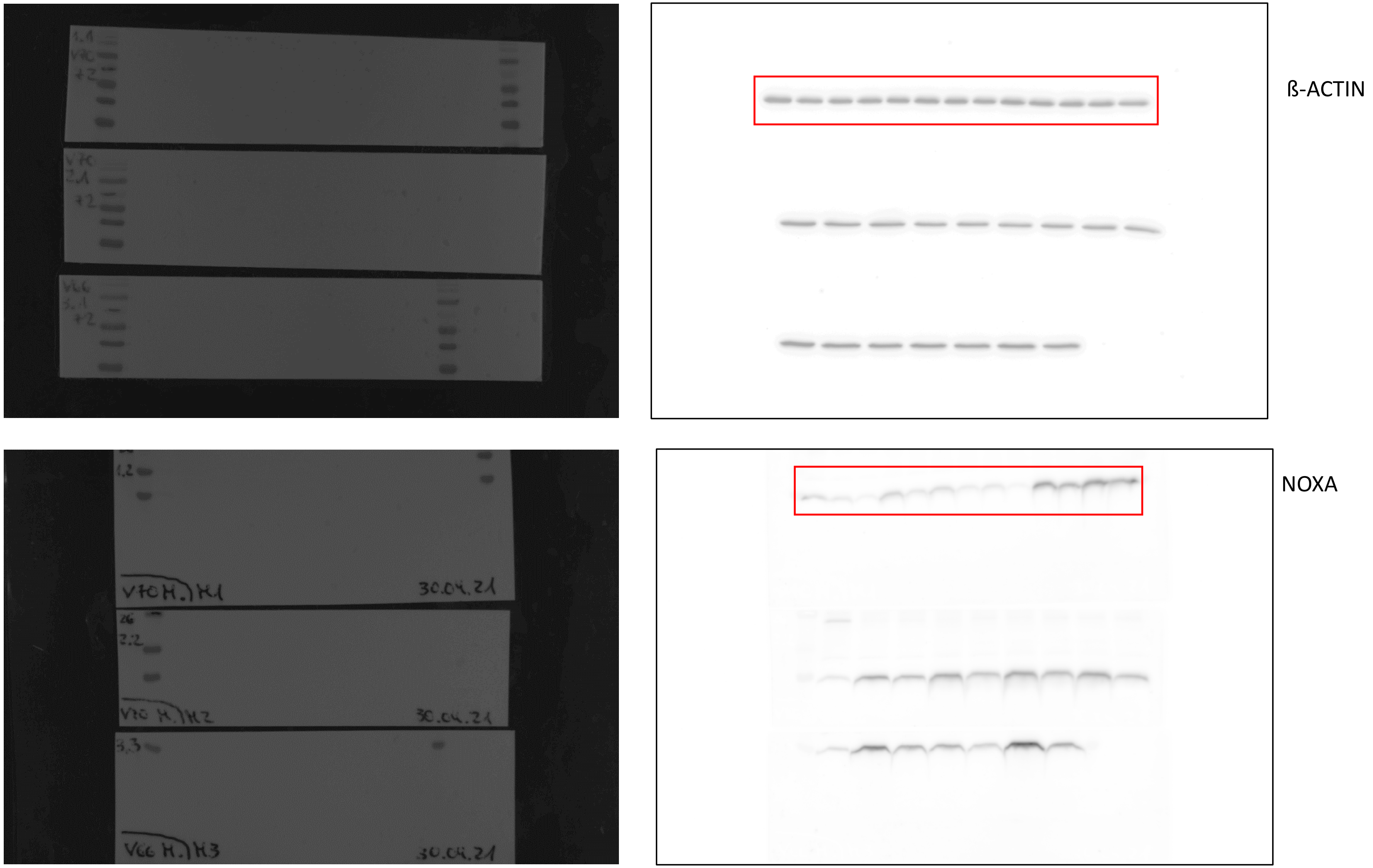

Fig. 3 C

SW982/WT

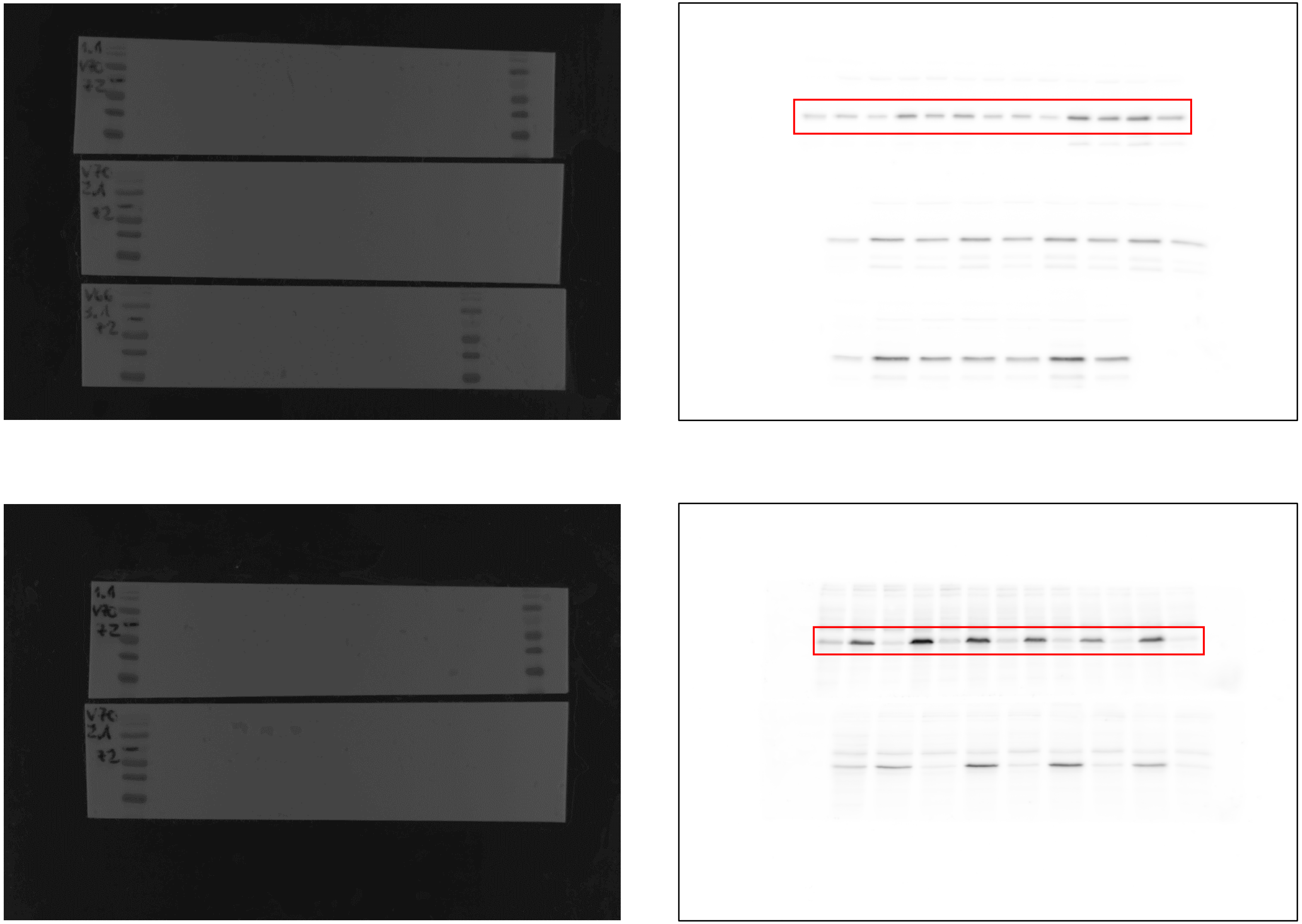

MCL-1

TP53

Fig. 3 D

SW982/WT

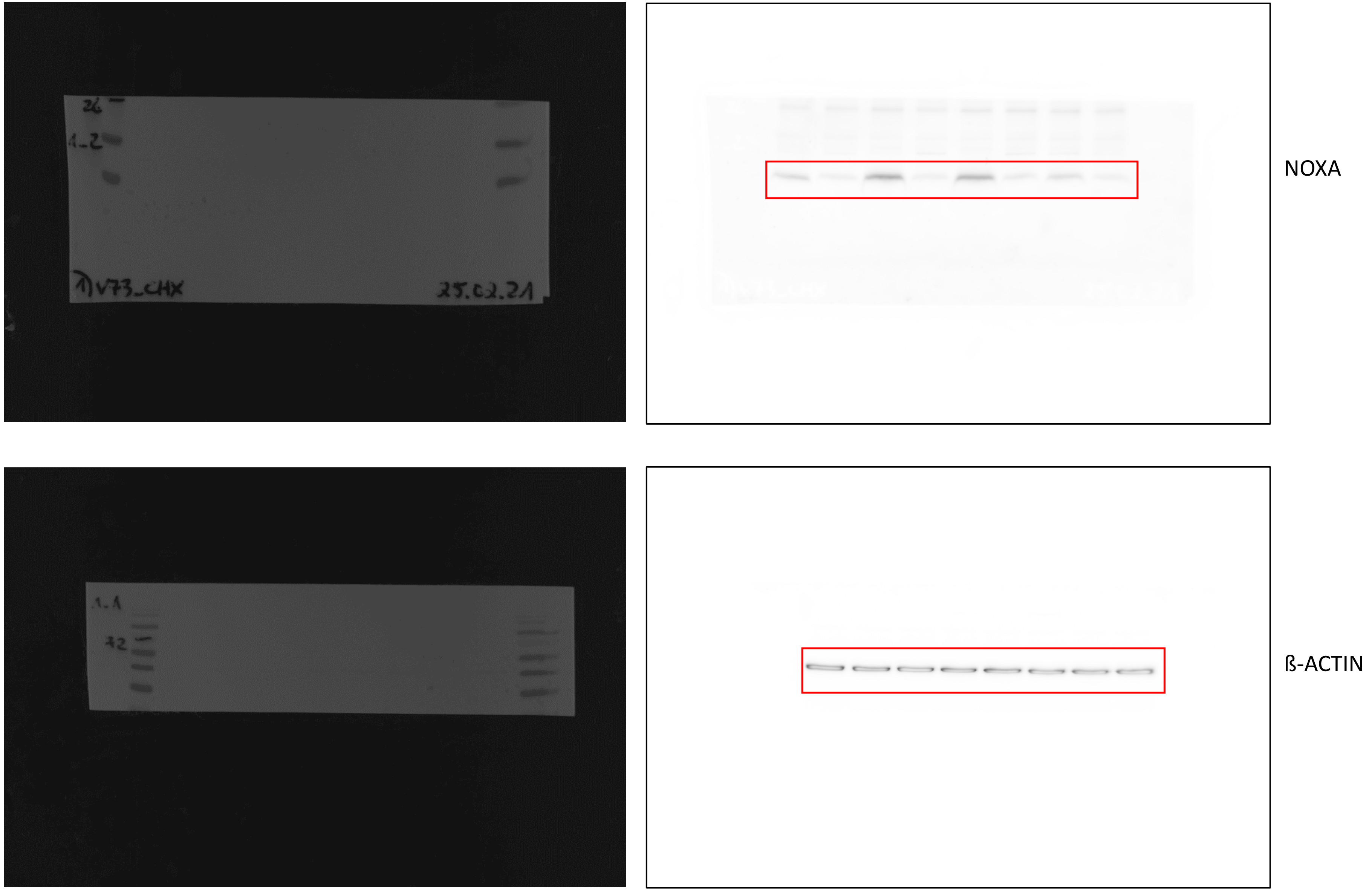

Fig. 3 D

SW982/WT

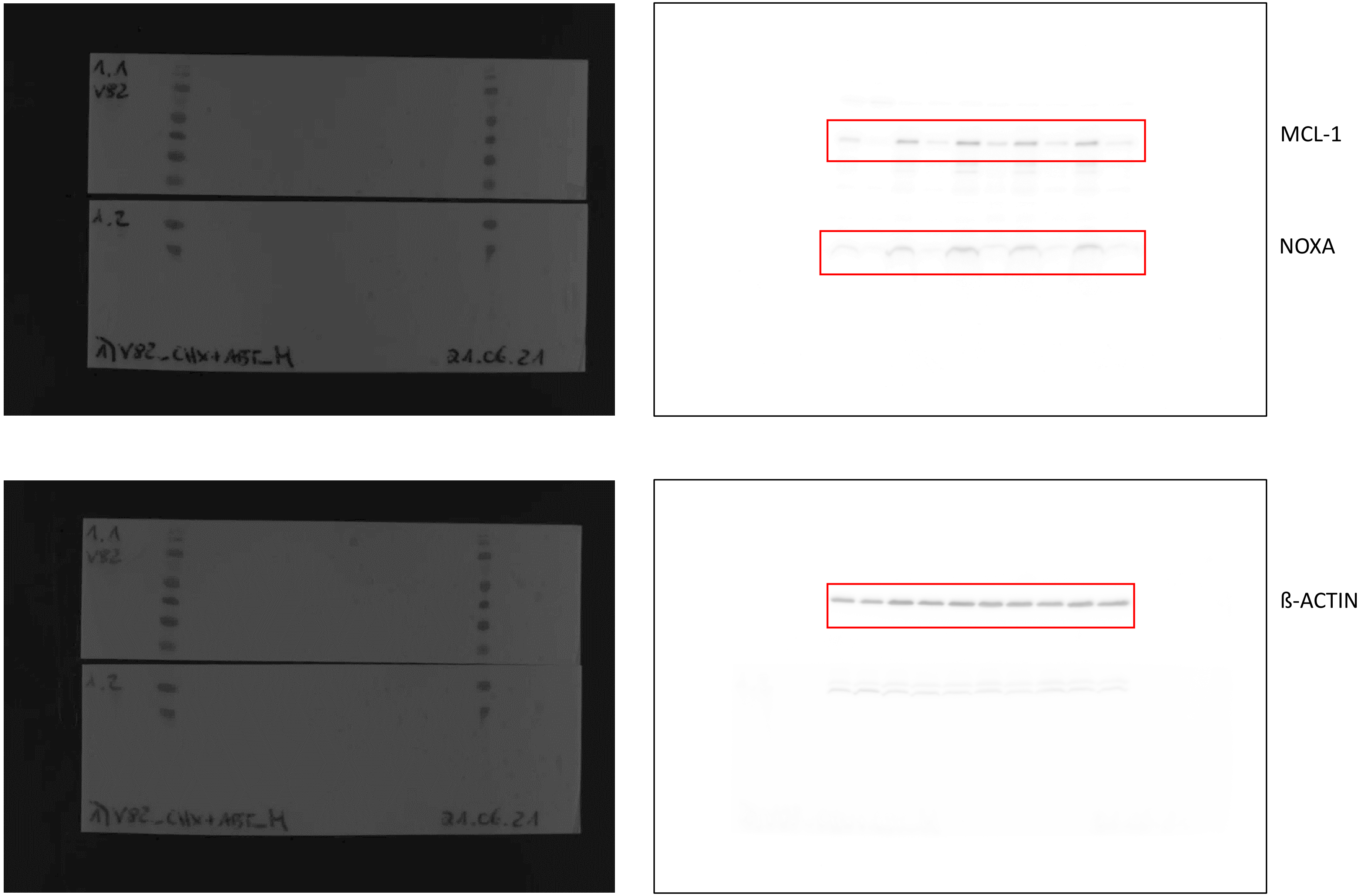

Fig. 3 E

SW982/WT

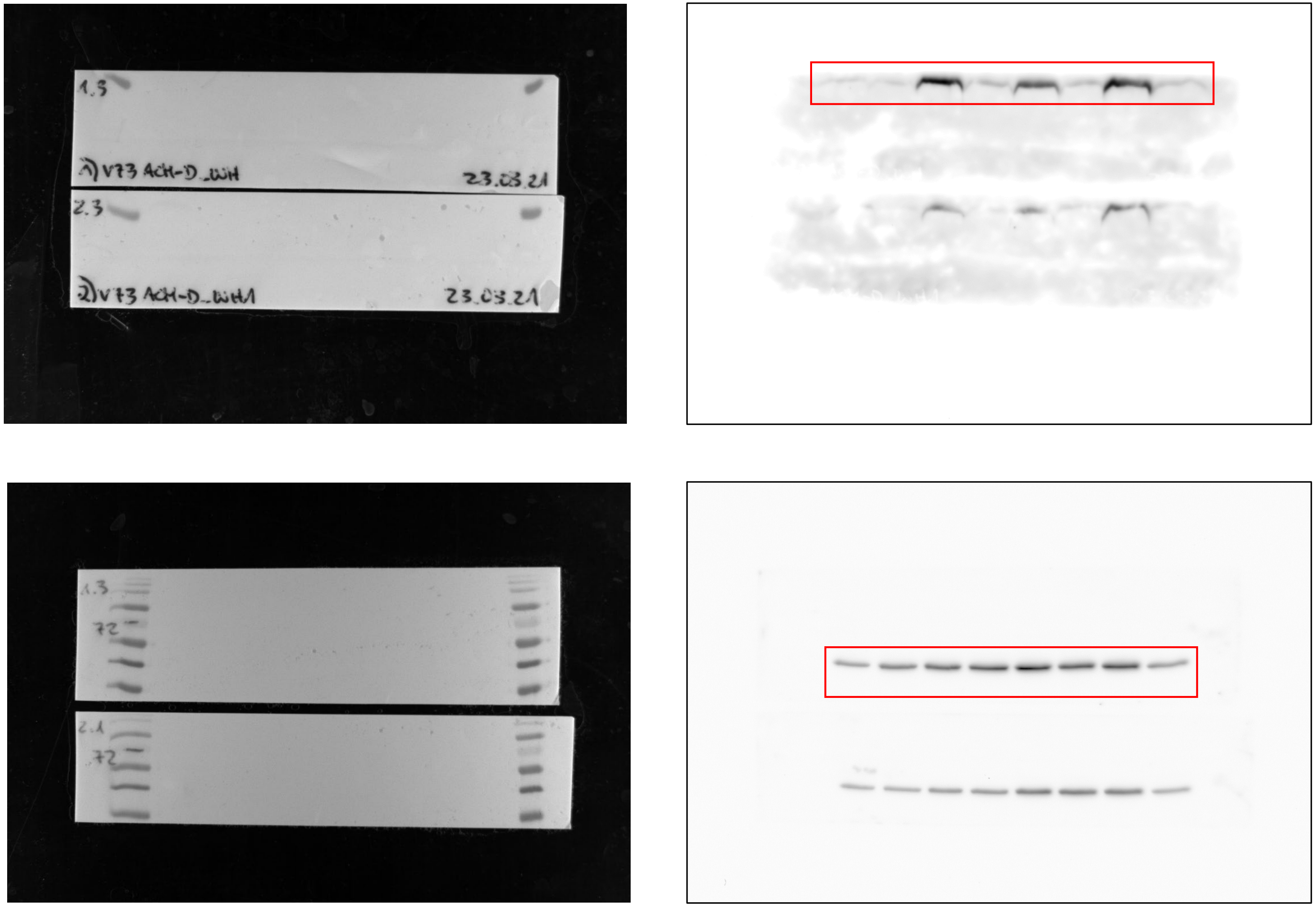

Fig. 3 F

SW982/WT

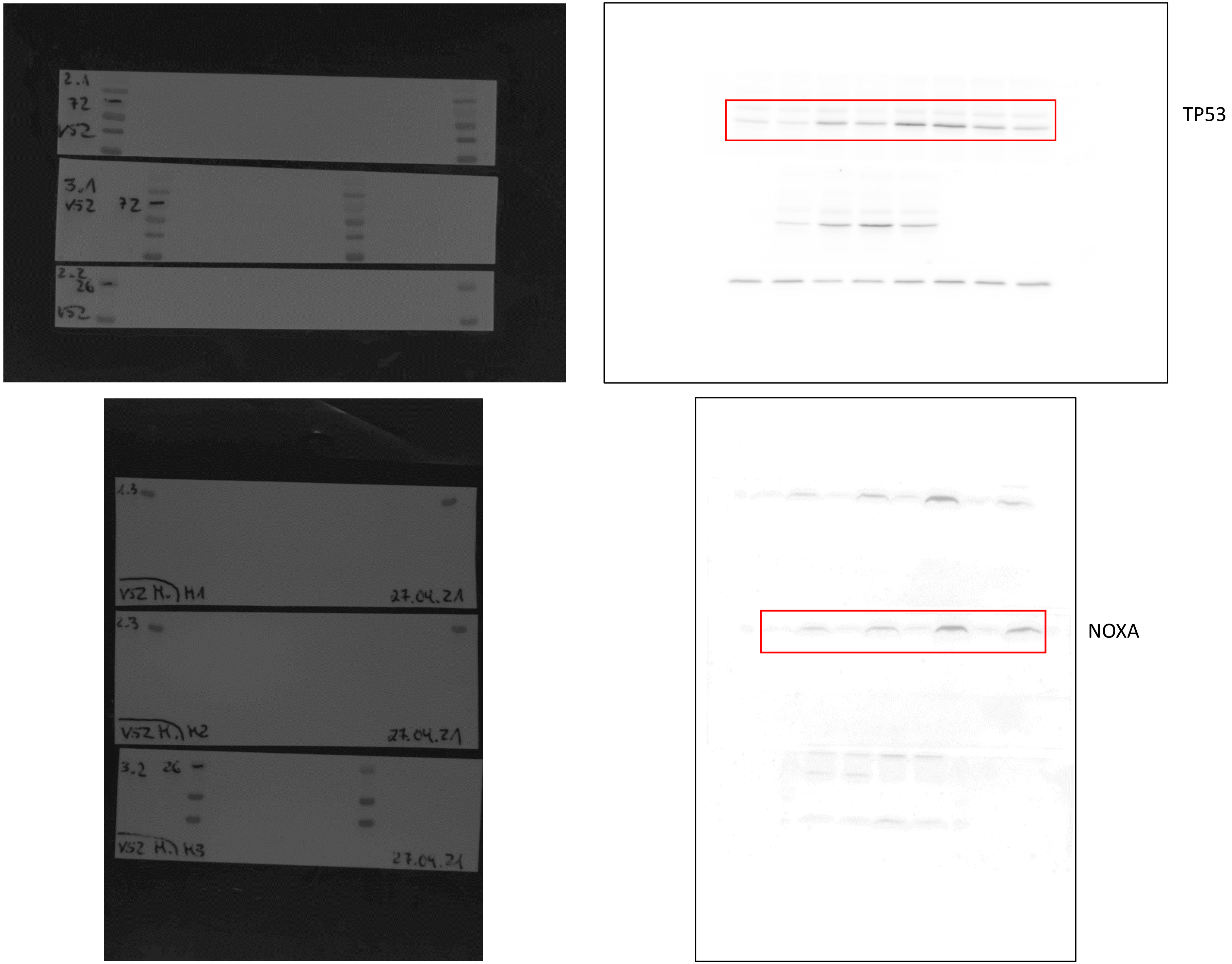

Fig. 3 F

SW982/WT

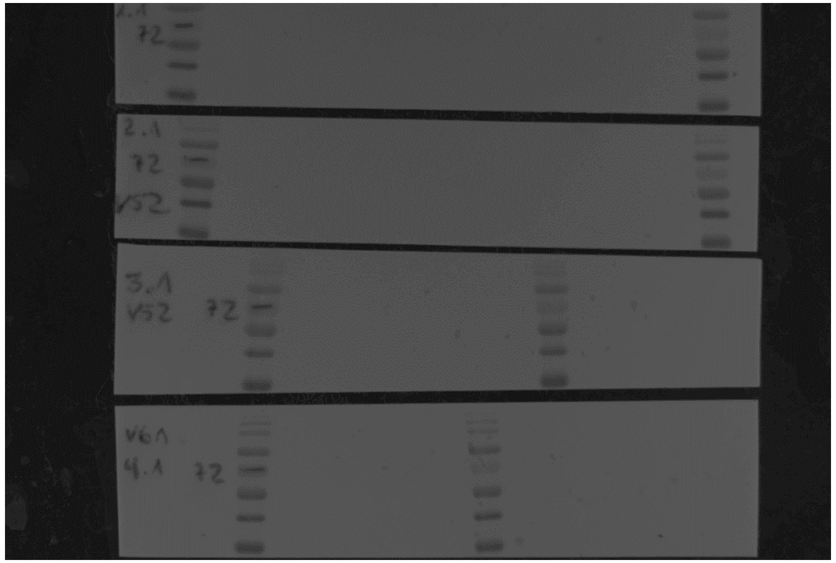

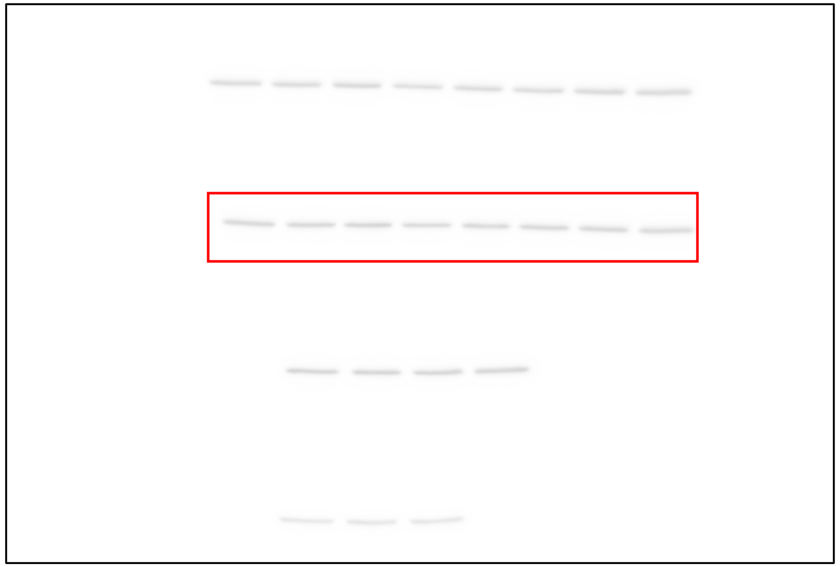

GAPDH

Fig. 3 H

SW982/WT

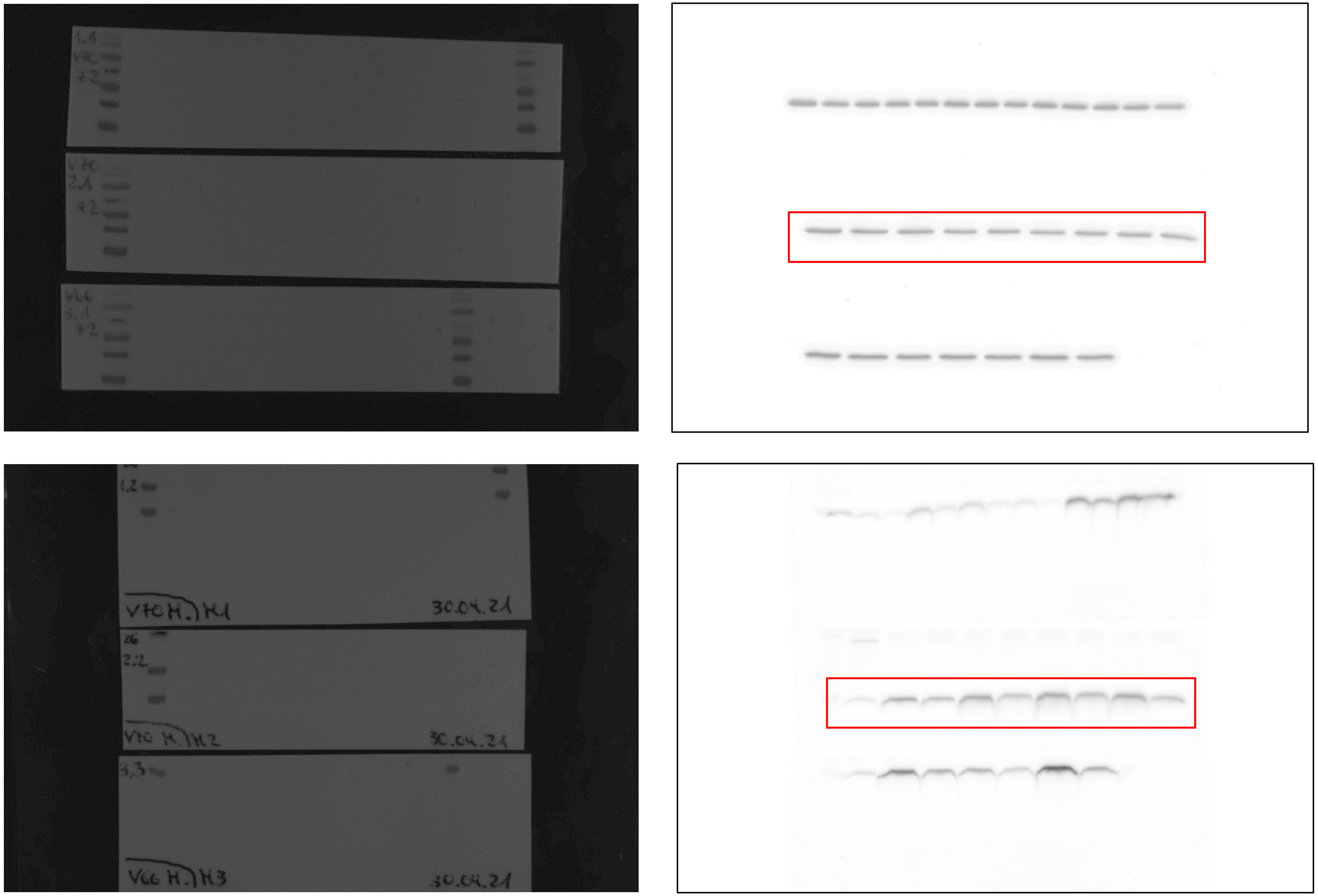

ß-ACTIN

NOXA

Fig. 3 H

SW982/WT

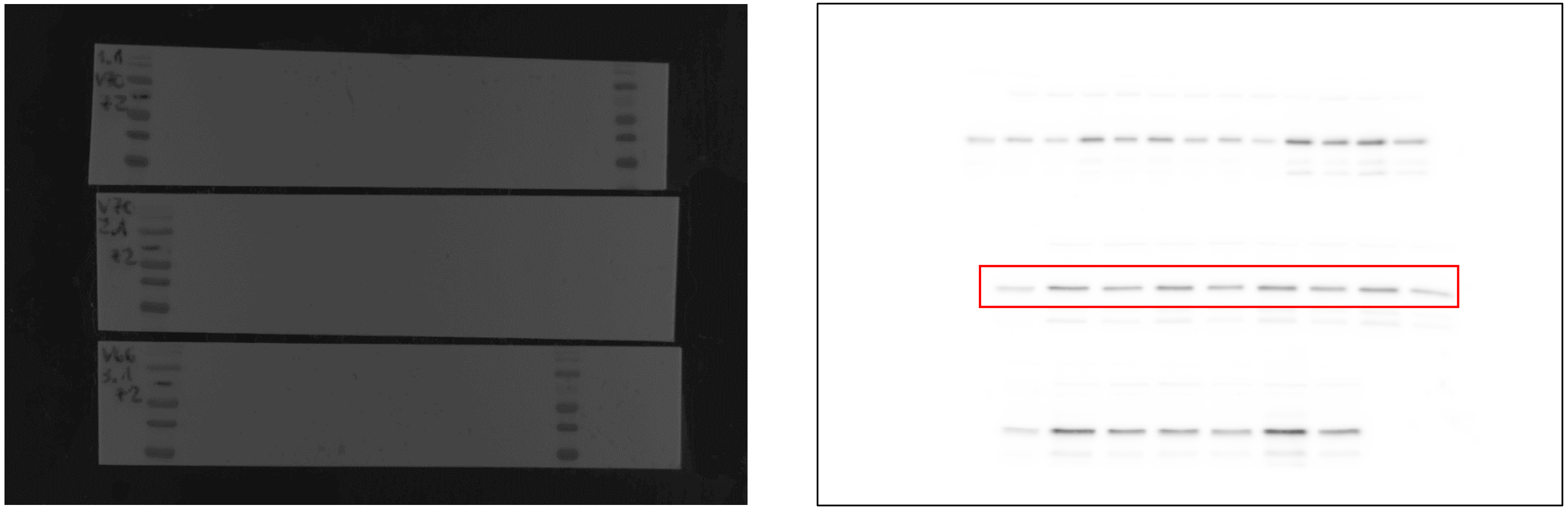

MCL-1

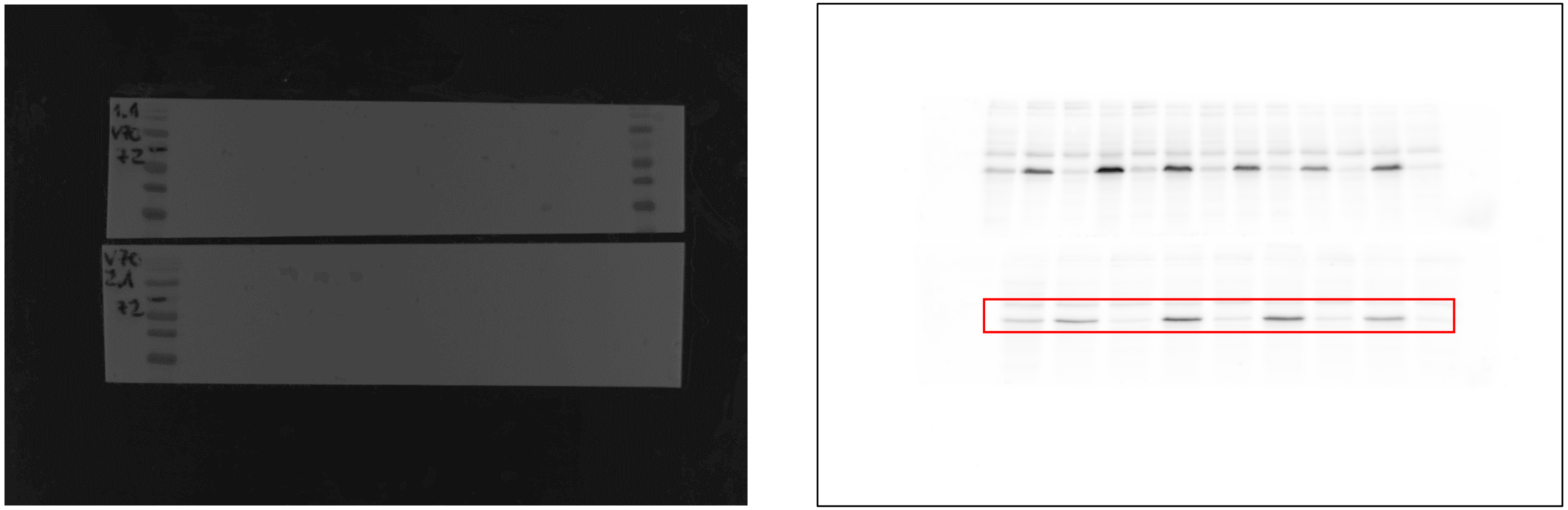

TP53

Fig. 4 A

SW982/WT

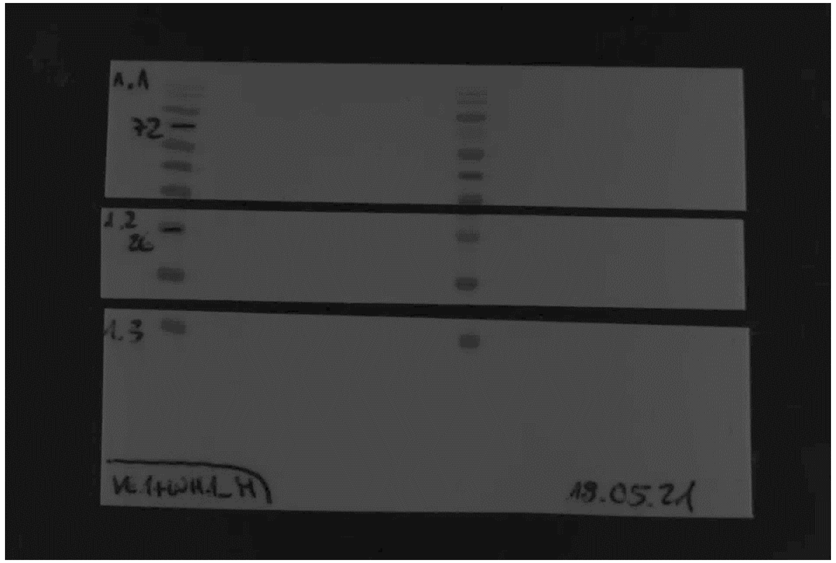

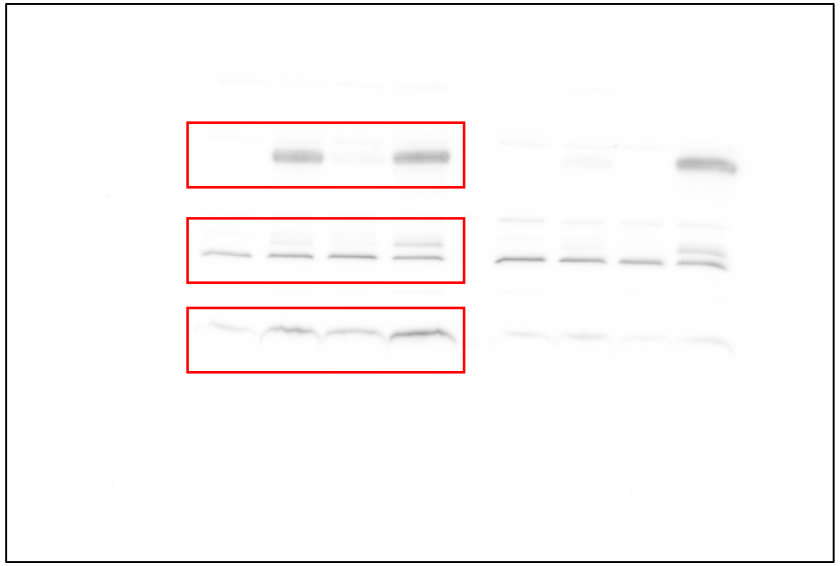

ATF4

ATF3

NOXA

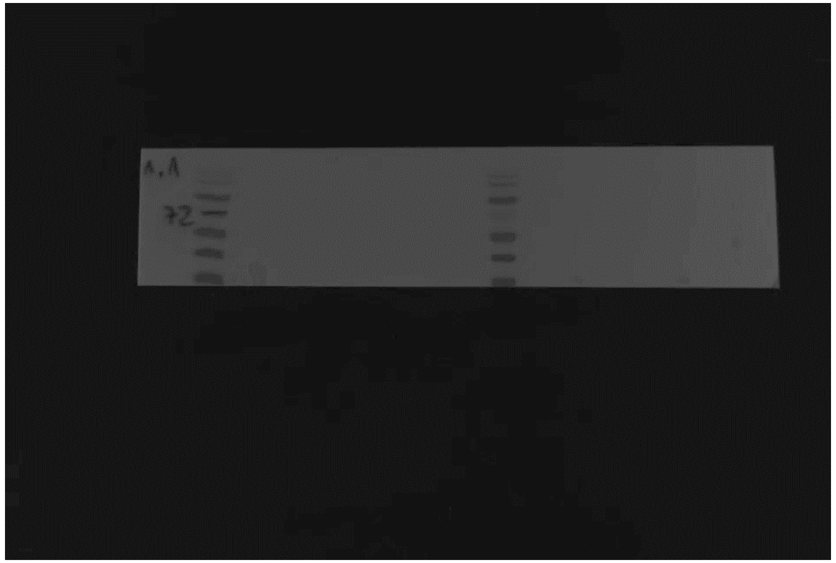

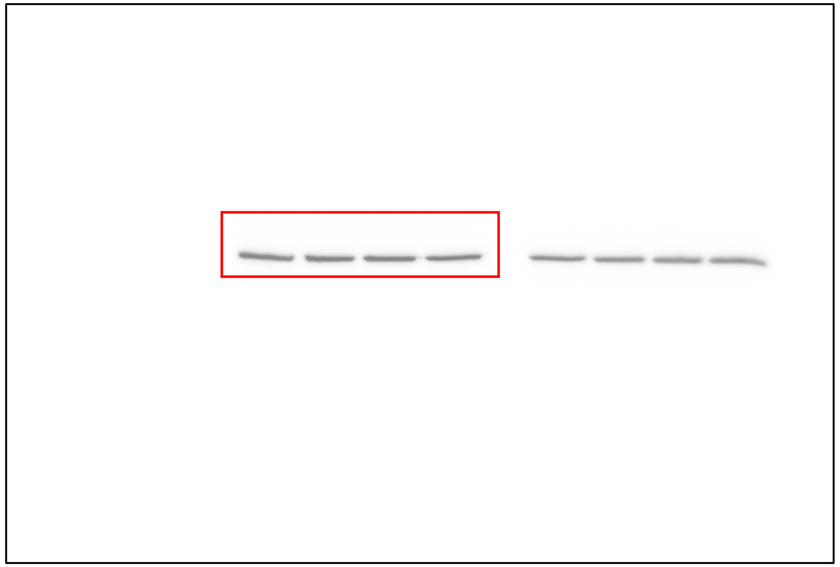

ß-ACTIN

Fig. 4 E

SW982/WT

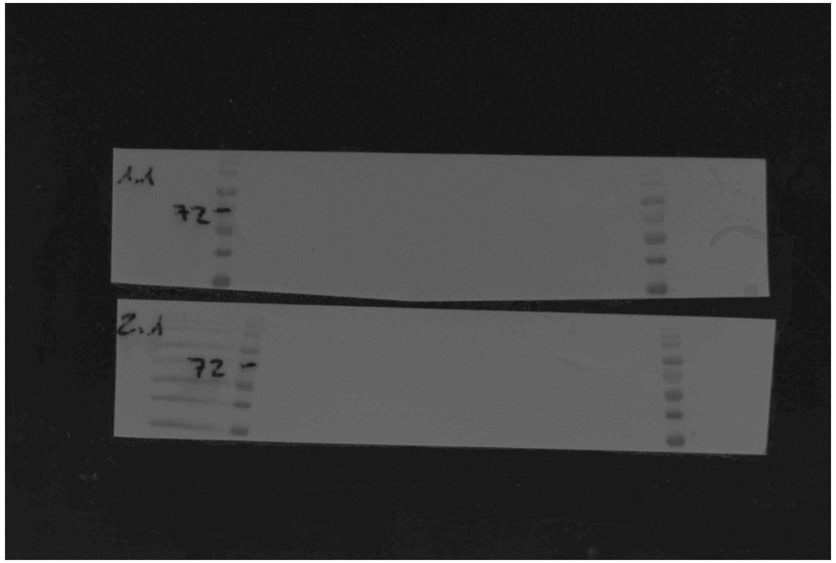

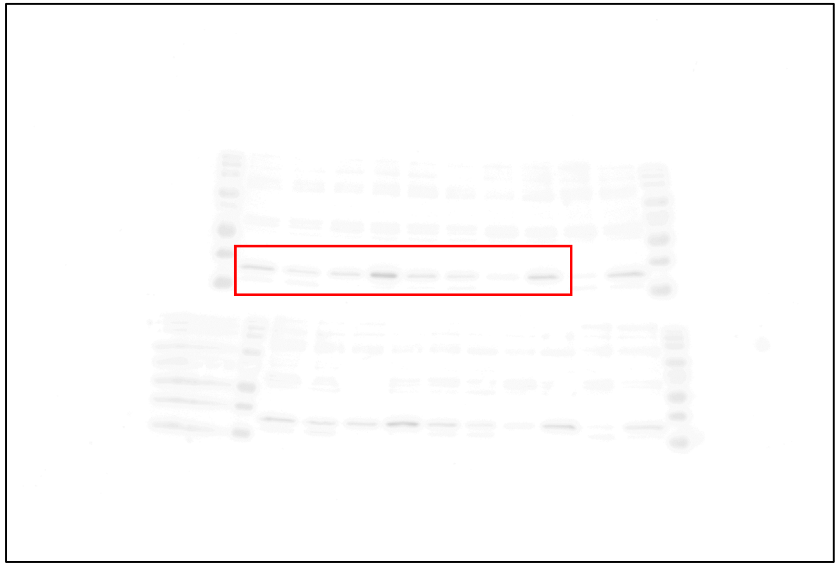

(P)-eIF2a

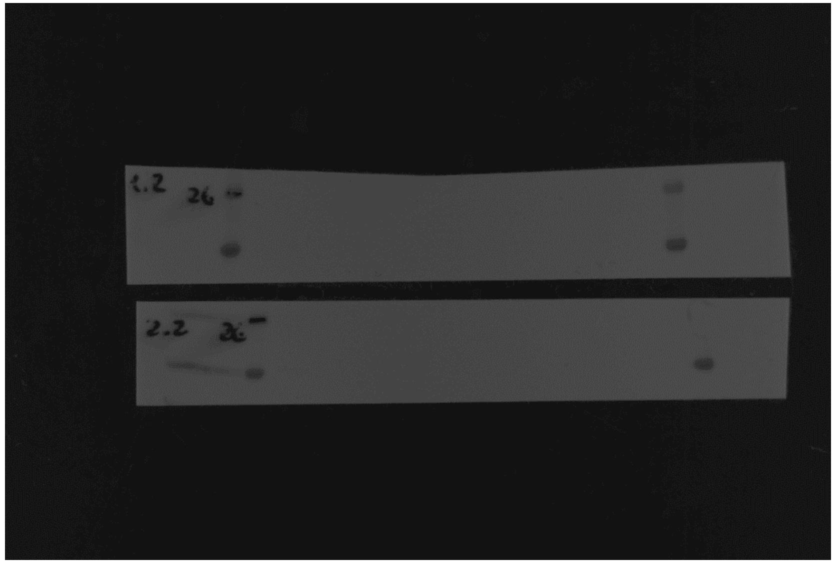

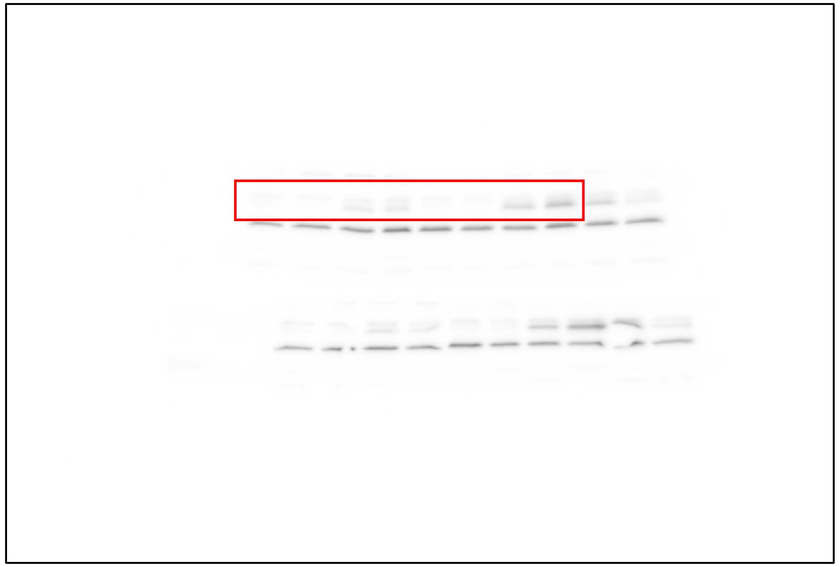

ATF3

Fig. 4 E

SW982/WT

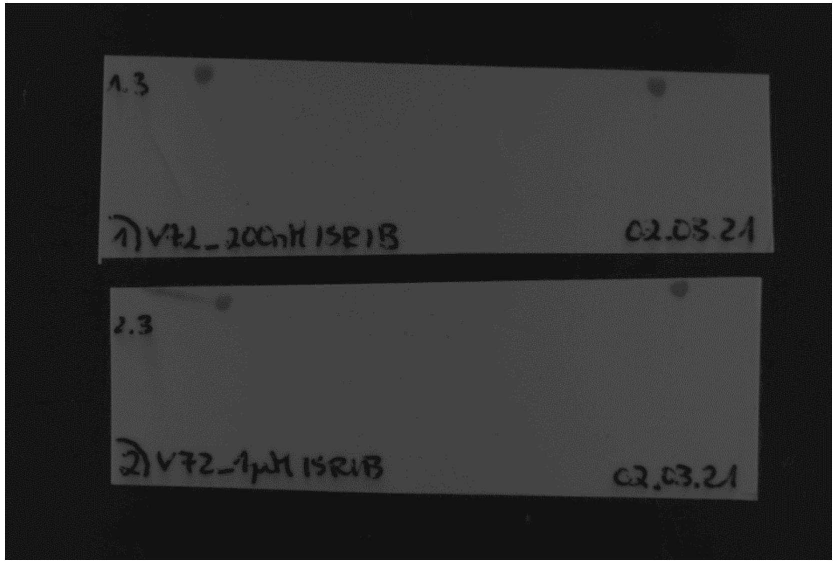

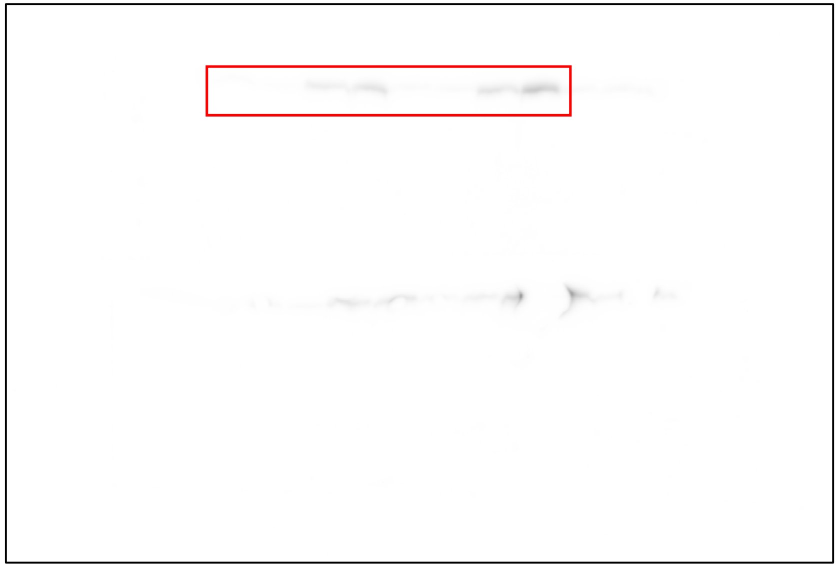

NOXA

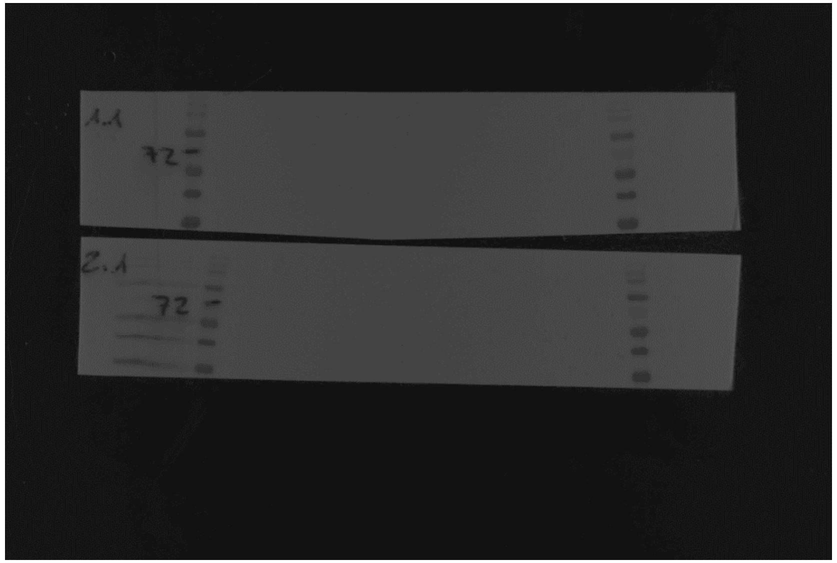

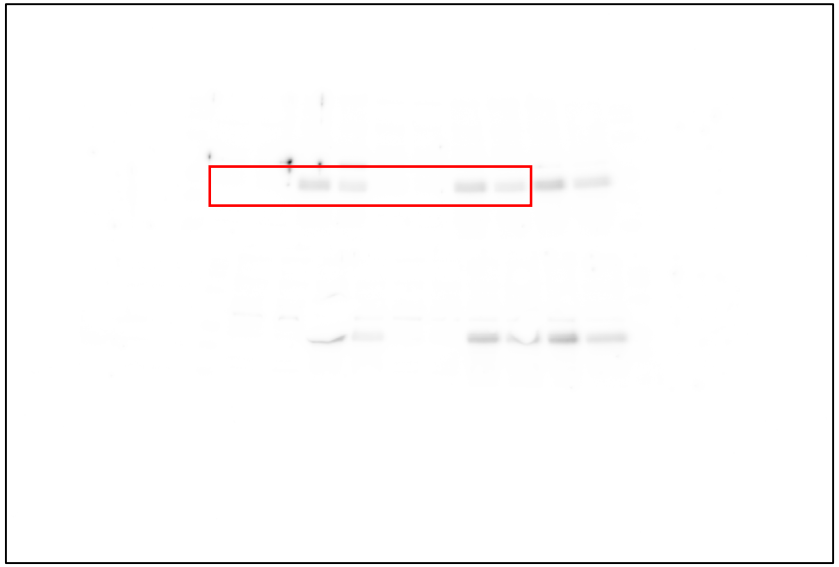

ATF4

Fig. 4 E

SW982/WT

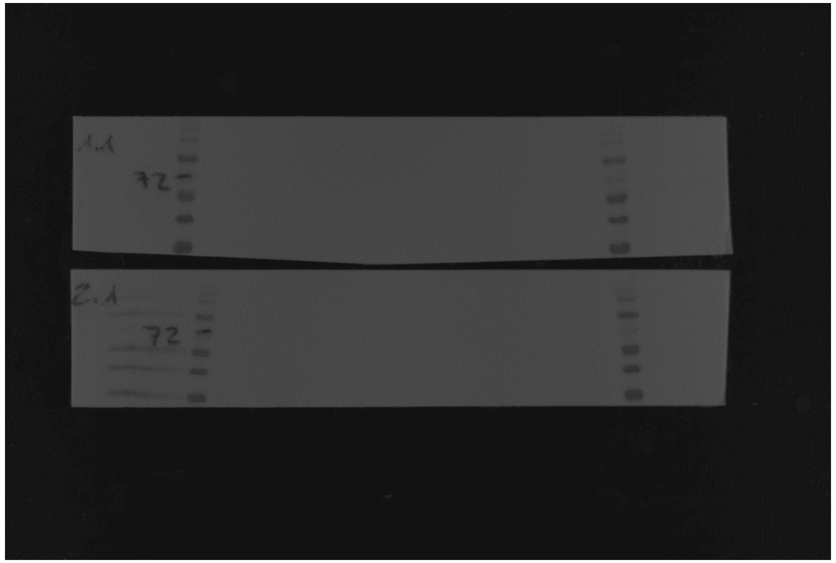

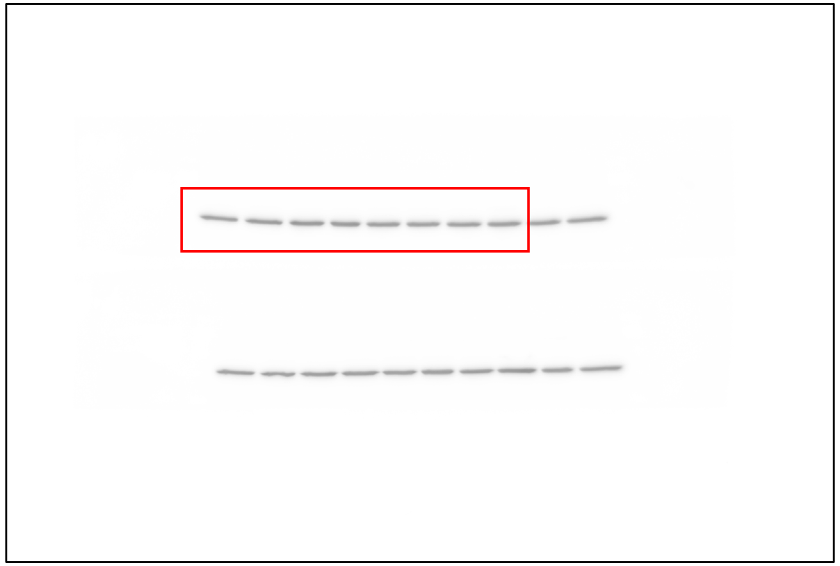

ß-ACTIN

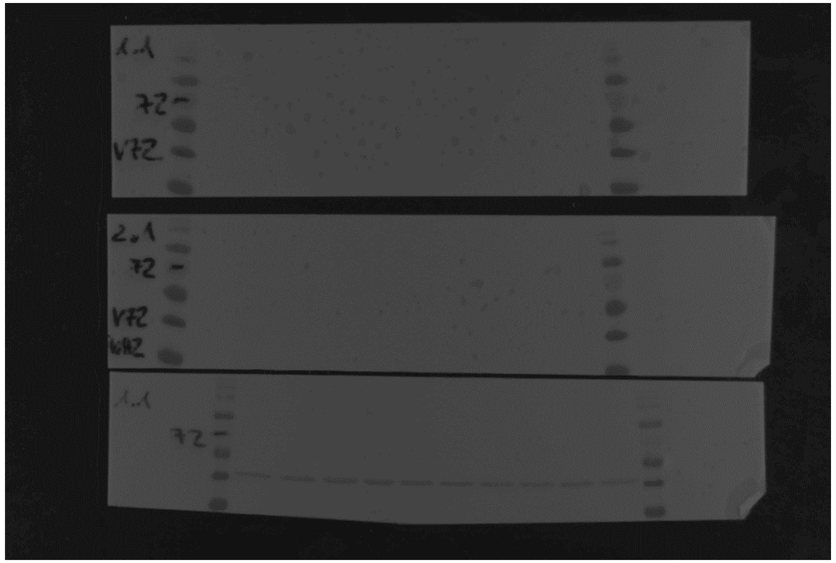

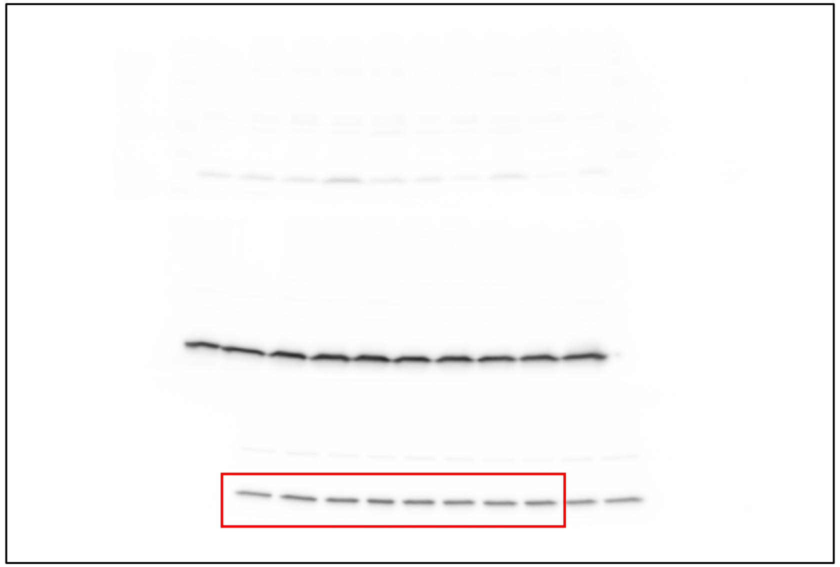

eIF2a

Fig. 5 A

SW982/WT

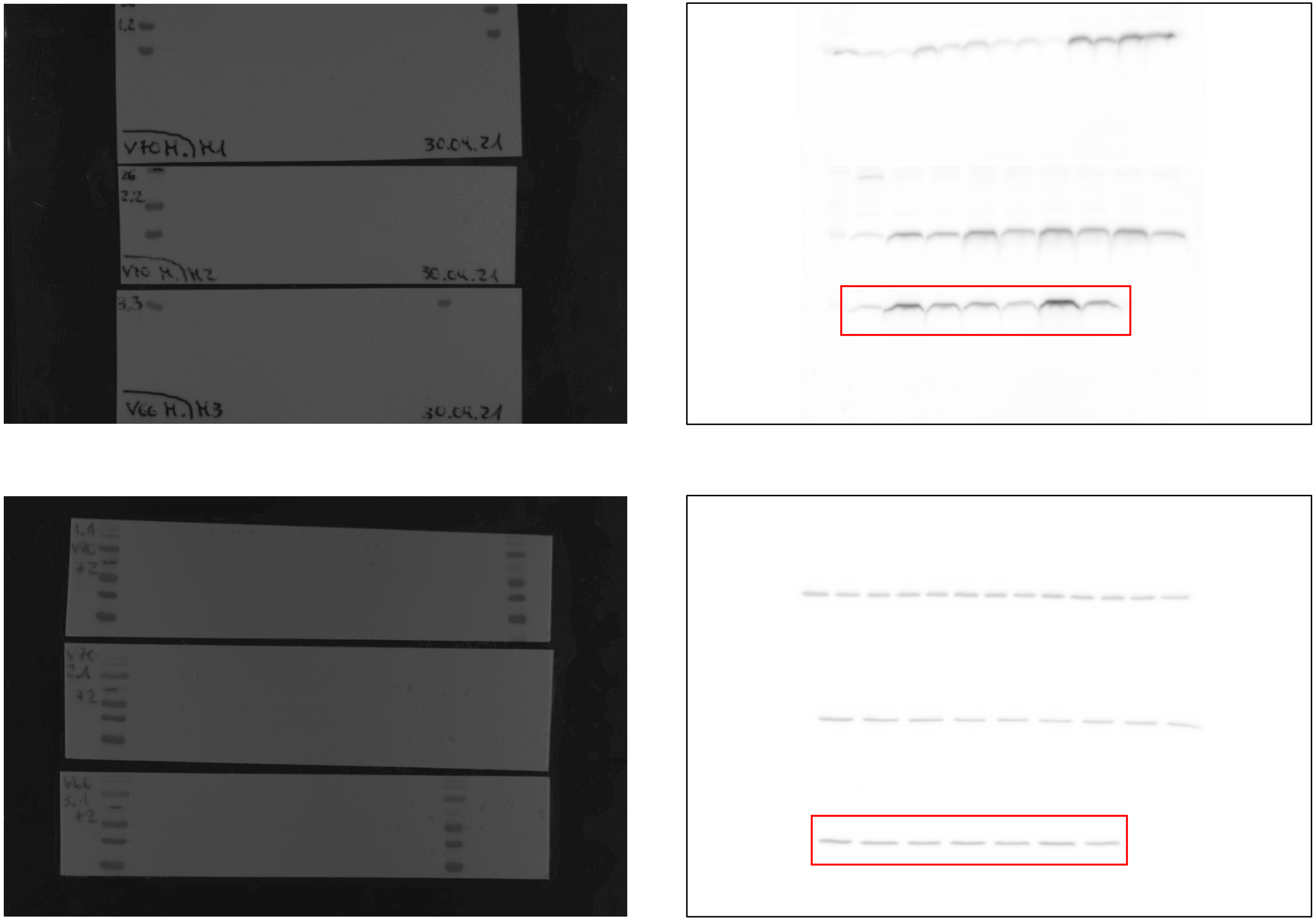

NOXA

ß-ACTIN

Suppl. Fig. 1 E

SW982/WT

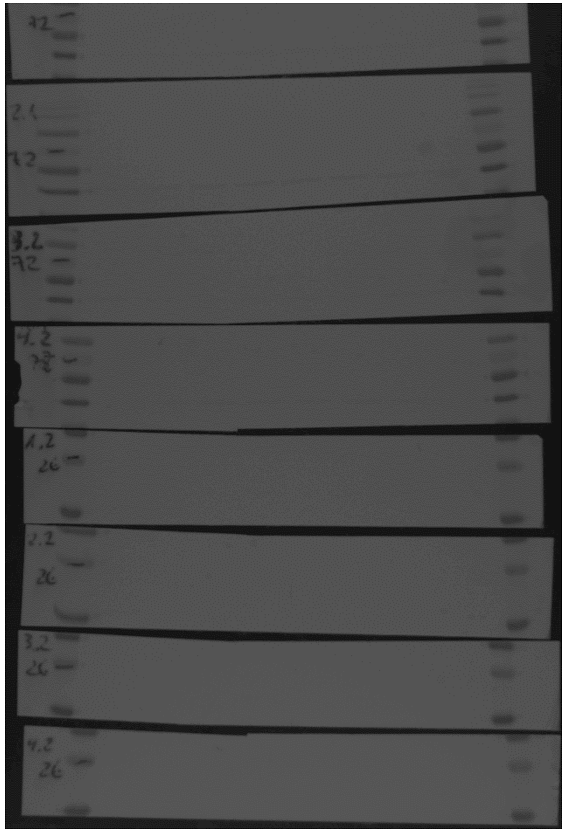

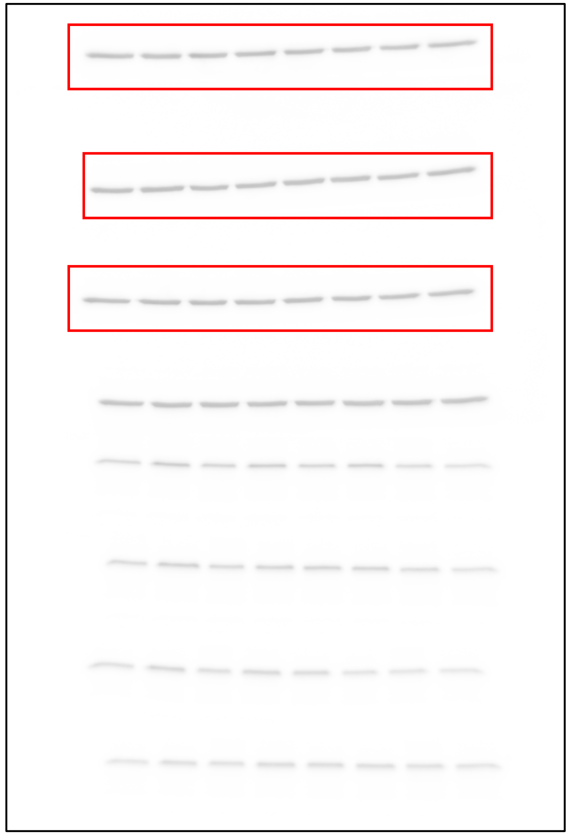

Suppl. Fig. 1 E-G

SW982/WT

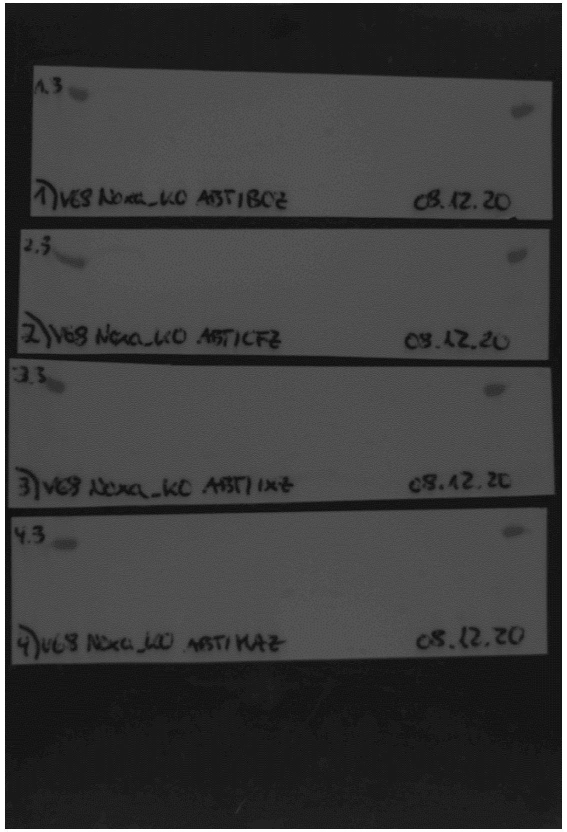

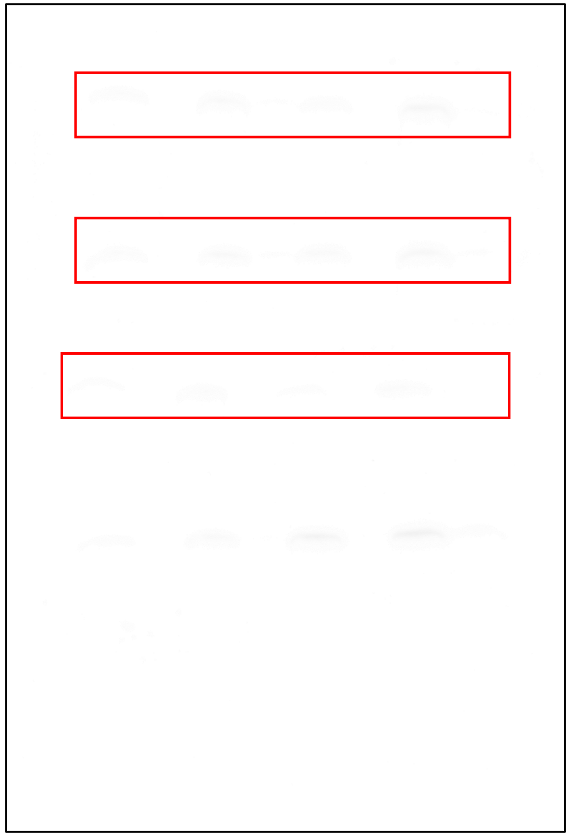

NOXA (Suppl.Fig. 1 E)

NOXA (Suppl.Fig. 1 F)

NOXA (Suppl.Fig. 1 G)

Suppl. Fig. 1 I

HCT116

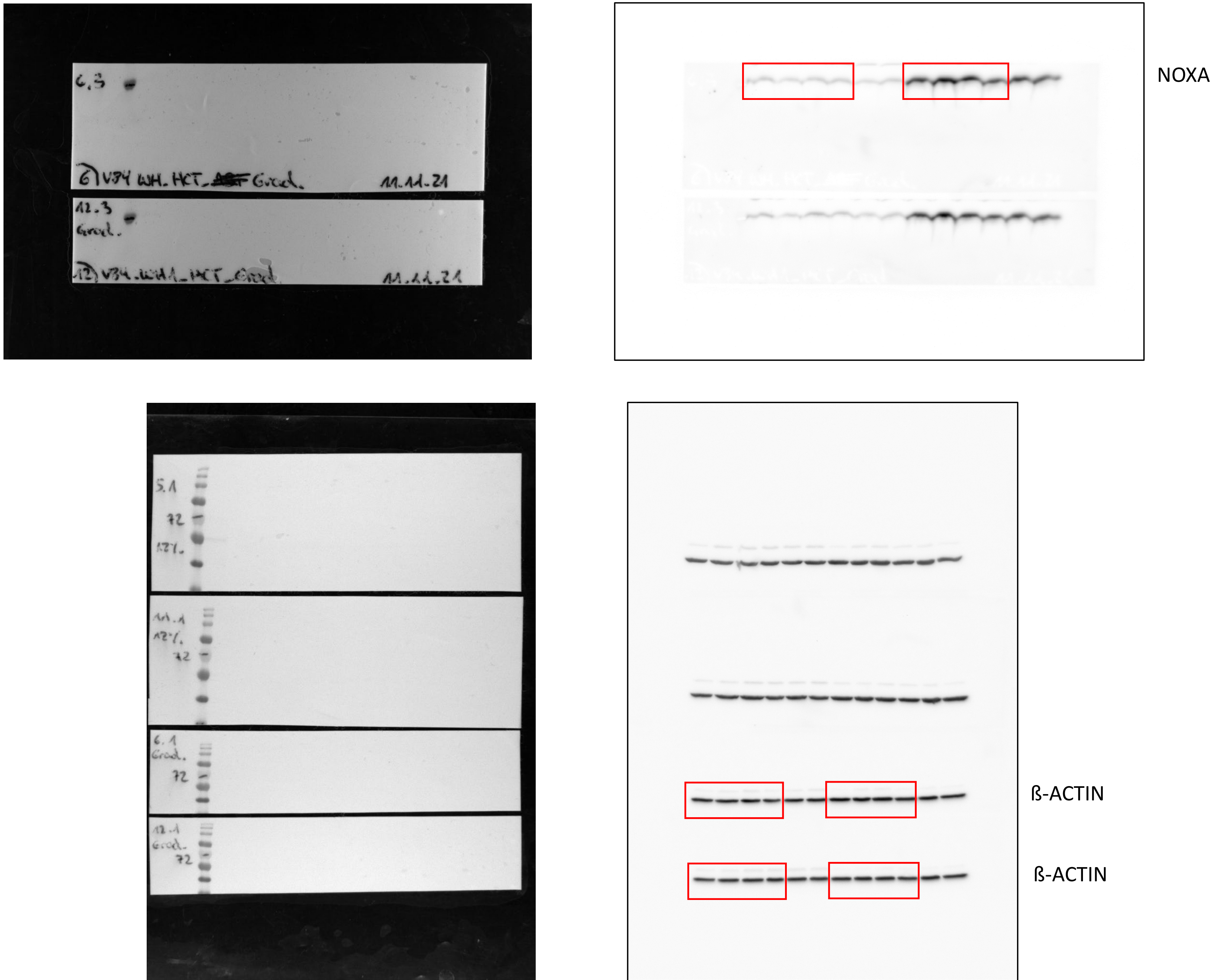

Suppl. Fig. 1 I

HCT116

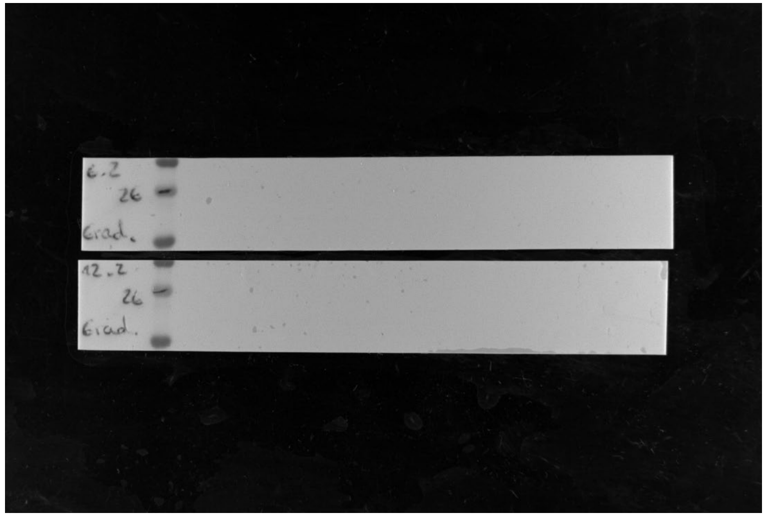

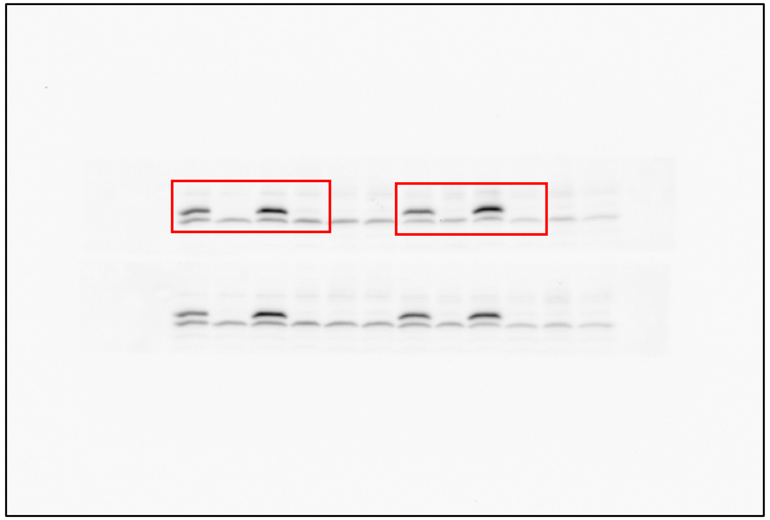

BAX

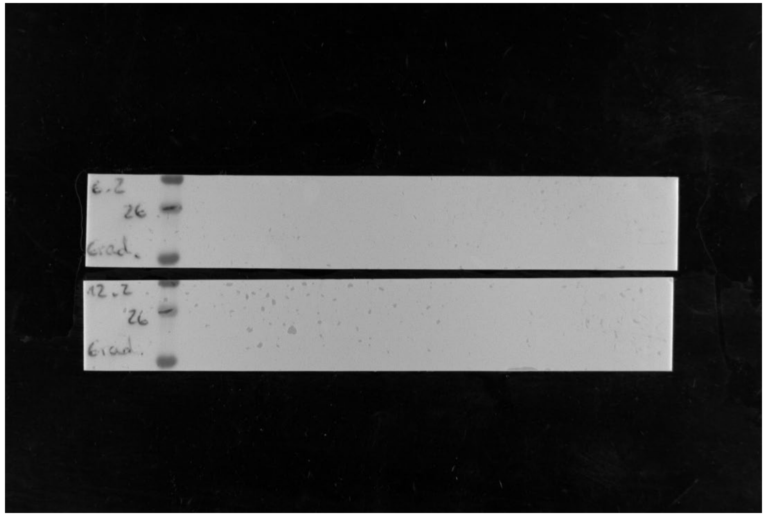

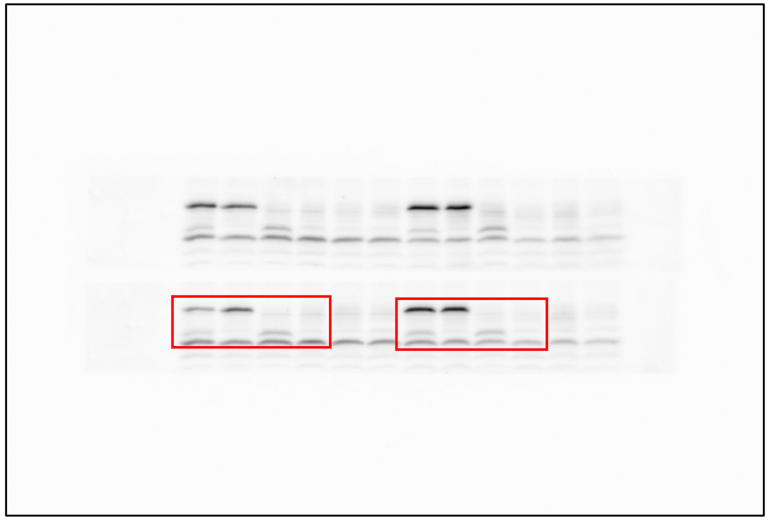

BAK

Suppl. Fig. 2 H

H1299

H1299+p53								
-	+	-	+	-	+	-	+	15 µM ABT-199
-	-	+	+	-	-	-	-	5 nM Bortezomib
-	-	-	-	+	+	-	-	5 nM Carfilzomib
-	-	-	-	-	-	+	+	5 nM Ixazomib
+	+	+	+	+	+	+	+	10 µM QVD-OPh

kDa

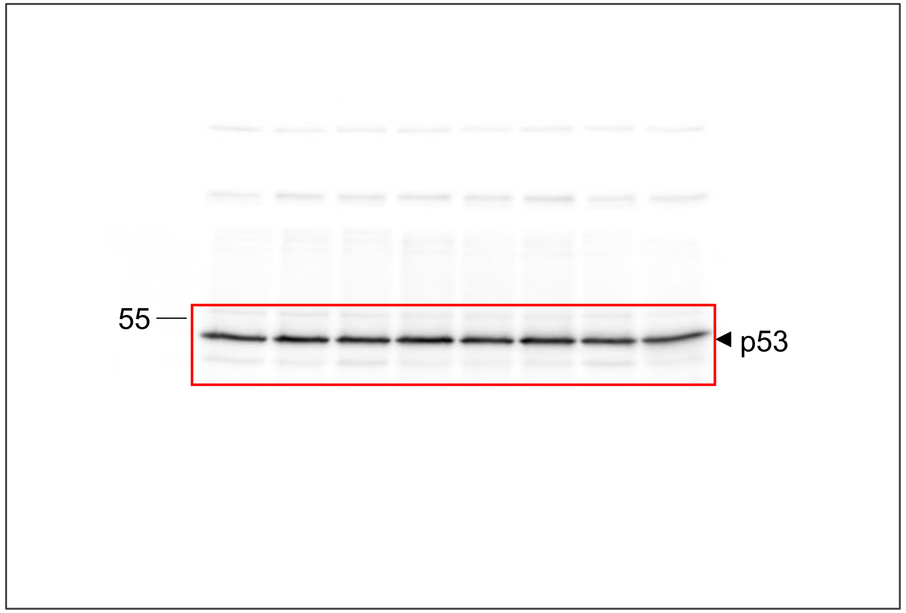

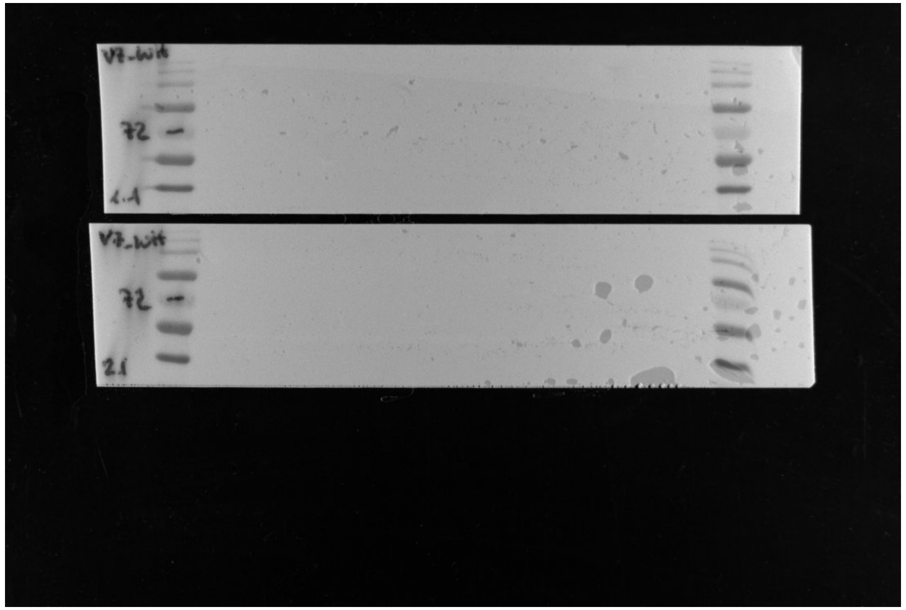

Suppl. Fig. 2 H

H1299

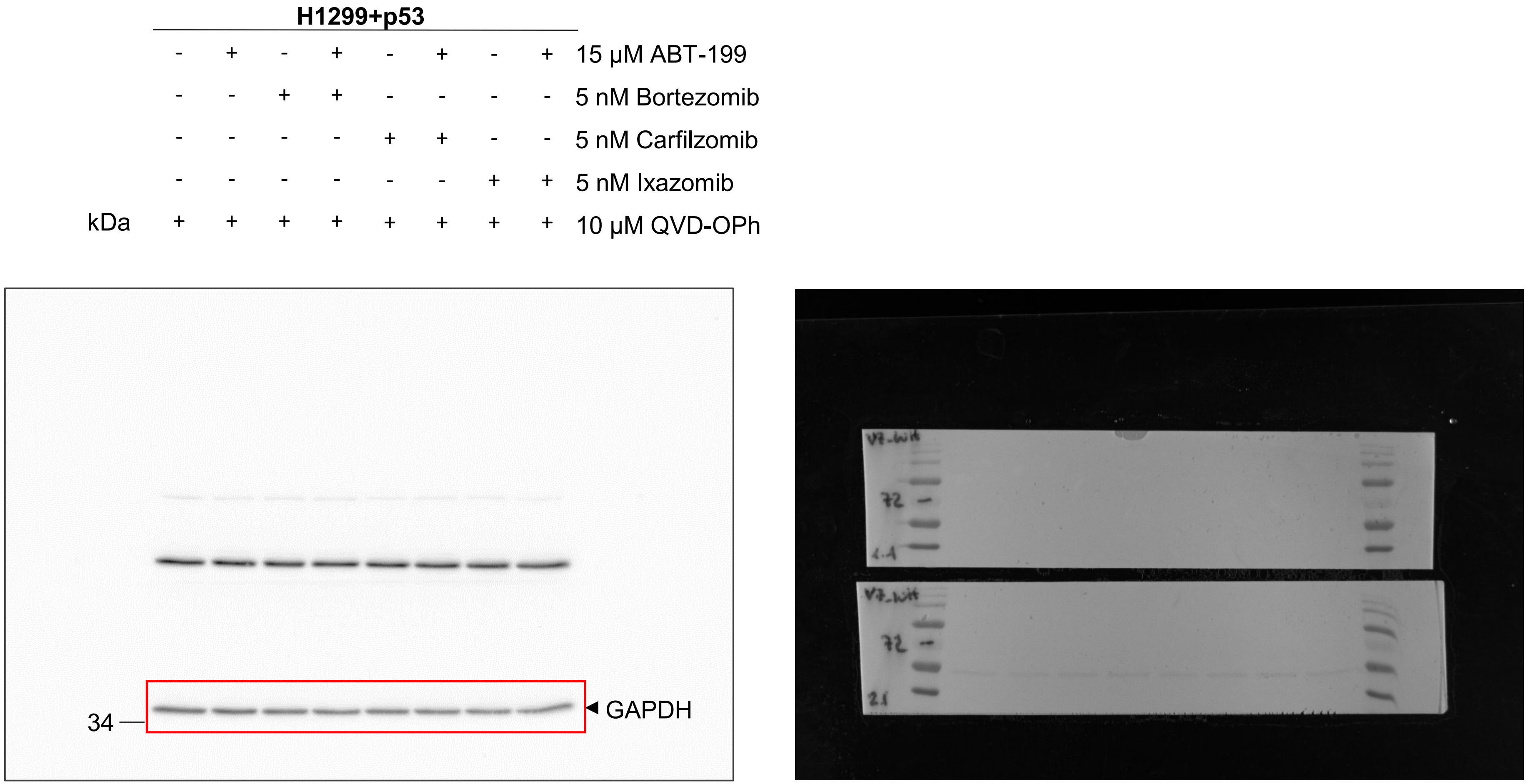

Suppl. Fig. 2 H

H1299

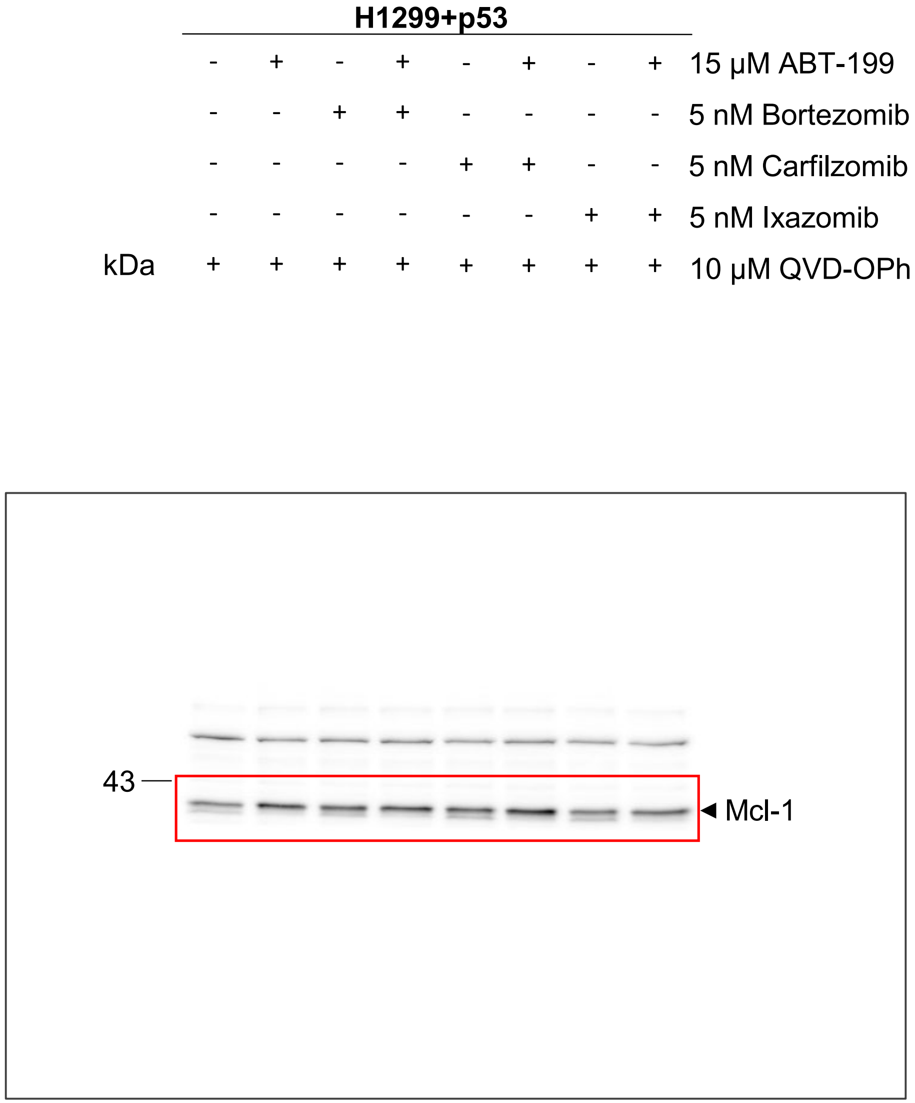

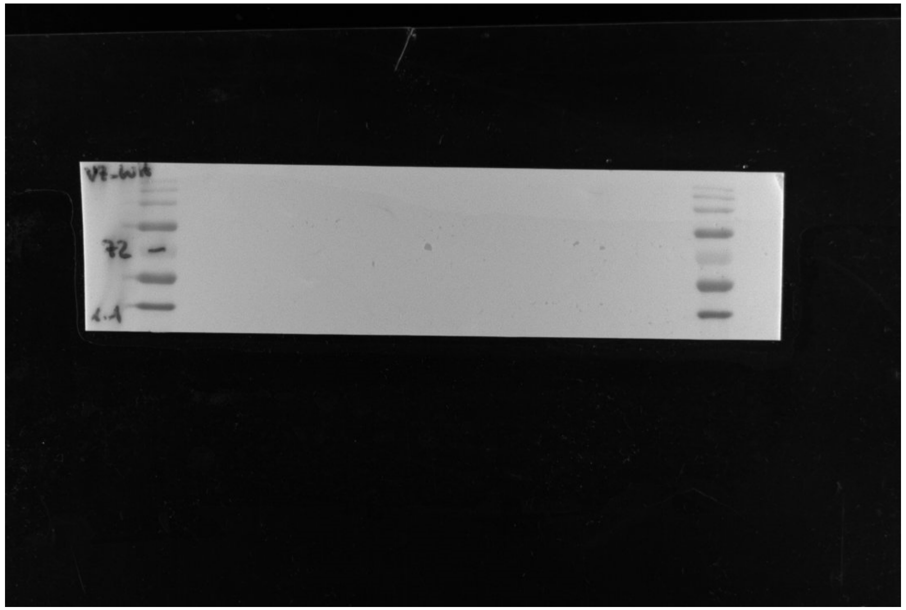

Suppl. Fig. 2 H

H1299

H1299+p53								
-	+	-	+	-	+	-	+	15 µM ABT-199
-	-	+	+	-	-	-	-	5 nM Bortezomib
-	-	-	-	+	+	-	-	5 nM Carfilzomib
-	-	-	-	-	-	+	+	5 nM Ixazomib
+	+	+	+	+	+	+	+	10 µM QVD-OPh

kDa

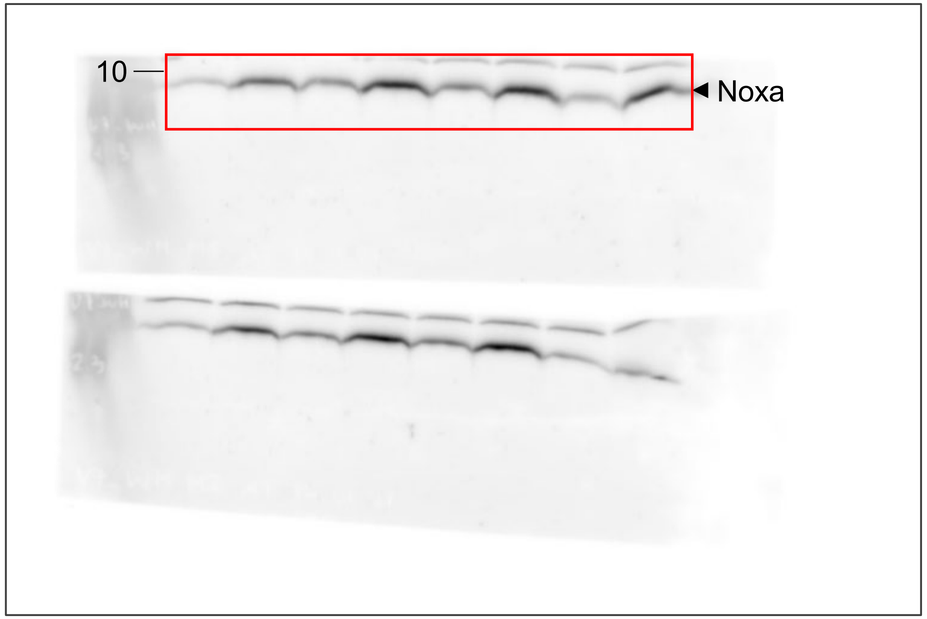

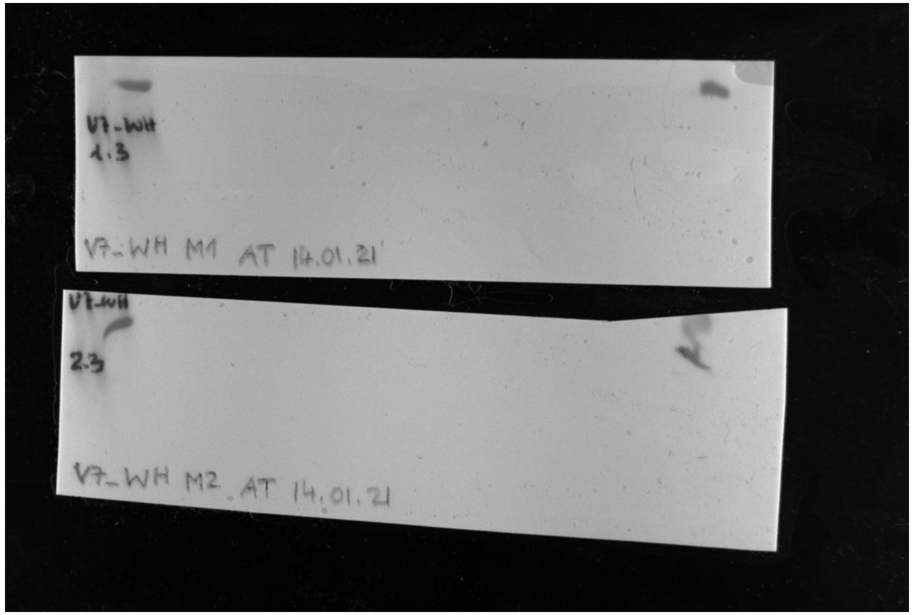

Suppl. Fig. 2 H

H1299

	H1299+p53								
	-	+	-	+	-	+	-	+	15 µM ABT-199
	-	-	+	+	-	-	-	-	5 nM Bortezomib
	-	-	-	-	+	+	-	-	5 nM Carfilzomib
	-	-	-	-	-	-	+	+	5 nM Ixazomib
kDa	+	+	+	+	+	+	+	+	10 µM QVD-OPh

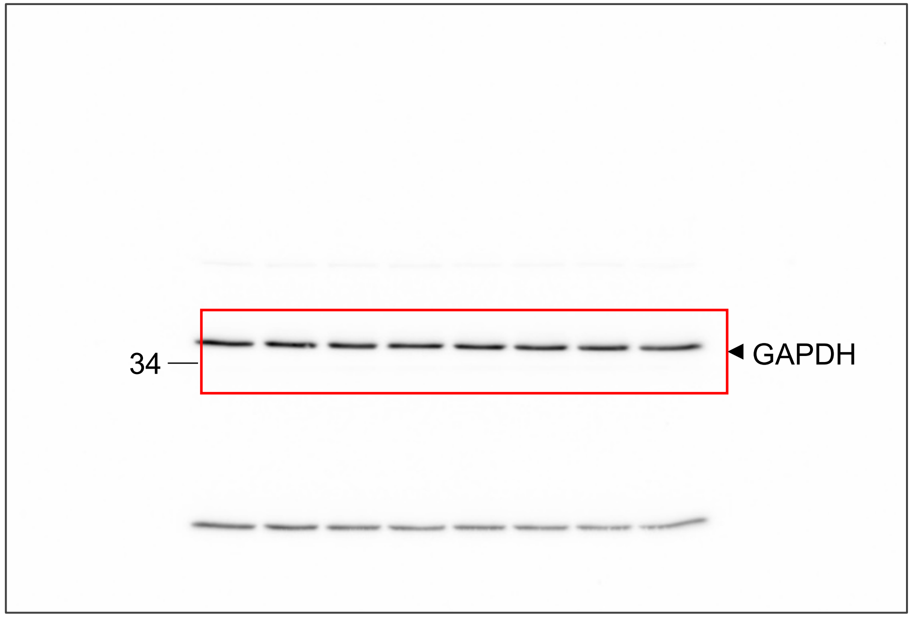

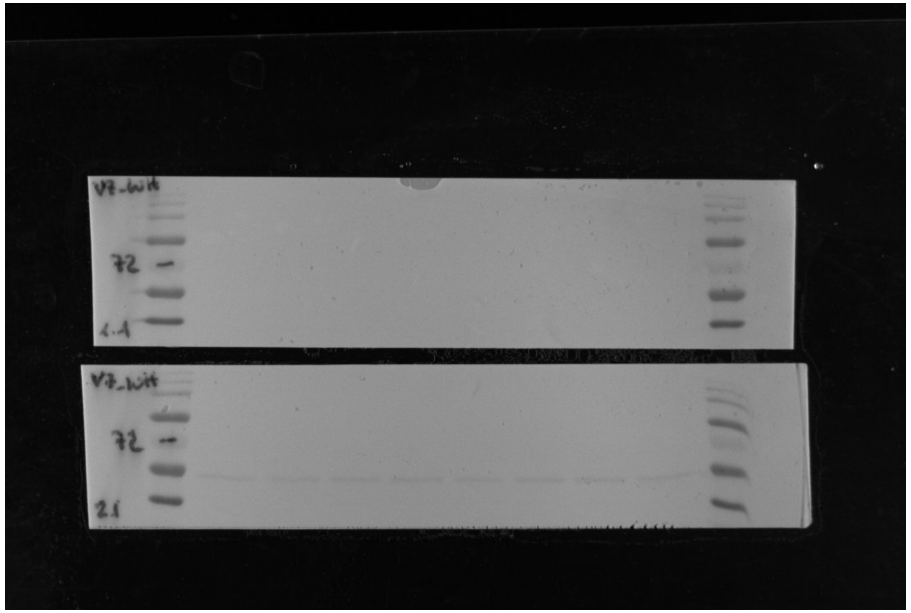

Suppl. Fig. 2 H

H1299

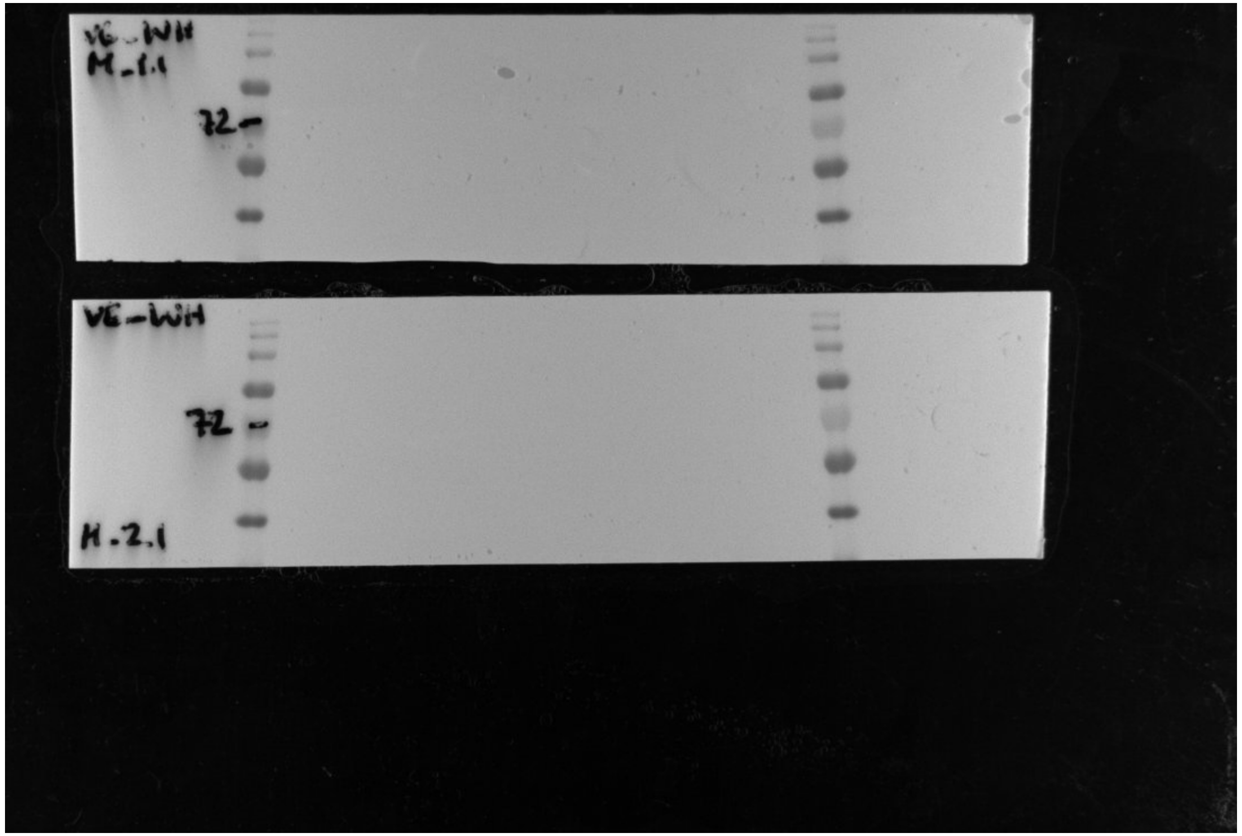

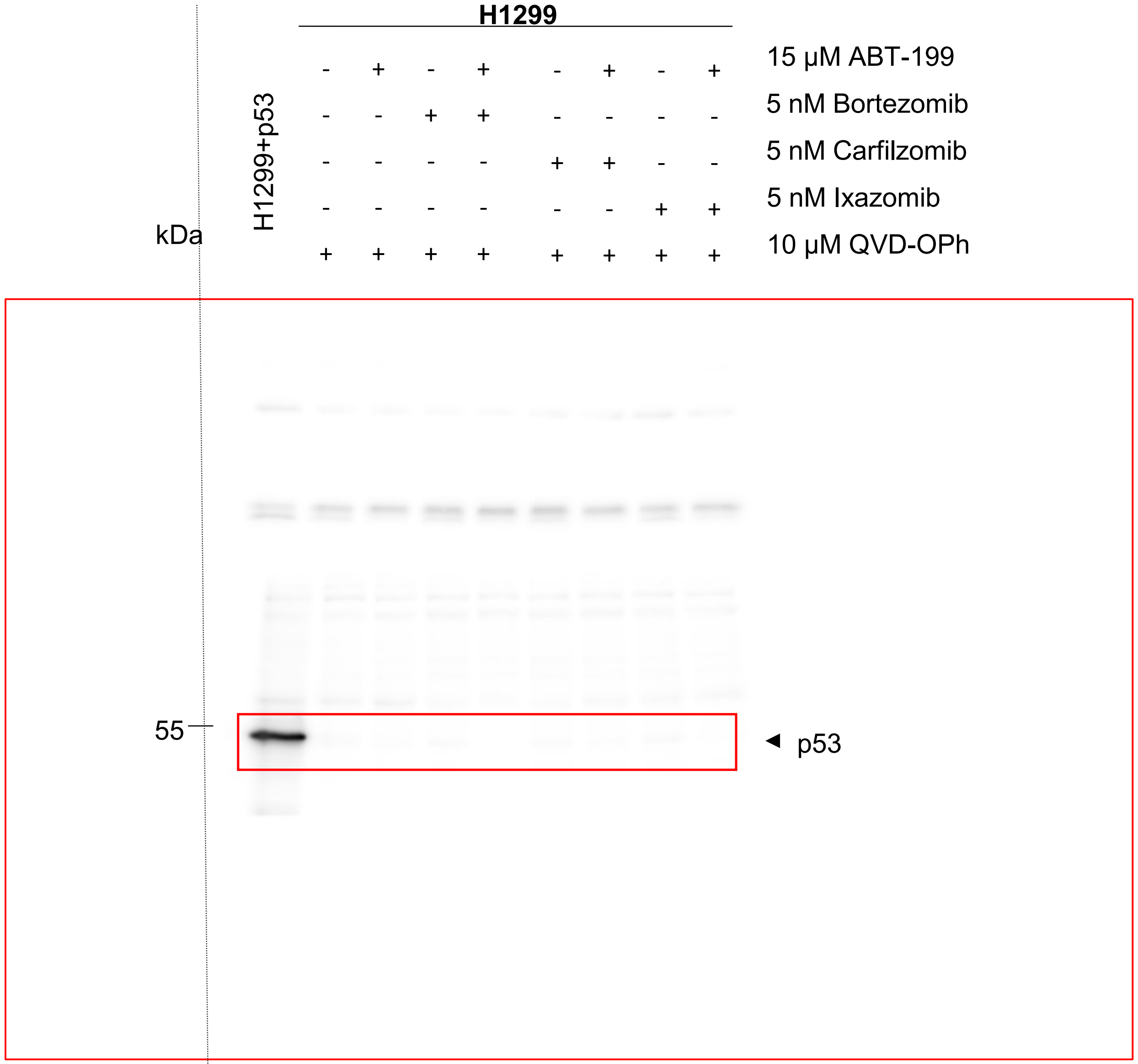

Suppl. Fig. 2 H

H1299

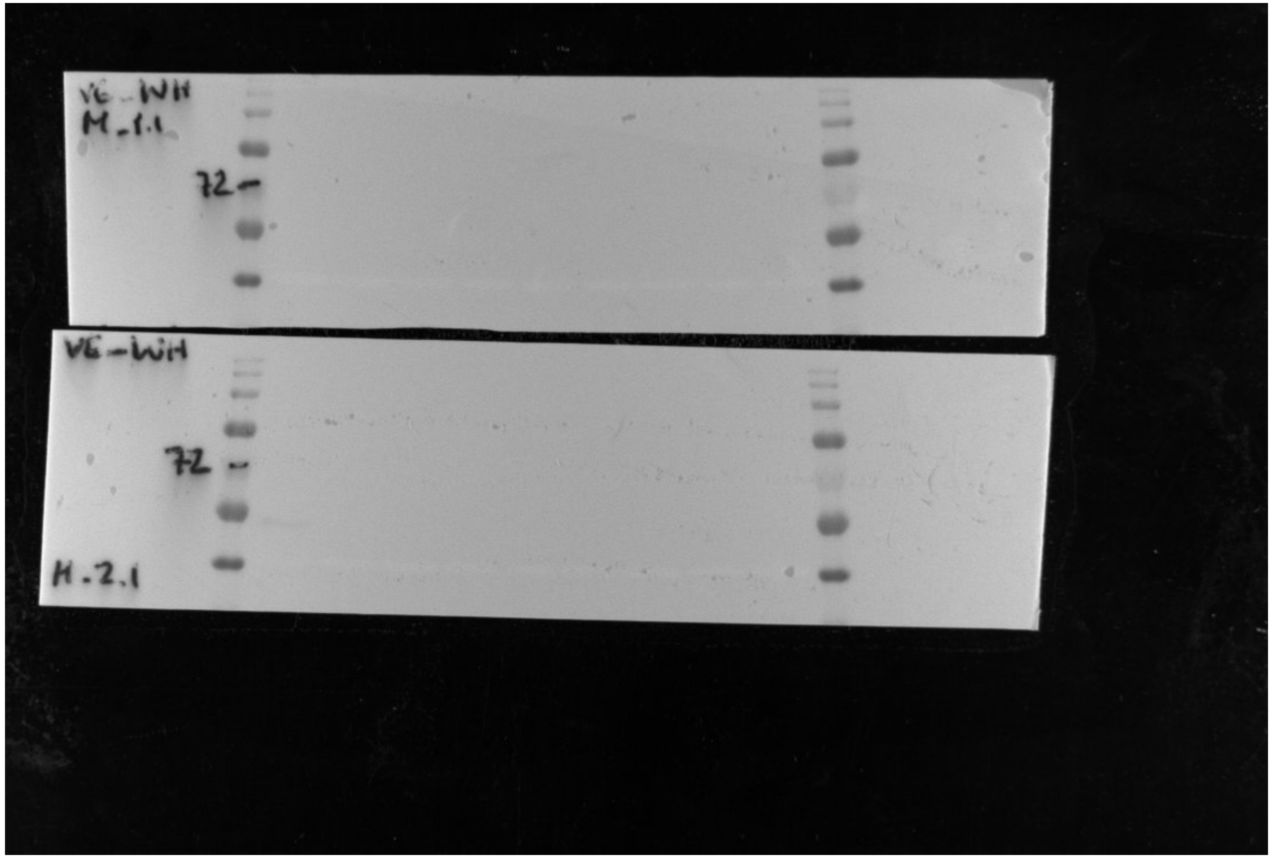

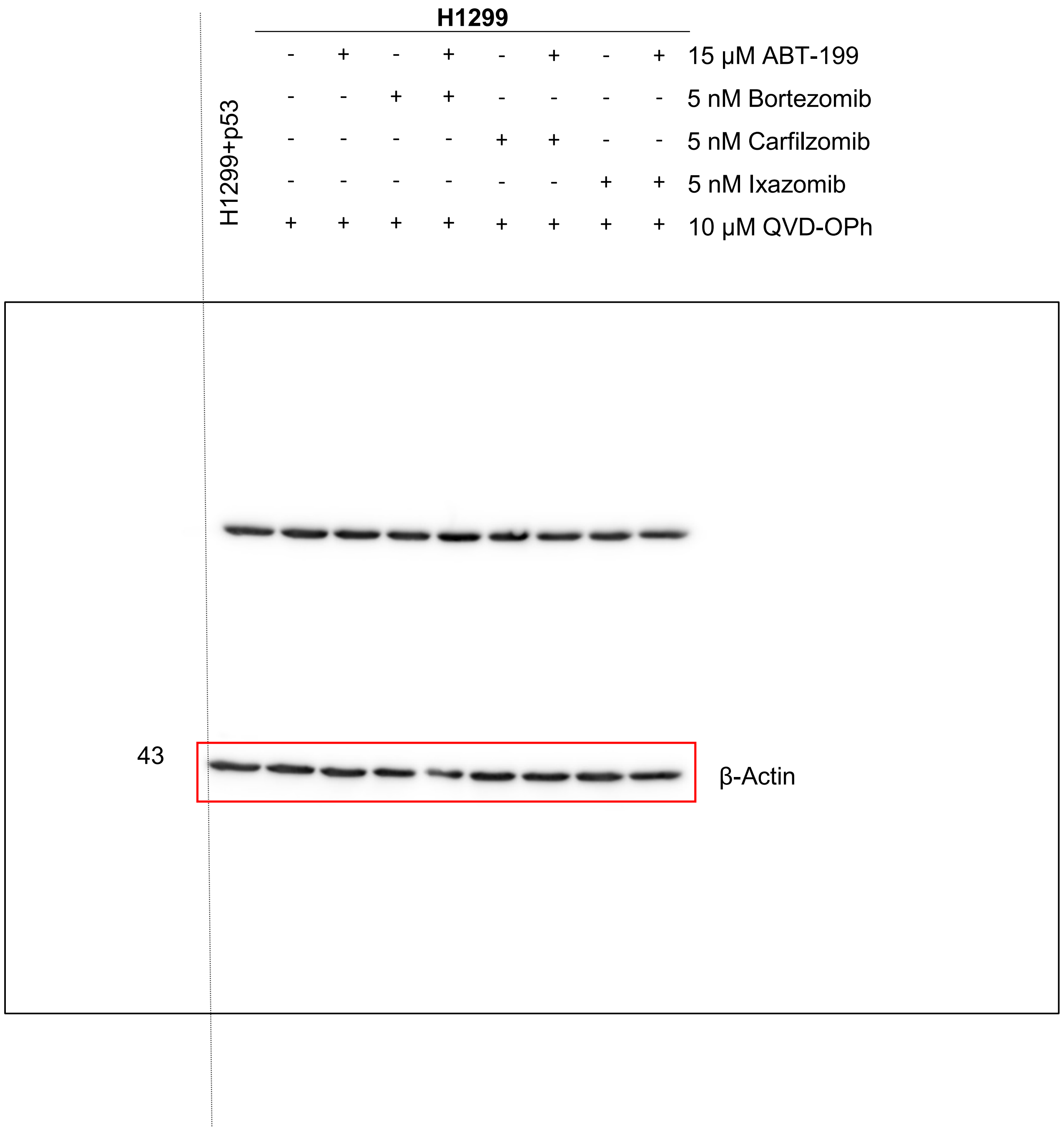

Suppl. Fig. 2 H

H1299

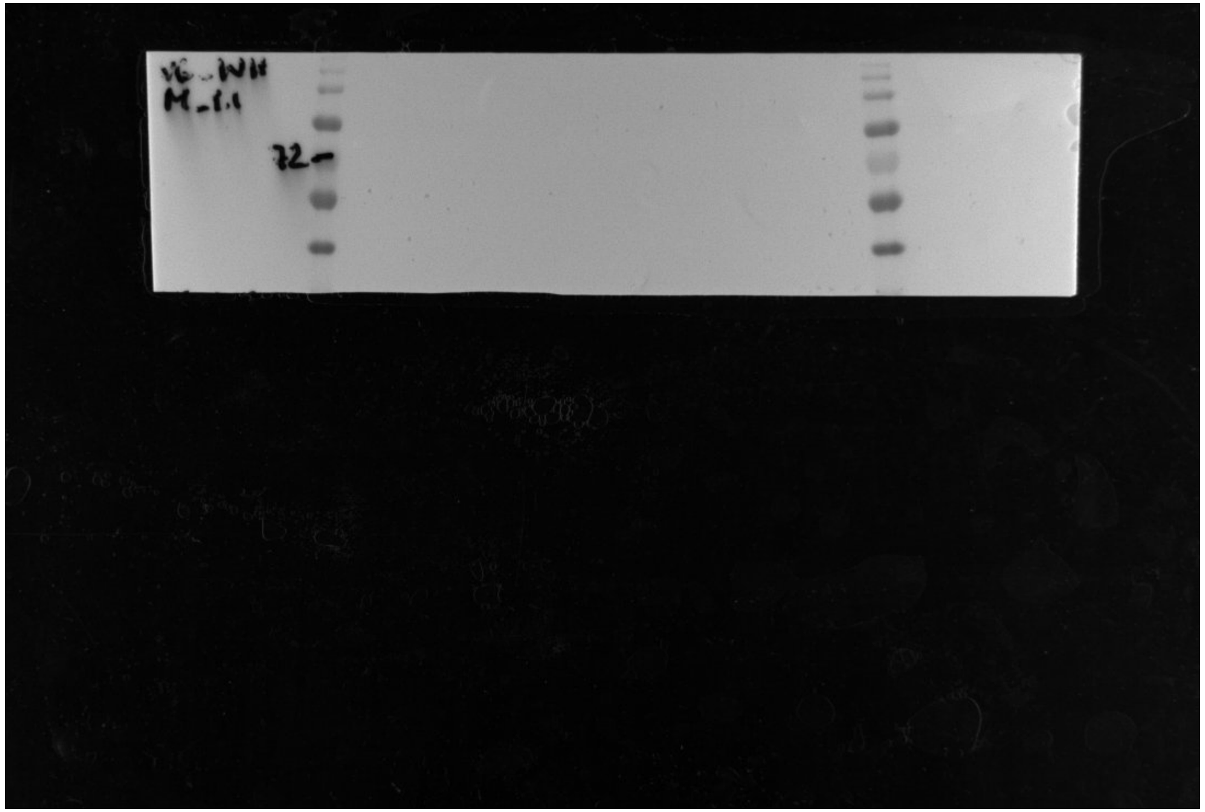

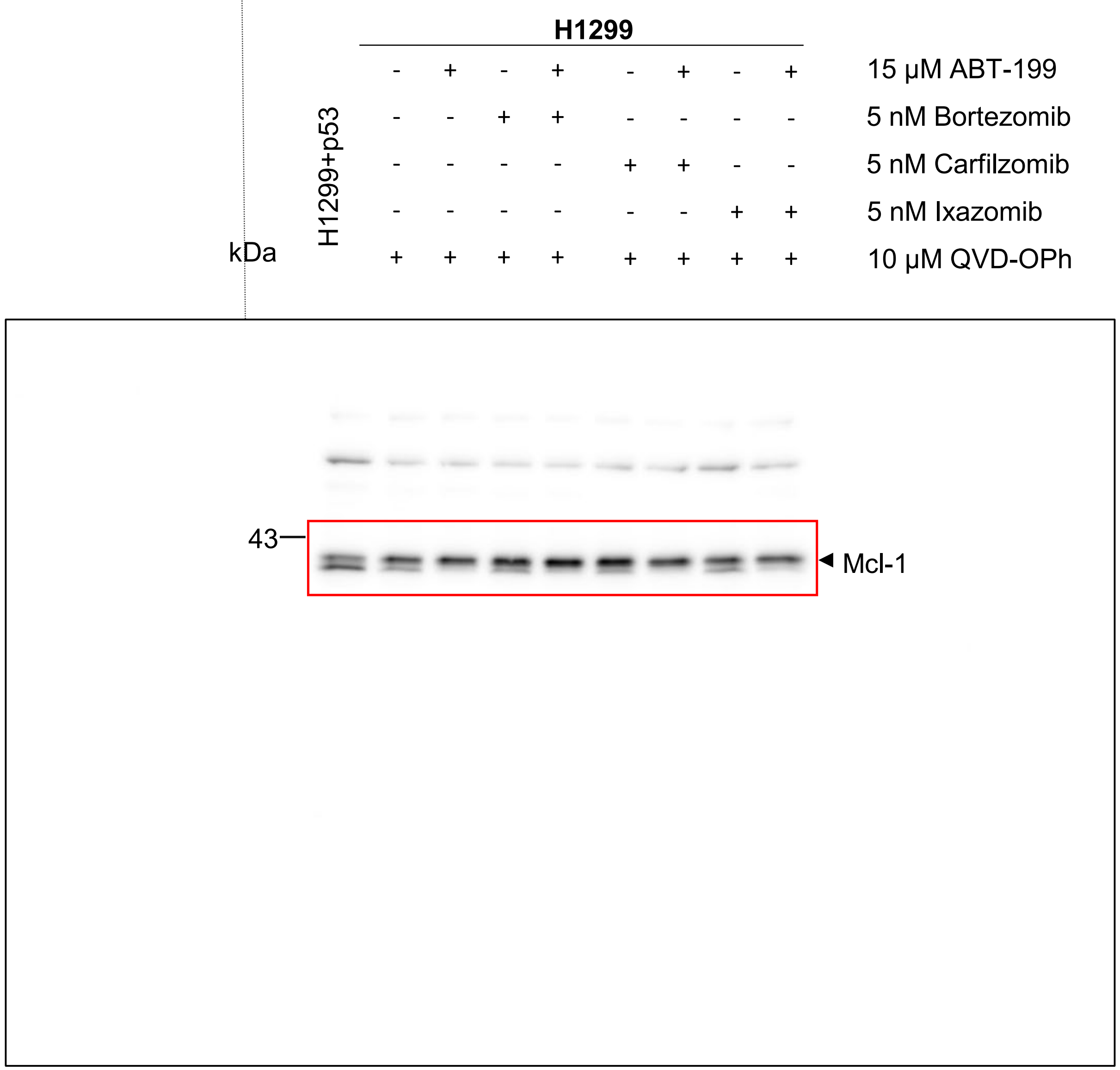

Suppl. Fig. 2 H

H1299

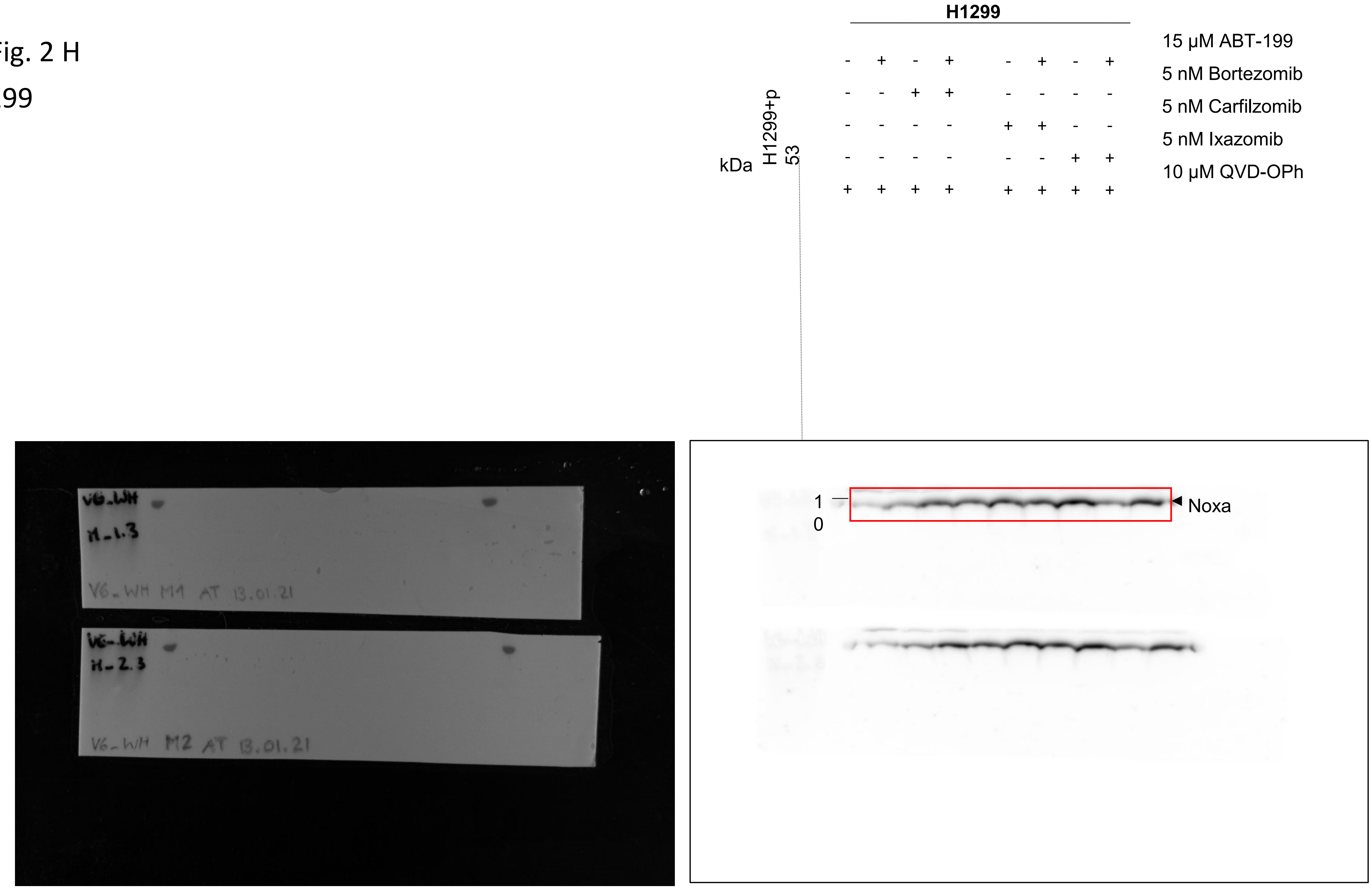

Suppl. Fig. 2 H

H1299

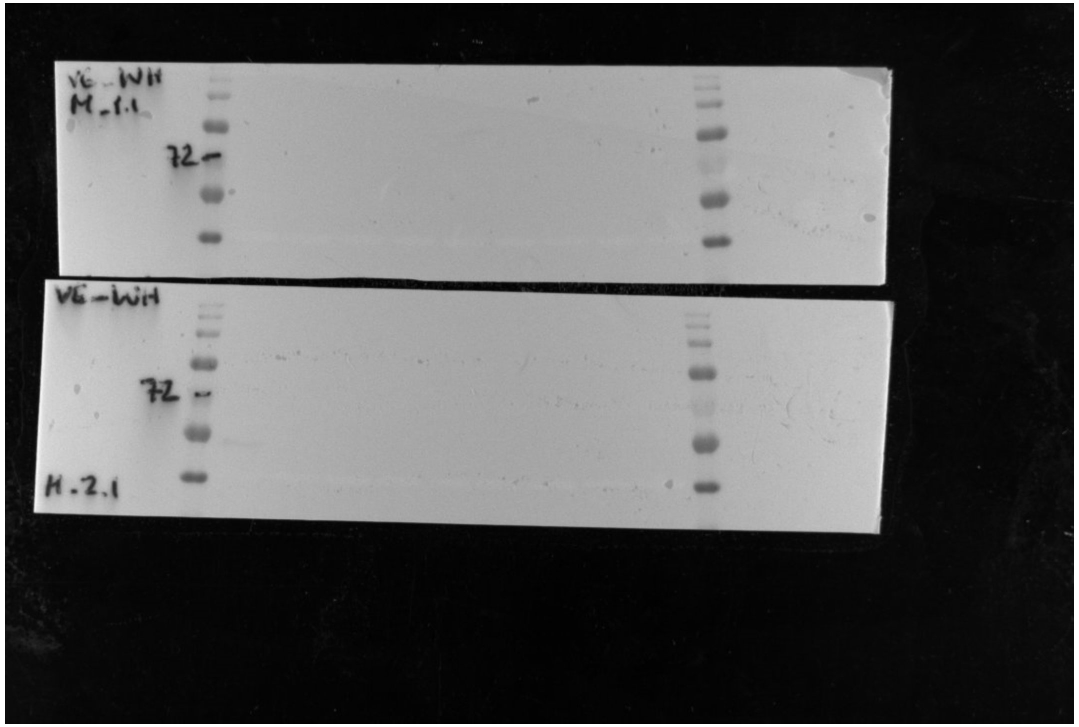

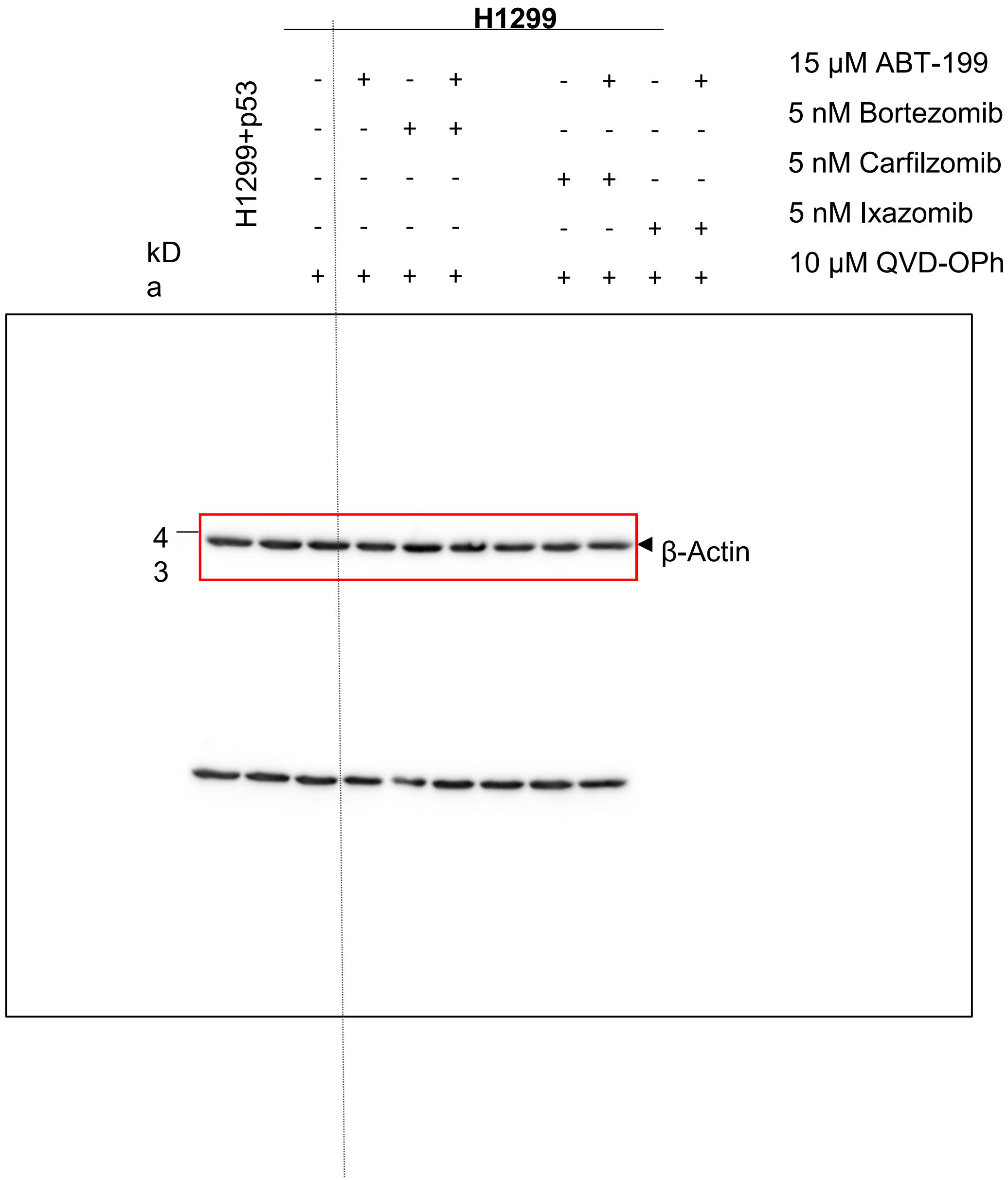

Suppl. Fig. 3 A

SW982/WT

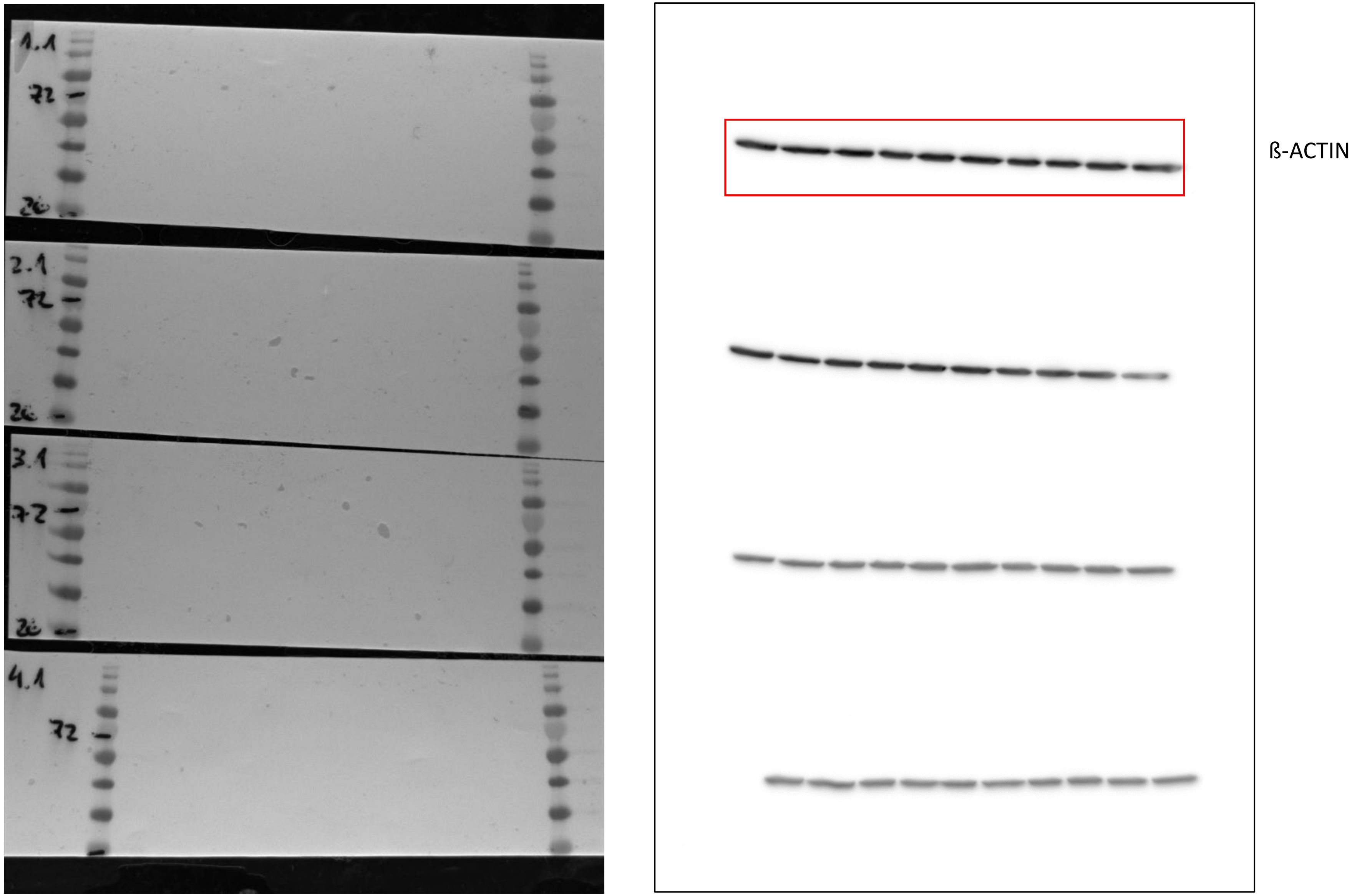

Suppl. Fig. 3 A

SW982/WT

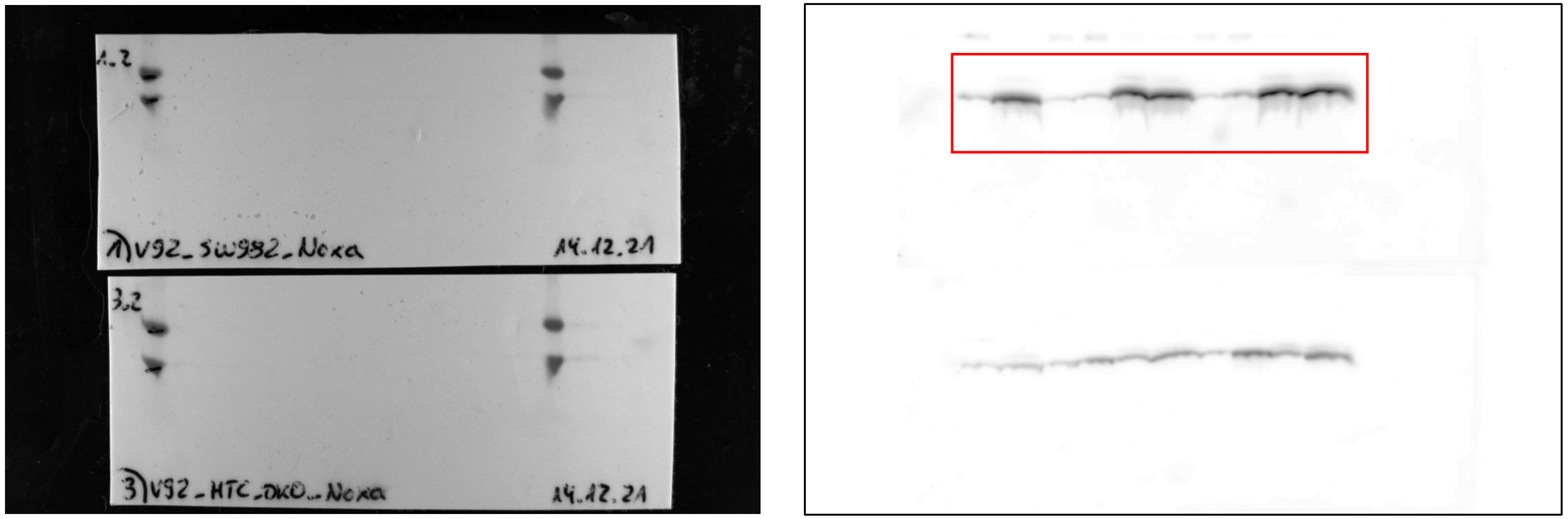

NOXA

Suppl. Fig. 3 B

SW982/WT

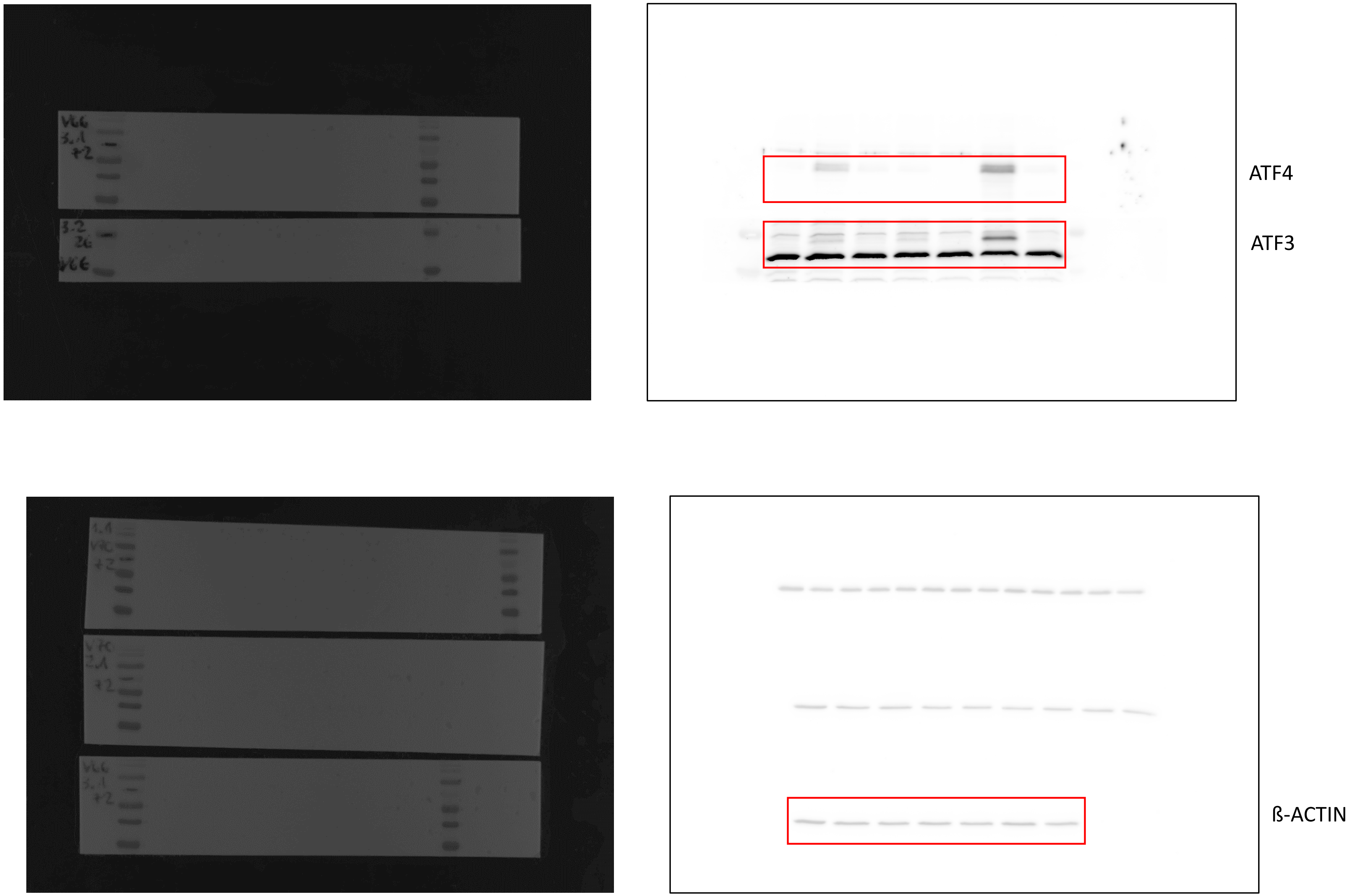

Supplement: Supplementary file 8 — original western blots [file 41420_2022_1009_MOESM8_ESM.pdf]
